# Supplementary figures and images for: Derivation and external validation of a risk score for predicting HIV-associated tuberculosis to support case finding and preventive therapy scale-up: A cohort study
Source: PLoS Med. 2021 Sep 7;18(9):e1003739. doi: 10.1371/journal.pmed.1003739 (PMC8454974; doi:10.1371/journal.pmed.1003739)

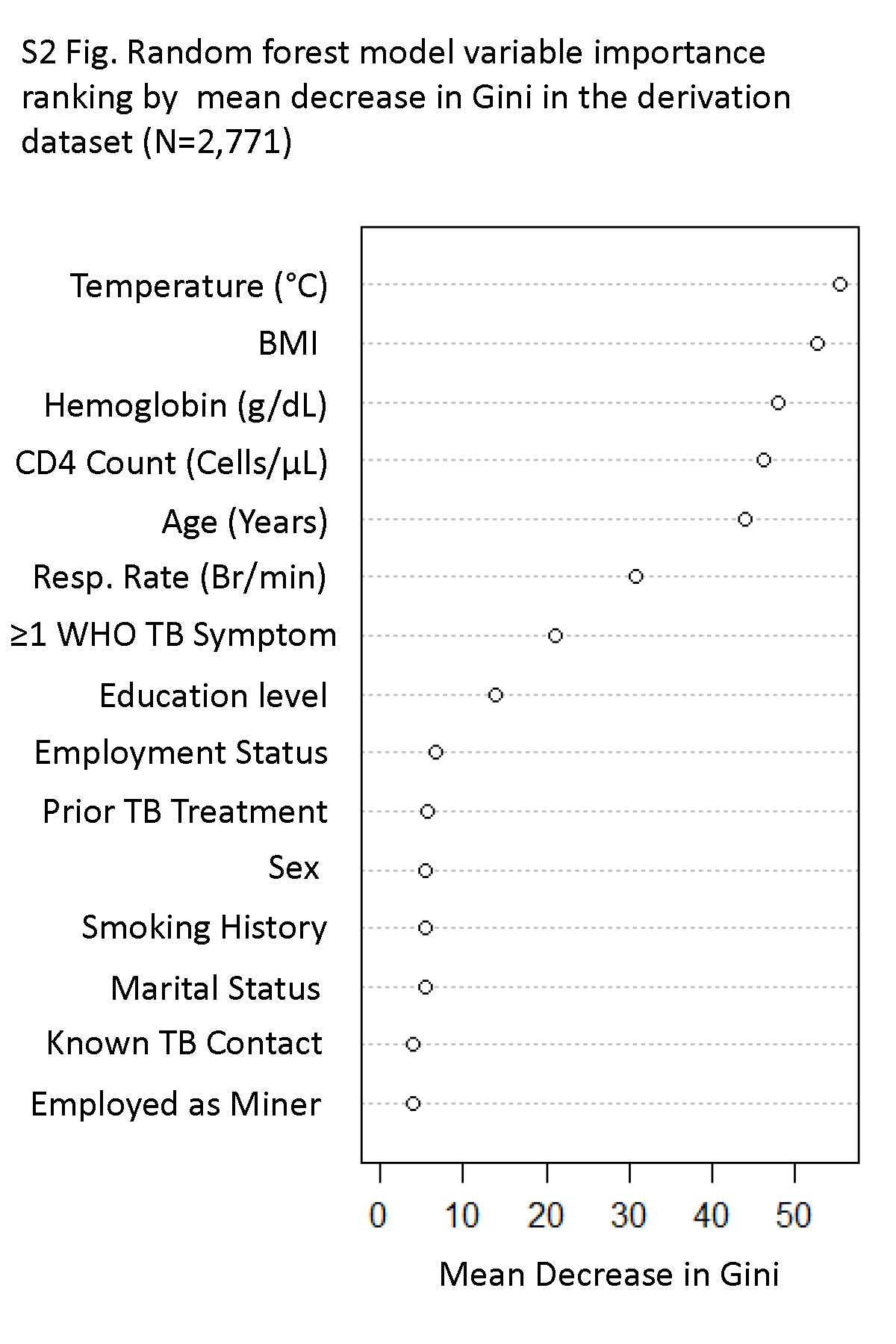

Supplement: S2 Fig — (TIF) [file pmed.1003739.s002.tif]

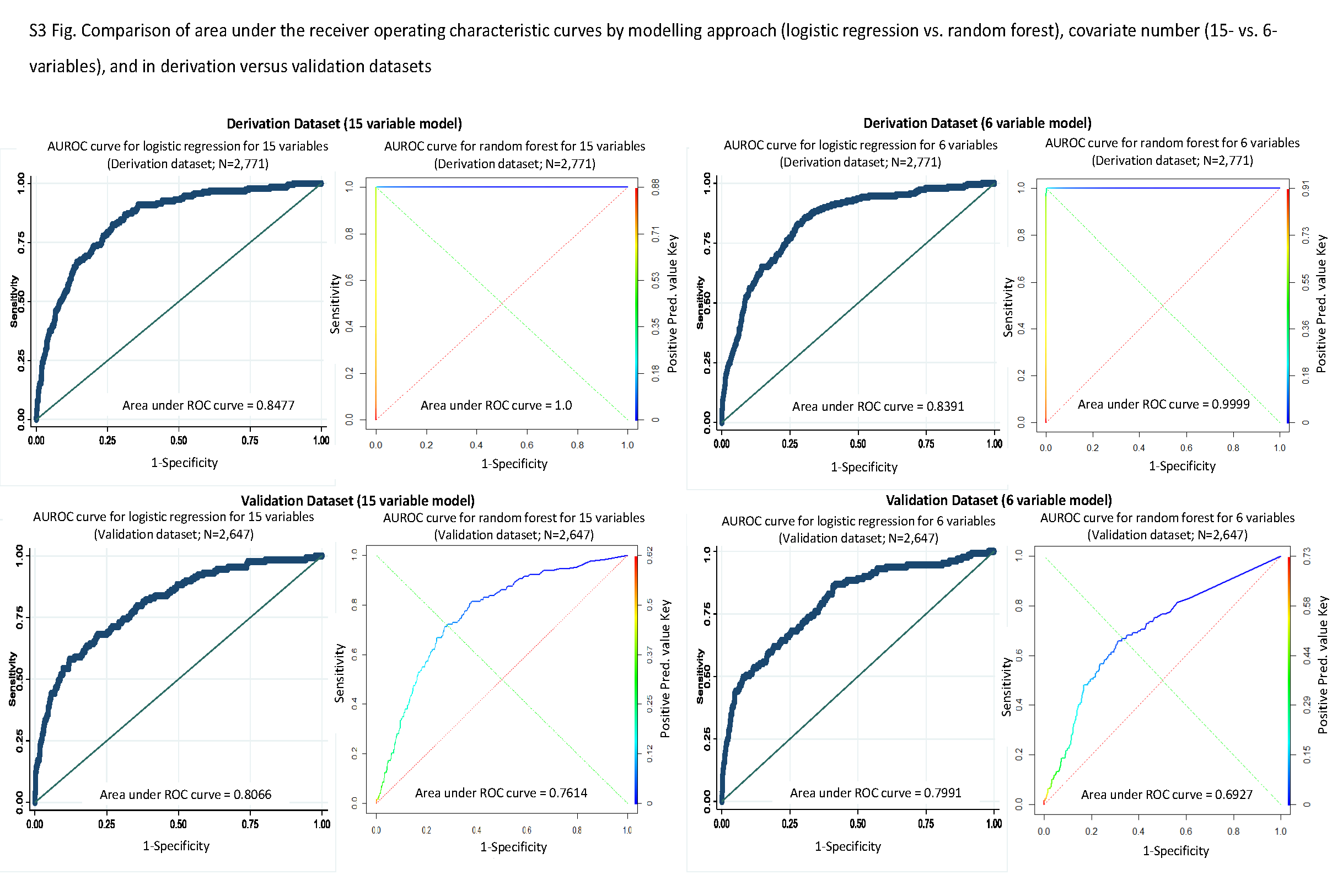

Supplement: S3 Fig — AUROC, area under the receiver operating characteristic. (TIF) [file pmed.1003739.s003.tif]

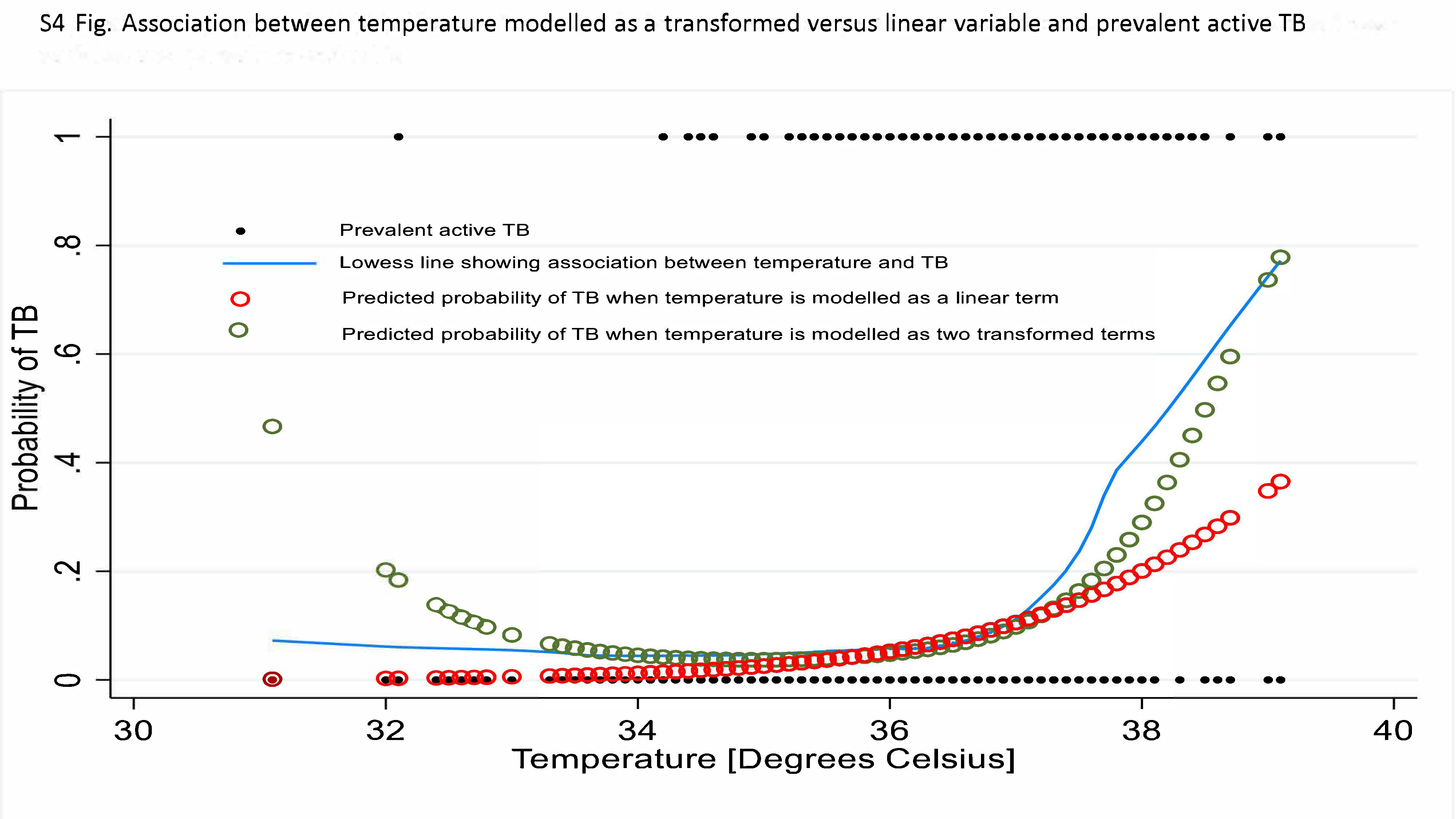

Supplement: S4 Fig — TB, tuberculosis. (TIF) [file pmed.1003739.s004.tif]

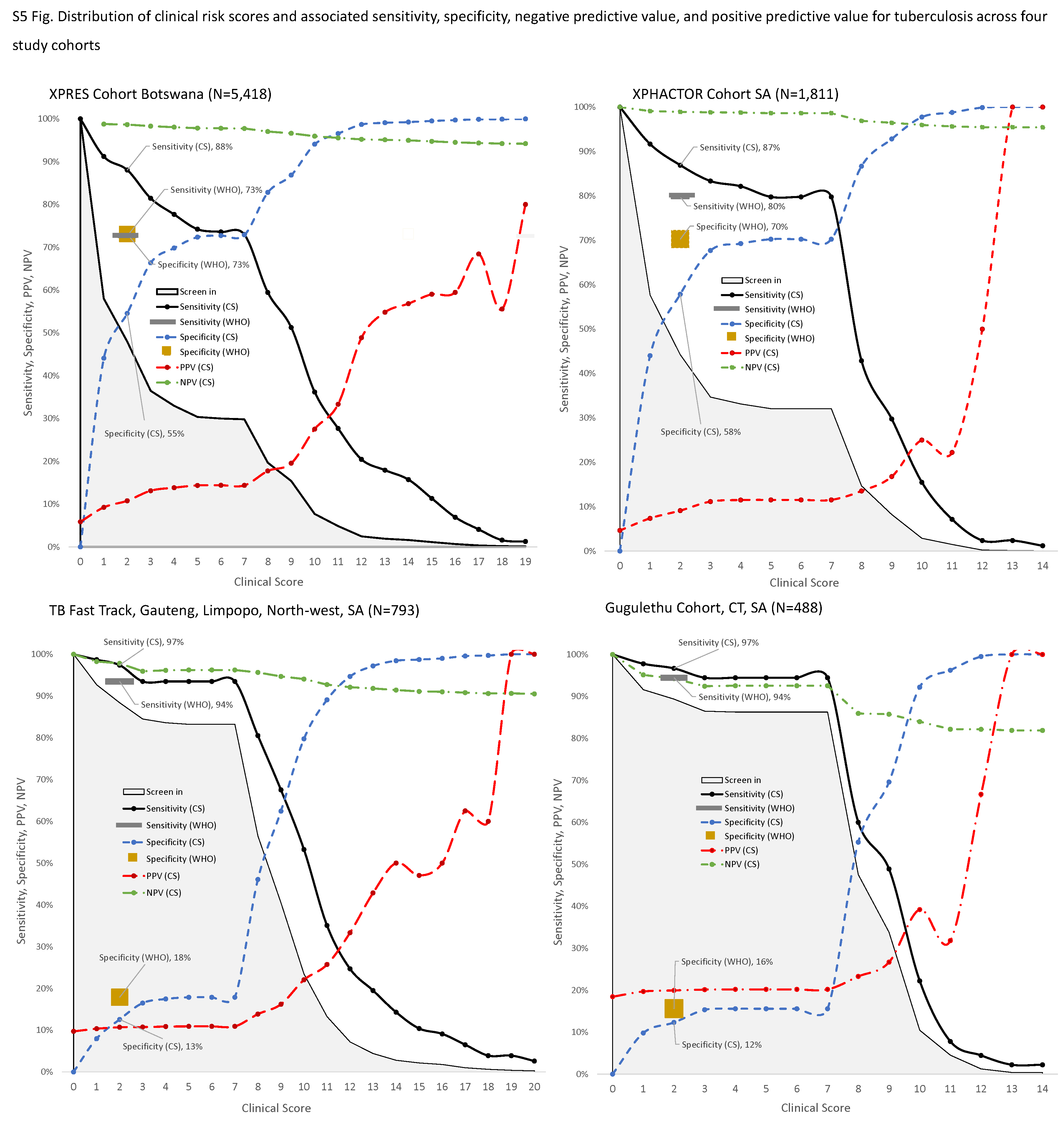

Supplement: S5 Fig — NPV, negative predictive value; TB, tuberculosis. (TIF) [file pmed.1003739.s005.tif]

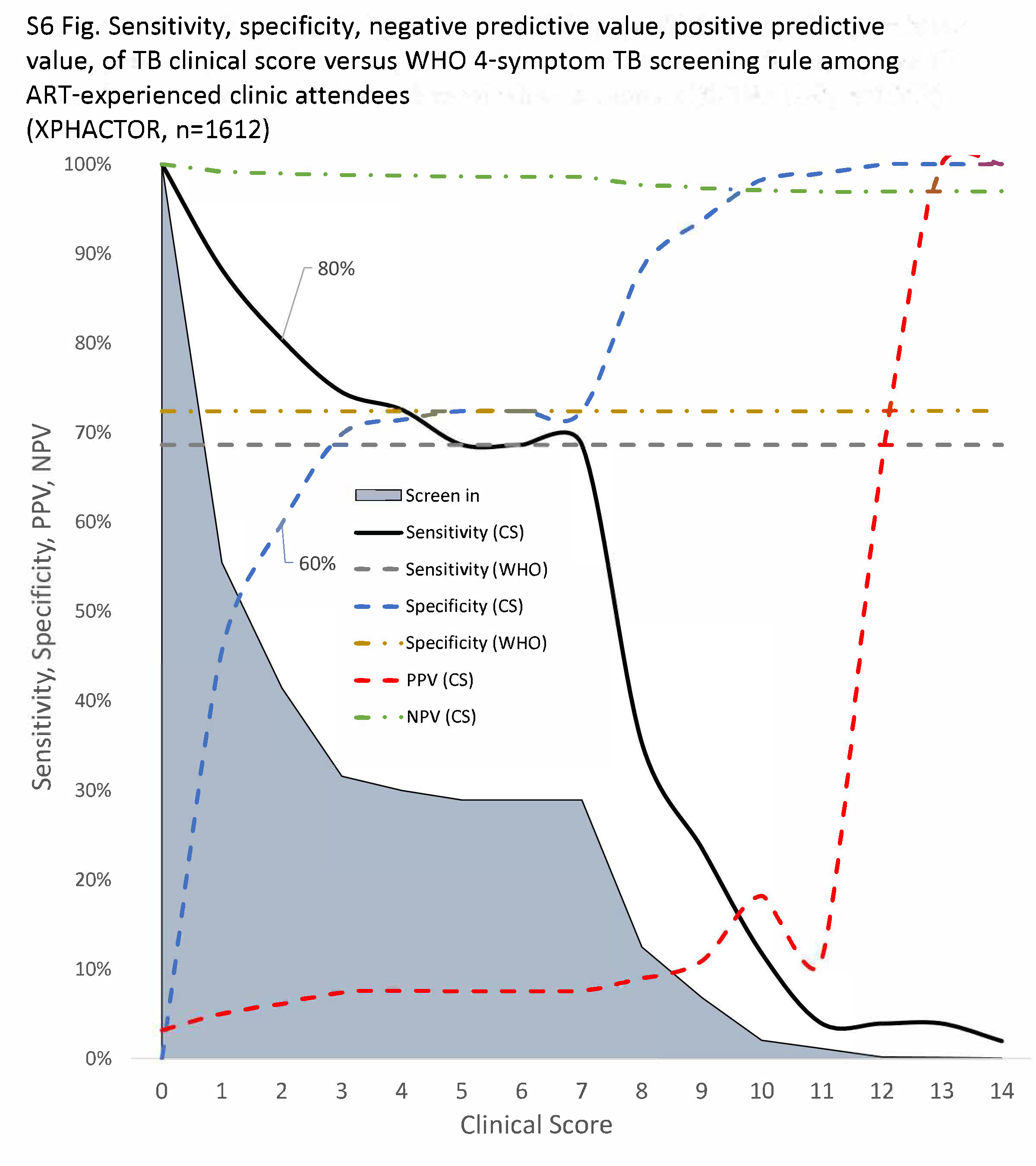

Supplement: S6 Fig — ART, antiretroviral therapy; NPV, negative predictive value; PPV, positive predictive value; TB, tuberculosis; WHO, World Health Organization. (TIF) [file pmed.1003739.s006.tif]

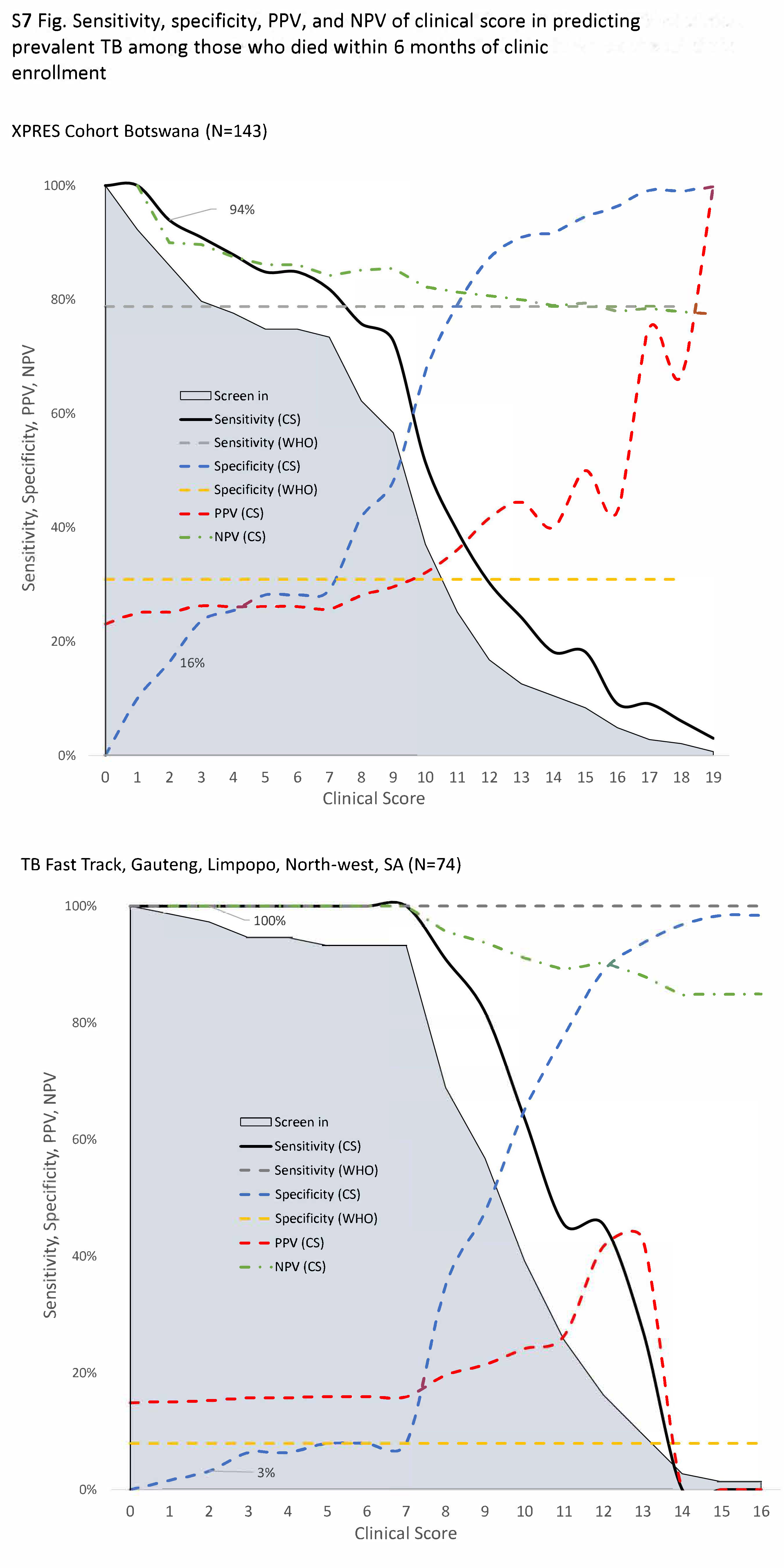

Supplement: S7 Fig — NPV, negative predictive value; PPV, positive predictive value; WHO, World Health Organization. (TIF) [file pmed.1003739.s007.tif]

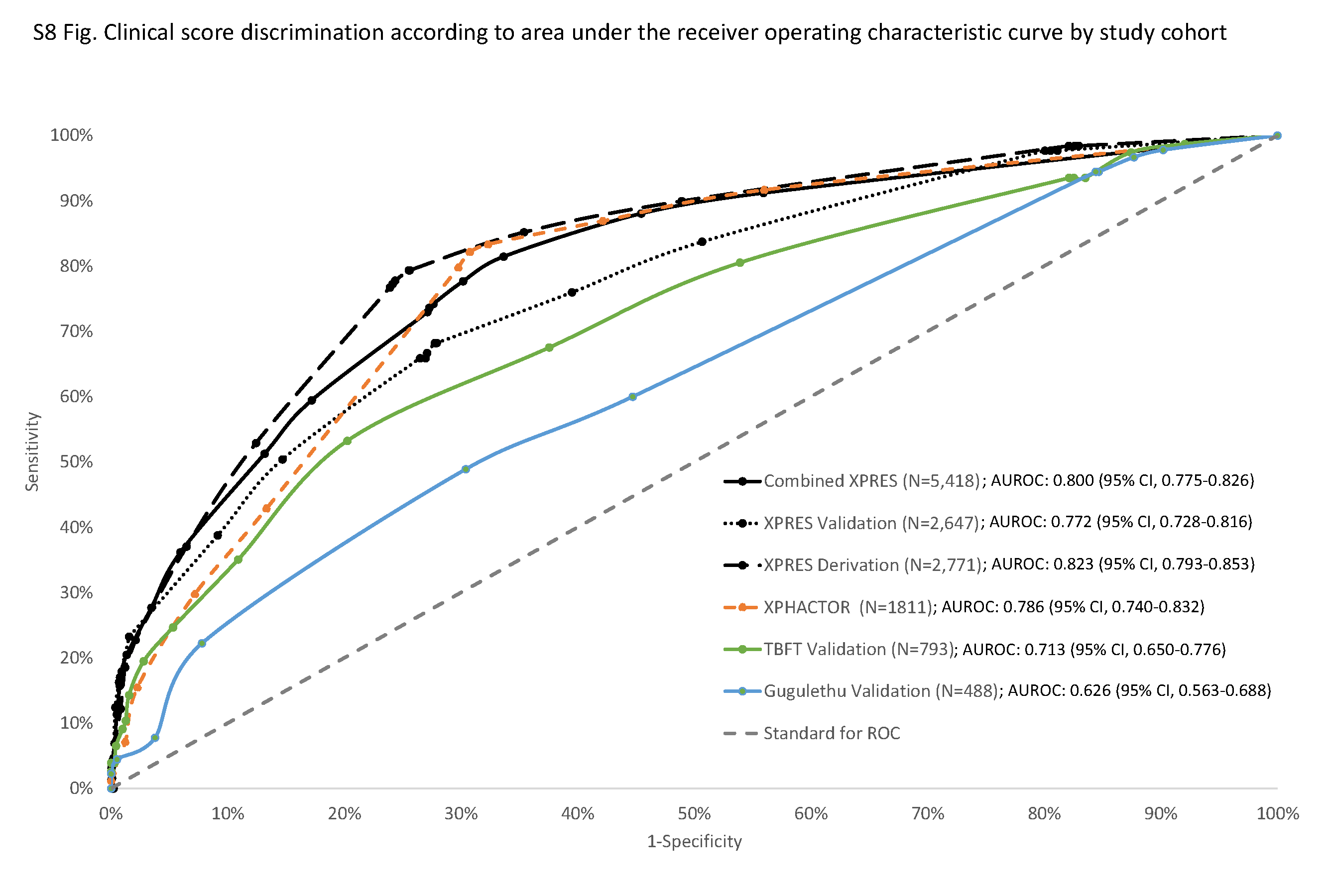

Supplement: S8 Fig — AUROC, area under the receiver operating characteristic. (TIF) [file pmed.1003739.s008.tif]
