# Supplementary material for: Derivation and external validation of a risk score for predicting HIV-associated tuberculosis to support case finding and preventive therapy scale-up: A cohort study
Source: PLoS Med. 2021 Sep 7;18(9):e1003739. doi: 10.1371/journal.pmed.1003739 (PMC8454974; doi:10.1371/journal.pmed.1003739)
Supplement: S1 Table — (PDF) [file pmed.1003739.s009.pdf]

S1 Table. HIV care clinical follow-up of clients in the Botswana XPRES cohort (2010-2015)

**Pre-ART, CD4 >350**	**3 monthly**	Weight, CD4, TB screen
**ART**	**ART start**	Weight, CD4, TB screen, ALT/AST if NVP-based regimen, Hb if AZT-based regimen, Hepatitis B screen, creatinine if TDF-based regimen
	**2 weeks**	Weight, TB screen, ALT/AST if NVP-based regimen, Hb if AZT-based regimen
	**1 month**	Weight, TB screen, ALT/AST if NVP-based regimen, Hb if AZT-based regimen
	**3 months**	Weight, TB screen, ALT/AST if NVP-based regimen, Hb if AZT-based regimen, Viral load, creatinine if TDF-based regimen
	**6months**	Weight, TB screen, ALT/AST[a] if NVP-based regimen, Hb if AZT-based regimen, Viral load, CD4
	**Quarterly[b]**	Weight, TB screen, Viral load and CD4 6 monthly, creatinine if TDF-based regimen 6 monthly

Abbreviations: CD4, CD4 cell count; TB, tuberculosis; ALT, alanine transaminase; AST, aspartate aminotransferase; NVP, nevirapine; AZT, zidovudine; TDF, tenofovir;

[a]Routine ALT/AST not required after 6 months but may be requested by the clinician depending on the clinical situation.

[b]For those patients started on PI-based regimens, baseline and 12-monthly glucose (random or fasting) and total cholesterol/triglycerides are recommended.
