# Supplementary material for: Derivation and external validation of a risk score for predicting HIV-associated tuberculosis to support case finding and preventive therapy scale-up: A cohort study
Source: PLoS Med. 2021 Sep 7;18(9):e1003739. doi: 10.1371/journal.pmed.1003739 (PMC8454974; doi:10.1371/journal.pmed.1003739)
Supplement: S2 Table — (PDF) [file pmed.1003739.s010.pdf]

S2 Table. Comparison of XPRES and External Validation Datasets

Study Characteristic	Internal Derivation and Validation Dataset (Botswana XPRES: N=5,418)	External Validation Dataset (SA, XPHACTOR: N=1,807)	External Validation Dataset (TBFT, SA: N=793)	External Validation Dataset (Gugulethu Cohort, CT, SA: N=488)
**Setting**	Botswana	Gauteng Province, SA	Gauteng, Limpopo, and North West Provinces, SA	Western Cape, SA
**Clinic Types**	5 District Hospitals and 17 Primary Health Care Clinics, purposively selected to be nationally representative	2 hospital-based and 2 community health centre (CHC) clinics	24 primary health-care clinics; only the 12 randomly selected intervention clinics included in this analysis	Community-based HIV treatment clinic in Gugulethu township
**Study Design**	Stepped-wedge trial	Prospective Cohort	Cluster Randomized Trial	Cross-sectional screening at Cohort enrollment
**Study Dates**	Enrollment: August 2012 – March 2014. Last follow-up: June 2015	Enrollment: Sept 2012–March 2014. Last follow-up: 2015.	Enrollment: December 2012 – December 2014 Last follow-up: May 2015	Enrollment: March 2010-April 2011 Last follow-up: N/A
**Eligibility Criteria for Study and analysis**	• ≥12 years old<br>• New HIV clinic enrollee, ART-naïve<br>• Not incarcerated<br>• Not already diagnosed with TB and on TB treatment<br>• Any CD4 count	• ≥18 years old<br>• Newly HIV diagnosed (HTC group), or in Pre-ART care, or on ART<br>• No TB treatment in previous 3 months<br>• Any CD4 count	• ≥18 years old<br>• No ART in the previous 6 months<br>• No TB treatment in previous 3 months<br>• CD4 count ≤150/μL<br>• No chronic liver disease, alcohol intake < 28 units/week for men, <21 units/week for women<br>• No signs/symptoms necessitating urgent referral to secondary care<br>• No intent to leave clinic catchment area within 6 months	• ≥18 years old<br>• ART-naïve<br>• No current TB diagnosis<br>• Any CD4 count
**ART eligibility**	• CD4 ≤350, stage III/IV, PBF women	• CD4 ≤200, stage IV, or CD4 ≤350 with TB or PBF women	• CD4 ≤200, stage IV, or CD4 ≤350 with TB or PBF women	• CD4 ≤200, stage IV, or CD4 ≤350 with TB or PBF women
**TB Symptom Screening**	• TB symptom screen at all visits*	• TB symptom screen at all visits*	• TB symptom screen at all visits*	• TB symptom screen at all visits*
**TB Diagnosis Ascertainment as relevant to this analysis**	• 2-4 Sputum samples collected from symptomatic enrollees<br>• Smear-microscopy and Xpert as initial test depending on phase.<br>• MGIT culture for all sputum samples<br>• Ultrasound for abdominal TB<br>• Chest x-ray per national guidelines	• ≥1 Spot sputum sample for all enrollees regardless of symptoms at enrollment<br>• Spot sputum samples collected at subsequent visits if "high risk"**<br>• Xpert for all sputum samples<br>• Chest x-ray per national guidelines	• 1 Spot sputum sample for all enrollees regardless of symptoms at enrollment in the intervention arm.<br>• Smear and MGIT culture on all enrollment samples.	• 1 Spot sputum sample, and 1 induced sputum for all enrollees regardless of symptoms at enrollment in the intervention arm.<br>• Smear, Xpert, and MGIT culture on all enrollment samples.
**TB outcome for this analysis**	• Clinical or microbiologically confirmed TB within 6 months of enrollment visit.	• Clinical or microbiologically confirmed TB within 6 months of enrollment visit.	• Microbiologically confirmed TB based on sputum sample collected at enrolment for smear and culture.	• Microbiologically confirmed TB based on sputum sample collected at enrollment and positive via Xpert, smear, or culture.
**Study-specific Primary Outcome**	• All-cause mortality<br>• TB case ascertainment a secondary outcome	• TB case ascertainment	• All-cause mortality<br>• TB case ascertainment a secondary outcome	• TB case ascertainment

Abbreviations: HTC, HIV testing and counseling; PBF, pregnant or breastfeeding women; TBFT, TB Fast Track; TB, tuberculosis; ART, antiretroviral therapy

*XPRES, XPHACTOR visits: initial visit, monthly for 3 months, then quarterly. TBFT per trial protocol. Gugulethu cohort per national guidelines.

** Spot samples were sent for Xpert MTB/RIF for (i) all assigned "high priority" (any of: current cough, fever ≥3 weeks, body mass index [BMI] <18.5 kg/m2, CD4 <100x106/l, measured weight loss ≥10% in preceding 6 months, or other feature raising high clinical suspicion of TB); (ii) those in pre-ART group with CD4<200 x106/l at enrolment and (iii) all in HTC group at enrolment, the latter two categories (who were recruited for XPHACTOR sub-studies) because of *a priori* high risk of active TB. For all other participants a spot sputum sample was frozen at -80˚C within 24 hours, for testing with Xpert at the end of the study.
