# Supplementary material for: Derivation and external validation of a risk score for predicting HIV-associated tuberculosis to support case finding and preventive therapy scale-up: A cohort study
Source: PLoS Med. 2021 Sep 7;18(9):e1003739. doi: 10.1371/journal.pmed.1003739 (PMC8454974; doi:10.1371/journal.pmed.1003739)
Supplement: S3 Table — (PDF) [file pmed.1003739.s011.pdf]

S3 Table. 15-Variable Multivariable Model in Derivation Dataset (N=2,771)

		Adjusted Odds Ratio	95% CI	p-value
Number of WHO TB symptoms (>=1)		6.25	(4.25-9.18)	0.000
Sex (Male)		1.65	(1.10-2.47)	0.015
Age in years (linear)		1.01	(1.00-1.02)	0.084
Marital Status	Married	1.00	(-)	
	Single	1.44	(0.94-2.19)	0.092
	Widowed/Divorced	1.44	(0.50-4.12)	0.498
Smoking History (ever smoked)		1.48	(1.17-1.87)	0.001
Employment Status (unemployed)		1.04	(0.69-1.57)	0.848
Prior TB (yes)		1.58	(0.86-2.91)	0.138
TB contact (yes)		1.55	(1.02-2.35)	0.042
CD4 at Enrollment		1.00	(0.997-1.00)	0.171
BMI		0.99	(0.94-1.04)	0.662
Hemoglobin at Enrollment		0.79	(0.71-0.87)	0.000
Temperature (linear term) (degrees Celsius)		1.46	(1.16-1.83)	0.001
Miner (Ever)		1.44	(0.80-2.59)	0.218
Respiratory rate (linear term)		1.02	(0.99-1.04)	0.225
Education	None	1.00	(-)	
	Primary	1.13	(0.54-2.39)	0.741
	Secondary	1.32	(0.74-2.36)	0.351
	Higher	0.62	(0.34-1.13)	0.122

Abbreviations: TB, tuberculosis; BMI, body mass index; CI, confidence interval
