# Supplementary material for: Derivation and external validation of a risk score for predicting HIV-associated tuberculosis to support case finding and preventive therapy scale-up: A cohort study
Source: PLoS Med. 2021 Sep 7;18(9):e1003739. doi: 10.1371/journal.pmed.1003739 (PMC8454974; doi:10.1371/journal.pmed.1003739)
Supplement: S4 Table — (PDF) [file pmed.1003739.s012.pdf]

S4 Table. Importance of Predictor in Logistic Regression Versus Random Forest Model

	Logistic Regression (N=2,771; 15 variables)		Random Forest Model (N=2,771; 15 variables)	
	Beta Coefficient	Rank	Mean Decrease in Gini	Rank
Temperature (first* transformed term) (degrees Celsius)	-8.028029	1	55.481	1
Number of WHO TB symptoms (>=1)	1.727382	2	20.984	7
BMI	1.616838	3	52.766	2
Smoking History (ever smoked)	0.4315334	4	5.361	12
Prior TB	0.4303915	5	5.781	10
Miner (Ever)	0.4226396	6	3.829	15
TB contact	0.4225646	7	3.931	14
Sex (Male)	0.4217461	8	5.477	11
Respiratory rate (transformed term)	-0.2790341	9	30.736	6
Hemoglobin at Enrollment	-0.2294897	10	48.020	3
Marital Status	0.2206944	11	5.357	13
Education	-0.0648862	12	13.955	8
Age in years (linear)	0.004779	13	44.020	5
Employment Status	0.0019766	14	6.564	9
CD4 at Enrollment	-0.0009955	15	46.287	4

Abbreviations: BMI, body mass index; TB, tuberculosis;
*Beta coefficient for second transformed term for temperature was 0.1157134
