# Supplementary material for: Derivation and external validation of a risk score for predicting HIV-associated tuberculosis to support case finding and preventive therapy scale-up: A cohort study
Source: PLoS Med. 2021 Sep 7;18(9):e1003739. doi: 10.1371/journal.pmed.1003739 (PMC8454974; doi:10.1371/journal.pmed.1003739)
Supplement: S5 Table — TB, tuberculosis. (PDF) [file pmed.1003739.s013.pdf]

S5 Table. Hosmer-Lemeshow test for calibration of final tuberculosis prediction model

Derivation Dataset					Validation Dataset				
			Prevalent TB					Prevalent TB	
Decile	N	Cut off*	Observed**	Predicted†	Decile	N	Cut off*	Observed**	Predicted†
1	278	0.0111	2	2.6	1	265	0.0113	3	2.5
2	277	0.0137	2	3.4	2	265	0.0138	4	3.3
3	277	0.0162	5	4.1	3	265	0.016	0	3.9
4	277	0.0191	1	4.9	4	264	0.0189	2	4.6
5	277	0.0231	4	5.8	5	265	0.0232	6	5.5
6	277	0.0299	6	7.2	6	265	0.031	10	7
7	277	0.0551	19	10.8	7	264	0.063	15	11.4
8	277	0.1099	27	23	8	265	0.1063	12	22.7
9	277	0.1881	38	39.8	9	265	0.1684	13	35.2
10	277	0.8484	85	87.4	10	264	0.8629	64	72.8
Total	2771		189	189		2647		129	169

Derivation dataset Hosmer-Lemeshow chi2(8) = 12.39, p=0.1348

Validation dataset Hosmer-Lemeshow chi2(8) = 31.39, p=0.0001

* Upper boundary of predicted risk

**Observed = observed number diagnosed with active TB within 6 months of clinic enrollment

†Predicted = expected number diagnosed with active TB within 6 months of clinic enrollment
