# Supplementary material for: Derivation and external validation of a risk score for predicting HIV-associated tuberculosis to support case finding and preventive therapy scale-up: A cohort study
Source: PLoS Med. 2021 Sep 7;18(9):e1003739. doi: 10.1371/journal.pmed.1003739 (PMC8454974; doi:10.1371/journal.pmed.1003739)
Supplement: S6 Table — TB, tuberculosis. (PDF) [file pmed.1003739.s014.pdf]

S6 Table. Performance of the TB prediction clinical score in derivation and validation datasets

	XPRES Cohort (N=5,418)			External Validation Dataset (SA, XPHACTOR: N=1811)			External Validation Dataset (TBFT, SA: N=793)			External Validation Dataset (Gugulethu Cohort, CT, SA: N=488)		
Clinical score	Total with score	Number diagnosed with prevalent TB	% Diagnosed	Total with score	Number diagnosed with prevalent TB	% Diagnosed	Total with score	Number diagnosed with prevalent TB	% Diagnosed	Total with score	Number diagnosed with prevalent TB	% Diagnosed
0	2,276	28	1%	767	7	1%	58	1	2%	41	2	5%
1	544	10	2%	243	4	2%	34	1	3%	11	1	9%
2	623	21	3%	173	3	2%	31	3	10%	14	2	14%
3	189	12	6%	28	1	4%	7	0	0%	1	0	0%
4	141	11	8%	19	2	11%	3	0	0%	0	0	0%
5	20	2	10%	0	0	0%	0	0	0%	0	0	0%
6	10	2	20%	0	0	0%	0	0	0%	0	0	0%
7	549	43	8%	315	31	10%	212	10	5%	189	31	16%
8	232	26	11%	117	11	9%	127	10	8%	67	10	15%
9	416	48	12%	97	12	12%	135	11	8%	114	24	21%
10	154	27	18%	25	7	28%	81	14	17%	29	13	45%
11	131	23	18%	23	4	17%	48	8	17%	16	3	19%
12	29	8	28%	2	0	0%	22	4	18%	4	2	50%
13	16	7	44%	1	1	100%	13	4	31%	0	0	-
14	27	14	52%	1	1	100%	5	3	60%	2	2	100%
15	24	14	58%				3	1	33%			
16	18	9	50%				6	2	33%			
17	10	8	80%				3	2	67%			
18	4	1	25%				2	-	0%			
19	5	4	80%				1	1	100%			
20							2	2	100%			
**Total**	**5,418**	**318**		**1,811**	**84**		**793**	**77**		**488**	**90**	

Abbreviations: 6m, 6 months; TB, tuberculosis; XPRES, Xpert Package Rollout Evaluation using a Stepped-wedge design trial; XPHACTOR, ªXpert for people attending HIV/AIDS care: test or review? trial; TBFT, TB Fast Track Trial; SA, South Africa; CT, Cape Town
