# Supplementary material for: Derivation and external validation of a risk score for predicting HIV-associated tuberculosis to support case finding and preventive therapy scale-up: A cohort study
Source: PLoS Med. 2021 Sep 7;18(9):e1003739. doi: 10.1371/journal.pmed.1003739 (PMC8454974; doi:10.1371/journal.pmed.1003739)
Supplement: S7 Table. Sensitivity analyses comparing discrimination between our final risk score and risk scores that (a) were derived from fully standardized bet coefficients, (b) excluded BMI, (c) excluded hemoglobin, and (d) excluded sex and smoking variables. BMI, body mass index — (PDF) [file pmed.1003739.s015.pdf]

S7 Table. Sensitivity analyses comparing discrimination between our final risk score and risk scores that (a) were derived from fully standardized beta coefficients, (b) excluded Body Mass Index (BMI), (c) excluded hemoglobin, and (d) excluded sex and smoking variables

(a) Final risk score versus risk score derived from standardized beta coefficients

	Risk Score Derived from Beta coefficients		Risk Score Derived from Standardized Beta coefficients	
	**AUROC**	**95% CI**	**AUROC**	**95% CI**
XPRES derivation (N=2,771)	0.823	(0.793-0.853)	0.822	(0.792-0.852)
XPRES validation (N=2,647)	0.772	(0.728-0.816)	0.768	(0.725-0.812)
XPHACTOR (N=1,811)	0.786	(0.740-0.832)	0.784	(0.738-0.830)
TBFT (N=793)	0.713	(0.650-0.776)	0.703	(0.641-0.765)
Gugulethu (N=488)	0.626	(0.563-0.688)	0.6194	(0.558-0.681)

(b) Final risk score versus risk score without BMI variable

	Risk Score Discrimination with BMI Included		Risk Score Discrimination Excluding BMI	
	**AUROC**	**95% CI**	**AUROC**	**95% CI**
XPRES derivation (N=2,771)	0.823	(0.793-0.853)	0.812	(0.780-0.844)
XPRES validation (N=2,647)	0.772	(0.728-0.816)	0.750	(0.704-0.795)
XPHACTOR (N=1,811)	0.786	(0.740-0.832)	0.783	(0.736-0.831)
TBFT (N=793)	0.713	(0.650-0.776)	0.702	(0.639-0.766)
Gugulethu (N=488)	0.626	(0.563-0.688)	0.602	(0.540-0.663)

(c) Final risk score versus risk score without hemoglobin variable

	Risk Score Discrimination with Hemoglobin Included		Risk Score Discrimination Excluding Hemoglobin	
	**AUROC**	**95% CI**	**AUROC**	**95% CI**
XPRES derivation (N=2,771)	0.823	(0.793-0.853)	0.818	(0.787-0.849)
XPRES validation (N=2,647)	0.772	(0.728-0.816)	0.762	(0.718-0.806)
XPHACTOR (N=1,811)	0.786	(0.740-0.832)	0.769	(0.723-0.815)
TBFT (N=793)	0.713	(0.650-0.776)	0.673	(0.608-0.738)
Gugulethu (N=488)	0.626	(0.563-0.688)	0.586	(0.525-0.647)

(d) Final risk score versus risk score without sex and smoking variables

	Risk Score Discrimination with Sex and Smoking variables included		Risk Score Discrimination Excluding Sex and Smoking Variables	
	**AUROC**	**95% CI**	**AUROC**	**95% CI**
XPRES derivation (N=2,771)	0.823	(0.793-0.853)	0.811	(0.780-0.842)
XPRES validation (N=2,647)	0.772	(0.728-0.816)	0.766	(0.722-0.809)
XPHACTOR (N=1,811)	0.786	(0.740-0.832)	0.777	(0.7312-0.822)
TBFT (N=793)	0.713	(0.650-0.776)	0.694	(0.631-0.757)
Gugulethu (N=488)	0.626	(0.563-0.688)	0.621	(0.570-0.673)
