# Supplementary material for: Derivation and external validation of a risk score for predicting HIV-associated tuberculosis to support case finding and preventive therapy scale-up: A cohort study
Source: PLoS Med. 2021 Sep 7;18(9):e1003739. doi: 10.1371/journal.pmed.1003739 (PMC8454974; doi:10.1371/journal.pmed.1003739)
Supplement: S1 Protocol — (PDF) [file pmed.1003739.s017.pdf]

S1 Protocol. Evaluating Performance, Impact, and Operational Challenges of GeneXpert Use for TB Case Finding among HIV-infected Persons In Botswana during 2012-2013:

The Xpert Package Rollout Evaluation Study (XPRES)

Version: 6.0

Date: 19 May 2017

Funding Source: The President's Emergency Plan for AIDS Relief (PEPFAR)

i. Table of Contents

i	Table of contents		2
ii	Investigators		3
iii	Abbreviations		5
iv	Summary		6
1	Introduction and Background		8
2	Objectives and Evaluation Questions		15
	2.1	General Objectives	15
	2.2	Primary Study Questions	15
	2.3	Secondary Study Questions	18
3	Methods		22
	3.1	Study Design	22
	3.2	Rationale for Study Design	28
	3.3	Study Population	29
	3.4	Randomization Procedures	33
	3.5	Sample Size	34
4	Study Procedures		41
	4.1	Site Assessment	41
	4.2	Training	41
	4.3	Prospective Study Procedures	43
	4.4	Retrospective Study Procedures	56
	4.5	Summary of Study Variables to be Collected	62
	4.6	Quality Assurance	67
5	Data Management		70
	5.1	Data Flow	70
	5.2	Data Entry	71
	5.3	Storage of Paper Questionnaires	71
	5.4	Data Transfer Procedures	72
6	Analysis Plan		72
	6.1	Study Flow Diagram	72
	6.2	Baseline Patient Characteristics	72
	6.3	Primary Study Questions	73
7	Ethical Issues		75
	7.1	Informed Consent	75
	7.2	Confidentiality	76
	7.3	Benefit for Enrolled Patients	77
	7.4	Benefit for the Program	77
8	Logistics		78
	8.1	Responsibilities	78
	8.2	Timeline	78
	8.3	Budget	80
9	References		81
10	Appendices		84
	10.1	MOH Recommended TB Diagnostic Algorithm - Adults	84
	10.2	MOH Recommended TB Diagnostic Algorithm – Children	86
	10.3	Sites Selected for XPRES	87
	10.4	Information and Consent Forms	88
	10.5	Prospective Enrollment Questionnaire and Specimen Collection Form	129
	10.6	TB Treatment Outcome Nomenclature and Drug Resistant Treatment Regimens	154
	10.7	MOH-Recommended ART Initiation Criteria and Regimens	155
	10.8	Prospective Follow-up Questionnaire	157
	10.9	Questionnaire for Patients Late for Appointments	171
	10.10	Retrospective Study Register	194
	10.11	Retrospective Data Abstraction Instrument	195
	10.12	Interview for Patients Enrolled in Retrospective Cohort	208
	10.13	Interview for Health Care Worker	217
	10.14	Chest X-ray Interpretation and Results Form	227
	10.15	Statement of Intent to Maintain Confidentiality	229

ii. Investigators

Botswana Ministry of Health:

Investigators	Position Title and Organization	Work Address & Contact Details	Role	Engaged in research?
Tebogo Madidimalo	Head of Treatment, Care and support, Department of HIV AIDS prevention and care and support Division, Ministry of health	tmadidimalo@gov.bw Tel: *+267 363 2303*	- MOH investigator<br>- Collaborates in study implementation, supervision, data analysis, and reporting of results.	Yes
Gasekgale Moalosi	Principal health Officer I, Botswana National TB Control program, Ministry of Health (MOH)	gsmoalosi@gov.bw	- MOH investigator<br>- Collaborates in study implementation, supervision, data analysis, and reporting of results.	Yes

Botswana Centers for Disease Control and Prevention:

Investigators	Position Title and Organization	Work Address & Contact Details	Role	Engaged in research?
Tefera Agizew	Senior Research Medical Officer, TB HIV division CDC Botswana	AgizewT@bw.cdc.gov, tel: +267 367 2400	- Field team supervisor<br>- Collaborates in study implementation, data analysis, and reporting of results.	Yes
Ebi Bile	Chief of Laboratory, GAP division, CDC Botswana	Bilee@bw.cdc.gov tel, +267 367 2400	- Laboratory team supervisor<br>- Collaborates in study implementation, data analysis, and reporting of results.	Yes
Joyce Basotli	Biomedical Technologist, TB/HIV division, CDC Botswana	basotlij@bw.cdc.gov tel, +267 367 2400	- Laboratory team member<br>- Collaborates in study implementation, to ensure laboratory components of the study are completed.	Yes
Balladiah Chingapane	Data manager TB/HIV Division CDC Botswana	ChingapaneB@bw.cdc.gov tel, +267 367 2400	- Data manager<br>- Supervises data entry, data checks, and data management.	Yes
Sambayawo Nyirenda	Senior Research Medical Officer, TB HIV division CDC Botswana	NyirendaS@bw.cdc.gov tel, +267 367 2400	- TB-HIV technical officer<br>- Collaborates in study implementation, supervision and advises on TB-HIV related issues.	Yes
Rosanna Boyd	Public Health Advisor, TB HIV division CDC Botswana	BoydR@bw.cdc.gov tel, +267 367 2400	- Study public health adviser<br>- Facilities study implementation, study finance management, logistics, human resources, supply procurement and distribution.	Yes
James Shepherd	Associate Director, TB HIV division CDC Botswana	Shepherd@bw.cdc.gov tel,+267 367 2400	- Study team supervisor<br>- Supervises and collaborates in study conduct, data analysis, and reporting of results.	Yes
Kathleen Toomey	Country Director, CDC Botswana	Toomeyk@bw.cdc.gov tel, +267 367 2400	- Study team supervisor<br>- Supervises and collaborates in study conduct, data analysis, and reporting of results.	Yes

Atlanta Centers for Disease Control and Prevention:

Investigators	Position Title and Organization	Work Address & Contact Details	Role	Engaged in research?
Andrew Auld	Senior Service Fellow, Medical Epidemiologist, HIV Care and Treatment Team, HIV Care and Treatment Branch, Division of Global AIDS, CDC, Atlanta	1600 Clifton Road, E-04, Atlanta Georgia, 30333 **Tel:** +1 404 639 8997 **Email:** Aauld@cdc.gov	- Lead protocol writer and CDC IRB liason<br>- Collaborates in study training, implementation, data analysis, and reporting of results.	Yes
Anand Date	Senior Service Fellow, Medical Epidemiologist, TB-HIV Team, HIV Care and Treatment Branch, Division of Global AIDS, CDC, Atlanta	1600 Clifton Road, E-04, Atlanta Georgia, 30333 **Email:** bvz7@cdc.gov	- TB-HIV Team Technical Expert<br>- Collaborates in study training, implementation, data analysis, and reporting of results.	Yes
Heather Alexander	Team Lead, International Laboratory Branch, HIV Care and Treatment Branch, Division of Global AIDS, CDC, Atlanta	1600 Clifton Road, Atlanta Georgia, 30333 **Email:** drz5@cdc.gov	- Lab Team Technical Expert<br>- Collaborates in study training, implementation, data analysis, and reporting of results.	Yes
Tedd Ellerbrock	Team Lead, HIV Care and Treatment Team, HIV Care and Treatment Branch, Division of Global AIDS, CDC, Atlanta	1600 Clifton Road, E-04, Atlanta Georgia, 30333 **Tel:** +1 404 639 8944 **Email**: Tellerbrock@cdc.gov	- Study team supervisor<br>- Supervises and collaborates in study conduct, data analysis, and reporting of results.	Yes
Bess Miller	Team Lead, TB-HIV Team, HIV Care and Treatment Branch, Division of Global AIDS, CDC, Atlanta	1600 Clifton Road, E-04, Atlanta Georgia, 30333 **Email**: bim1@cdc.gov	- Study team supervisor<br>- Supervises and collaborates in study conduct, data analysis, and reporting of results.	Yes
Sherri Pals	Statistician, Statistics Team, Epidemiology and Strategic Information Branch, Division of Global AIDS, CDC, Atlanta	**Email:** sfv3@cdc.gov	- Study team statistician<br>- Collaborates in study conduct, data analysis, and reporting of results.	Yes
Laura Broyles	Meical Officer, HIV Care and Treatment Team, HIV Care and Treatment Branch, Division of Global AIDS, CDC, Atlanta	1600 Clifton Road, E-04, Atlanta Georgia, 30333 **Email:** zjt5@cdc.gov	- HIV treatment expert<br>- Collaborates in study conduct, implementation, data analysis, and reporting of results.	Yes

Partners:

Investigators	Position Title and Organization	Work Address & Contact Details	Role	Engaged in research?
Steenhoff, Andrew P	Associate Country Director, Research Director and Lead Paediatrician; Botswana-UPenn Partnership	steenhoff@email.chop.edu; Tel: (+267 317 0933/ 721 26505)	- Pediatric HIV-TB expert<br>- Collaborates in study conduct, data analysis, and reporting of results.	Yes

iii. Abbreviations

ART	Antiretroviral therapy
ACHAP	African Comprehensive HIV/AIDS Partnerships
CDC	United States Centers for Disease Control and Prevention
CRF	Case Report Form
DSMB	Data and safety monitoring board
DST	Drug Susceptibility Testing
HIC	High-income country
HIV	Human immunodeficiency virus
IRB	Institutional review board
LED	Light-emitting diode
LIC	Low-income country
MOH	Ministry of Health
OI	Opportunistic infection
PEPFAR	United States President's Emergency Plan for AIDS Relief
PLWHA	Persons living with HIV/AIDS
PII	Personal identifying information
PY	Person-year
SOP	Standard operating procedure
TB	Tuberculosis
WHO	World Health Organization
Xpert	GeneXpert
XPRES	Xpert Rollout Evaluation Study

iv. Executive Summary

Background: In Botswana, as in the rest of sub-Saharan Africa, undiagnosed TB or TB diagnosed late in the course of disease is thought to be the most common cause of death among HIV-infected persons receiving antiretroviral therapy (ART) as well as those not yet on ART.[1-3] Failure to diagnose TB early is thought to be due to: (1) failure to appropriately screen HIV-infected patients for TB;[4] (2) difficulty in diagnosing TB with traditional diagnostic methods;[5, 6] and (3) sub-clinical TB that is missed by standard screening algorithms.[5]

Interventions for Evaluation: The recent development of the Xpert MTB/RIF assay for the GeneXpert platform (Xpert) has revolutionized TB diagnostic capability for clinicians managing HIV-infected patients. Among HIV-infected adults, TB diagnostic sensitivity of Xpert (82.4%) has been proven superior to that of smear microscopy (44.6%).[7] In line with WHO guidelines, the Botswana Ministry of Health (MOH), the United States Centers for Disease Control and Prevention (CDC), and the African Comprehensive HIV/AIDS Partnerships (ACHAP), plan to rapidly rollout the Xpert device and a new Xpert-based diagnostic algorithm while simultaneously answering important operational questions which can inform future national scale-up. In addition, to maximize impact of the Xpert device in improving detection of active TB, Xpert rollout will be preceded by strengthening of TB screening procedures by: (1) adopting the WHO-recommended 4-symptom TB screen for adults; (2) situating trained TB case-finding nurses in facilities; and (3) training health facility personnel in TB diagnostic algorithms. The combination of these strengthened TB screening procedures and rollout of the Xpert device is referred to as the "Xpert package" in this protocol.

Key Evaluation Objectives: The protocol has two key objectives: (1) to evaluate whether the new MOH-recommended Xpert-based TB diagnostic *algorithm* for new adult HIV clinic enrollees is more sensitive than the pre-Xpert smear-microscopy-based *algorithm* in diagnosing culture-positive TB disease; and (2) to evaluate the impact of the whole "Xpert package" on all-cause mortality during the first 6 months of ART, among adult patients..

Design: This is an evaluation of the phased rollout of the Xpert device using a "Step Wedge" design at 22 purposefully selected health facilities. Prior to Xpert device rollout, all 22 facilities will initiate the intensified TB screening procedures simultaneously. Subsequently, 13 Xpert devices will be initiated in a step-wise manner over nine months (one or two Xpert devices per month). The order of Xpert device rollout will be randomly assigned.

Sample Size: To answer the first primary study question with >80% power and alpha at 0.05, assuming that pre- and post-Xpert TB diagnostic algorithm sensitivities are about 62.5%[8] and 82.4%,[7] respectively, about 9,614 new adult HIV clinic enrollees will need to be enrolled (3,266 before and 6,348 after Xpert device rollout). In addition, to detect a 40% decrease in all-cause mortality during the first 6 months of ART before and after implementation of the "Xpert package" with >80% power and alpha at 0.05 assuming 6-month ART mortality rates are 15 per 100 person years[9-13] before the study starts, data will be abstracted from all ART patient enrollees (about 12,144 ART enrollees) during the 24 months prior to study start-up.

Procedures: All 22 health facilities will be assessed prior to training to troubleshoot potential logistical barriers to successful study implementation. All personnel involved in study procedures (study nurses, other health care workers involved in routine HIV care and treatment, laboratory personnel involved in diagnostic algorithms, and a data entry team) will be trained before study start and closely supervised during implementation. Patients enrolled in the prospective cohorts will be followed for 6 months after HIV clinic enrollment, or through the end of TB treatment, whichever is later. Tracing of ART patients in retrospective and prospective cohorts who are documented as late for appointments or lost to follow-up (missed last scheduled appointment by >90 days) will be performed according to national guidelines to facilitate true estimation of 6-month ART mortality.

Time line: Study enrollment is expected to start in January 2012 and be complete by July 2013, at which time analysis to answer the first primary study question will be possible. Analysis to answer the second primary study question will be possible at the end of 2013.

Budget: A budget of $ 3,051,00 is requested for all three study years 2012-2014.

1. Introduction

The majority of the world's 33.2 million HIV-infected adults and children live in resource-limited settings, where access to effective treatment with antiretroviral therapy (ART) was largely lacking prior to 2004.[14] In the absence of ART, median survival time after HIV-1-infection was 10 years for adults and two years for infants.[15] However, since 2004, due to increased political commitment and funding, especially from the United States President's Emergency Plan for AIDS Relief (PEPFAR), the global number of people receiving ART has increased, on average, by about 300,000 every six months.[16] Although outcomes to-date of ART programs in low-income countries (LIC) have been encouraging,[17, 18] early all-cause mortality among new ART patient enrollees remains high.[19]

Death from undiagnosed TB or TB diagnosed late in the course of disease is thought to be responsible for most all-cause mortality among persons receiving ART[19] as well as those not yet on ART.[20] For example, in Botswana in 2002, 40% of deaths among HIV-infected patients, who did not have access to ART or were ART-ineligible, were due to autopsy-confirmed undiagnosed TB[1] and in a large Senegalese adult ART cohort, TB accounted for at least 19% of deaths.[3] Difficulty in diagnosing TB among HIV-infected persons using traditional diagnostics (smear microscopy and chest X-ray), is thought to be responsible for the trend of missed or late TB diagnosis.[21] For example, microscopy alone, detects only about 45% of liquid culture confirmed TB cases among HIV-infected persons.[7] Even among those HIV-infected patients correctly diagnosed with TB using smear microscopy, TB disease is usually diagnosed at an advanced stage.[22-24] Adding chest X-ray for diagnosis of smear-negative TB can increase sensitivity of the TB case-finding algorithm among HIV-infected persons to about 62%,[8] but this is dependent on X-ray availability and clinician expertise, and does not solve the problem of delayed TB diagnosis.[25] Apart from morbidity and mortality, delayed TB diagnosis is also blamed for increased disease transmission.[25-27]

Multidrug-resistant (MDR) tuberculosis is also an increasing concern globally and directly threatens disease-control efforts in many countries.[28] Only 30,000 of nearly 500,000 new cases

of multidrug-resistant tuberculosis every year[28] are detected and reported,[21] and misdiagnosis causes thousands of deaths, nosocomial and community transmission, and amplification of drug resistance.[29-31]

However, the recent development of the Xpert MTB/RIF assay for the GeneXpert platform (Xpert) has revolutionized TB diagnostic capability for clinicians managing HIV-infected patients. Xpert is a PCR-based molecular test for Mycobacterium tuberculosis (MTB) and MTB with rifampicin resistance-conferring mutations. By fully integrating and automating all processes required for real-time PCR-based molecular testing, Xpert represents a simple and robust molecular test suitable for use in resource-limited settings, where TB burden is highest, and is able to provide results directly from sputum within 100 minutes. The table below summarizes key Xpert Performance characteristics among adults.[7]

Table 1: Xpert Performance Characteristics in Diagnosing Pulmonary TB among Adults (>18 years old)

	**Xpert**	**Sputum Microscopy**
**MTB Diagnosis among HIV-infected Adults**		
Sensitivity - overall	82.4% (76.7–86.9%)	44.6% (37.7–51.6%)
Sensitivity – among smear-positive	97.7% (91.9–99.4%)	100%
Sensitivity – smear-negative	71.8%, (63.3–78.9%)	0%
Specificity	99.2%, (97.8–99.7%)	100·0% (99.4–100.0%)
Positive Predictive Value	Estimated at 96.3%*	100.0%*
Negative Predictive Value	Estimated at 95.8%*	12.2%*
**MTB Diagnosis among HIV-negative Adults**		
Sensitivity - overall	90·7% (87·2–93·4%)	68·6% (63·5–73·3%)
Sensitivity – among smear-positive	99·0% (96·5–99·7%)	100%
Sensitivity – smear-negative	77·5% (69·6–83·9%)	0%
Specificity	99·3% (98·5–99·7%)	99·4% (98·8–99·7%)
Positive Predictive Value	Estimated at 97.0%*	Estimated at 96.6%*
Negative Predictive Value	Estimated at 97.7%*	Estimated at 92.7%*
**MTB/RIF Diagnosis (data only available for both HIV+ve & -ve adults)**		
Sensitivity - overall	94.4%, (90.8–96.6%)	N/A
Specificity	98.3% (97.1–99.0%)	N/A
Positive Predictive Value**	94.4%	N/A
Negative Predictive Value**	98.3%	N/A

*Assumes 20% TB prevalence
**Prevalence of Rifampicin (RIF) resistance was 23.6% (250/1060). As prevalence of RIF resistance decreases, so does positive predictive value (PPV). It is estimated that in Botswana,

where MDR TB prevalence is about 2.5% and RIF resistance about 5%, Xpert PPV for RIF-resistant MTB will be about 71%.[32]

Xpert performance characteristics for HIV-infected and HIV-negative persons are similar, however, for HIV-infected persons, superiority of Xpert TB diagnostic sensitivity over smear microscopy is more notable.[7] Because smear microscopy for HIV-negative persons remains a reasonably sensitive TB diagnostic test, and because the current cost of Xpert set-up, and training is about $80,000-100,000 per year,[32] WHO currently recommends the following:

(1) Xpert MTB/RIF should be used as the initial diagnostic test in individuals suspected of having MDR-TB or HIV-associated TB. (Strong recommendation).
(2) Xpert MTB/RIF may be considered as a follow-on test to microscopy in settings where MDR-TB or HIV is of lesser concern, especially in further testing of smear-negative specimens. (Conditional recommendation acknowledging major resource implications).

In Botswana, which has a high HIV prevalence (about 25% among adults aged 15-49),[14] high annual TB case notification rate (about 550/100,000)[33], high TB-HIV co-infection rate (about 80% of TB clinic patients are also HIV-infected according to routine MOH program monitoring data), and an increasing prevalence of MDR TB (from 0.2% in 1996 to 2.5% in 2008),[33] Xpert rollout for use in HIV care and treatment centers is an urgent priority. As part of the national strategy, the Botswana Ministry of Health (MOH) plans to rollout Xpert along with a package of complementary TB case finding interventions. The combination of Xpert and complementary TB case finding interventions will be referred to as the "Xpert package" in this protocol.

The complementary TB case finding activities include:

(1) Adoption of the WHO-recommended 4-symptom TB screen (cough of any duration, fever, weight loss, and night sweats) for HIV-infected adults (>12 years old);[34]
(2) Introduction of clinic TB case finders, responsible for daily TB screening, sample collection, sample transport to the laboratory, and reporting of results to diagnosing clinicians; and,
(3) Training for health care workers in correct use of recommended case finding algorithms for adults (>12 years) and children (≤12 years).

This protocol is designed primarily to:

(1) Evaluate the added advantage of Xpert-based pulmonary TB diagnostic algorithms over smear microscopy-based algorithms for HIV-infected adult TB suspects, when the complementary TB case finding interventions (the new 4-symptom TB screen, TB case-finders, and training of clinic staff in intensified case finding algorithms) are in place.

Rationale: WHO recommends evaluation of Xpert diagnostic performance during rollout efforts [32]. Although Xpert has been proven more sensitive than smear microscopy for TB diagnosis in TB suspects, superiority of the Xpert-based algorithm for adults over the smear microscopy based algorithm, which includes chest X-ray for smear negative TB suspects, has not yet been demonstrated.

(2) Evaluate the impact of the Xpert package on 6-month adult ART patient all-cause mortality rates.

Rationale: This is a WHO-recommended Xpert evaluation question [32]. TB may go undiagnosed at ART initiation for several reasons, of which the three most common are thought to be: (a) failure to adequately follow TB-screening guidelines;[4] (b) subclinical disease (asymptomatic disease) in patients who were correctly screened for TB;[5] and (c) failure to detect active TB infection among TB suspects due to insensitive diagnostic tests (usually, sputum microscopy and chest X-ray):[5, 7]

(a) <u>Failure to correctly screen ART patients for TB</u>: In a systematic review of clinic records of adults receiving ART in Mozambique during 2004-7, only 57% of records had documentation of any TB symptom screening, while only 5% of records documented the full 4-symptom screen.[4] Reasons for non-compliance with TB screening guidelines are unknown, but likely relate to human resource constraints and high workload of attending health care workers. By situating one TB case finding nurse in each of the 22 facilities, this problem of failure to comply with TB screening guidelines will hopefully be overcome.

(b) <u>Subclinical TB</u>: Active TB symptoms are due to both the bacillary burden and the host's immune response. Therefore, highly immune suppressed patients enrolled in ART, who have active TB, may not have TB symptoms and therefore may not screen positive or be tested for active TB. For these patients, ART initiation and subsequent restoration of the immune system can lead to rapid development of severe TB symptoms and florid

disease.[5] The Xpert package will not reduce the incidence of "unmasked TB" during the first 6 months of ART. Therefore, this will not be a possible source of all-cause mortality reduction.Insensitive diagnostic tests: Failure to correctly diagnose smear-negative TB cases among identified TB suspects is another possible reason for missing active TB among ART enrollees. Progression of undiagnosed TB during the first six months of ART is thought to be an important cause of mortality and morbidity.[5] If the Xpert package is correctly implemented, with earlier diagnosis of active TB disease prior to ART start, this should reduce the prevalence of undiagnosed TB during the first 6 months of ART. This in turn might reduce all-cause mortality, by reducing TB-specific mortality.

We will design the evaluation so that we can assess all-cause mortality rates during the first 6-months of ART (1) before study initiation through retrospective evaluation of ART medical records belonging to ART patients enrolled in the 24 months prior to study initiation at each clinic (this retrospective cohort is referred to as cohort "R" in this protocol), (2) after rollout of the complementary TB case finding interventions among prospectively enrolled patients before Xpert device rollout (this cohort is referred to as cohort "A" in this protocol), and (3) after subsequent rollout of the Xpert device in addition to existing complementary TB case finding interventions in the prospective post-Xpert cohort (referred to as cohort "B" in this protocol). However, for the purpose of sample size calculations, and simplicity, our primary question will be: "Are incidence rates of all-cause mortality in the first 6 months of ART among adult patients, significantly different between cohorts "R" and "B"?"

Some important secondary questions, which the evaluation will assess, include:

(1) Assessing whether the performance characteristics of the Xpert device varies according to whether the Xpert device is placed in a laboratory and operated by technicians with previous laboratory training or whether it is located in a point-of-care facility and operated by personnel without previous formal laboratory training.
Rationale: Most studies evaluating Xpert performance have been located in national reference centers.[7] However, the Xpert MTB/RIF assay was designed specifically for use

close to point-of-treatment in endemic disease settings. Possible factors that may impact Xpert performance at peripheral centers include the need for: a stable electricity supply, an operating temperature <30 degrees Celsius , and storing cartridges at 2-28 degrees Celsius prior to use. One recent multi-country study assessed performance of Xpert in sub-district TB microscopy labs, but the study did not include Botswana, and did not include evaluation of Xpert use by non-laboratory personnel.[7] This question is important in the context of Botswana's TB-HIV program because it may inform location of future Xpert machines.

(2) Assessing the best use of Xpert for diagnosis and management of drug resistant TB. Specifically, the protocol will assess:

(a) What proportion of Xpert-diagnosed TB patients, assessed as Rifampicin (RIF) sensitive by Xpert, have TB resistant to the other first-line TB drugs [Isoniazid (INH), Ethambutol and Pyrazinamide)?

Rationale: An Xpert sputum result showing lack of resistance to RIF, does not exclude resistance to other TB drugs. By exploring the prevalence of non-RIF TB drug resistance in Botswana, this study could help to inform future algorithms for diagnosis and management of patients at high risk for TB drug resistance.

(b) What is the sensitivity, specificity, PPV, and NPV of Xpert-diagnosed rifampicin-resistant TB as a marker of: (1) mono RIF resistance; (2) MDR TB (RIF+INH resistance).

Rationale: Table 2 presents the PPV and NPV for RIF resistance using Xpert MTB/RIF in settings or populations with varying prevalence of RIF resistance. Due to the high sensitivity of the assay, the NPV is over 99% in settings with both low and high prevalence of MDR-TB resistance, i.e. a negative result accurately excludes the possibility for RIF resistance.

However, in low MDR-TB prevalence settings, the PPV of Xpert MTB/RIF testing is adversely affected; for example, in Botswana, where the prevalence of RIF resistance is thought to be 2.5-5% among all new TB cases, PPV of MTB/RIF testing is thought to be about 60-71%.[32]

Table 2: False positive, false negative, and predictive values for RIF resistance using Xpert MTB/RIF, according to varying prevalence of RIF resistance in a population of 1000 persons

% Prevalence of RIF resistance	True Positives	False Positives	False Negative	True Negative	PPV	NPV
1%	9.5	19.8	0.5	970.2	32.4%	99.9%
2%	19	19.6	1	960.4	49.2%	99.9%
**3% ***	**28.5**	**19.4**	**1.5**	**950.6**	**59.5%**	**99.8%**
**4% ***	**38**	**19.2**	**2**	**940.8**	**66.4%**	**99.8%**
**5% ***	**47.5**	**19**	**2.5**	**931**	**71.4%**	**99.8%**
6%	57	18.8	3	921.2	75.2%	99.7%
7%	66.5	18.6	3.5	911.4	78.1%	99.6%
8%	76	18.4	4	901.6	80.5%	99.6%
9%	85.5	18.2	4.5	891.8	82.4%	99.5%
10%	95	18	5	882	84.1%	99.4%
11%	104.5	17.8	5.5	872.2	85.4%	99.4%
12%	114	17.6	6	862.4	86.6%	99.3%
13%	123.5	17.4	6.5	852.6	87.7%	99.2%
14%	133	17.2	7	842.8	88.5%	99.2%
15%	142.5	17	7.5	833	89.3%	99.1%

Source: for the above table: WHO. Rapid Implementation of the Xpert MTB/RIF diagnostic test.[32]

The findings of this study may have implications for future management of TB patients diagnosed as RIF-resistant, especially in settings where culture and drug susceptibility testing (DST) results are not rapidly available.

(c) How does the presence or absence of risk factors for TB drug resistance (e.g. previous TB treatment) influence the PPV of Xpert MTB/RIF assay for RIF-resistance or MDR TB?

Rationale: Previously treated TB cases (among whom MDR TB prevalence is estimated at 15.3%),[32] who test positive for RIF-resistance should have a higher PPV for true RIF-resistance and MDR TB. These results may inform future guidelines on who needs to have resistance confirmed with liquid culture and DST before MDR treatment initiation, and who can be assumed to have RIF-resistance/MDR TB without further DST results.

Other important Xpert-associated operational issues that will be assessed with this protocol include assessment of Xpert-based algorithms for HIV-infected children (≤12 years of age), laboratory-specific turnaround times such as time from sputum collection to result return to the

patient. Cost and cost-effectiveness issues will be evaluated, but their evaluation is described in a separate protocol.

Further Study Rationale and Justification:

Published WHO support for the primary study questions of this protocol can be found at: http://whqlibdoc.who.int/publications/2011/9789241501569_eng.pdf, in the WHO document titled "*Rapid Implementation of the Xpert MTB/RIF diagnostic test*". In this WHO document, a panel of WHO experts (who are listed on page 3) supports evaluation of:

(1) The diagnostic performance of the GeneXpert apparatus in routine program settings (Page 19, section 9.1)..

(2) The impact of GeneXpert on patient treatment outcomes (Page 21, section 9.2, #5.)..

Operational research is important to inform planned future rollout in Botswana and other resource-limited settings. For example, poor performance (low sensitivity) of the planned Xpert-based diagnostic algorithm compared with the current microscopy-based algorithm would be important to detect and, if possible, correct, before nation-wide rollout of the Xpert devices, in this resource-limited setting. Alternately, if the study demonstrates improved diagnostic sensitivity of the new Xpert-based algorithm, as well as a significant impact of GeneXpert on patient outcomes (e.g. mortality) this should accelerate willingness of Botswana and other resource-limited countries to invest in purchase of Xpert devices for the benefit of their HIV-infected patients. This could lead to reductions in morbidity and mortality, both in Botswana and elsewhere.

The study design we have chosen to evaluate both the diagnostic performance of the test and the impact of the test on patient outcomes, is the stepped-wedge design. When WHO released guidance on possible study designs to use when evaluating the rollout of the Xpert device, WHO recommended use of this study design. Please see page 32 of the WHO publication, under the section "*Annex 4. Piloting implementation of Xpert MTB/RIF: potential operational research study designs*".

It is the goal of CDC Botswana and the Botswana MOH to improve the efficacy of patient care while remaining cost-effective. This study will demonstrate whether the CDC-MOH partnership has improved the efficacy of patient care, by showing whether there has been an improvement in TB diagnostic sensitivity of the new Xpert-based algorithm, and showing whether there is a detectable improvement in patient outcomes. A separate protocol is aimed at assessing the costs of Xpert rollout and will inform the cost-effectiveness analysis.

Brief Description of ART Eligibility Criteria in Botswana:

**Patient**	**Eligibility Criteria**
**Adults**	
**Pregnant**	Life-long ART if:<br>- CD4<350<br>- WHO stage III/IV<br>Otherwise, ART for pregnancy and throughout breastfeeding.
**Non-pregnant**	Life-long ART if:<br>- CD4<350<br>- WHO stage III/IV
**Children**	
**≤12 months old**	All infants are eligible for ART
**>12 months**	- WHO clinical stage 3 or 4<br>- Advanced or severe immune suppression per WHO age-related guidelines (see table 13 of protocol)

2. Objectives and Evaluation Questions

2.1. General Objectives

The specific study questions can be grouped under the following general objectives:

(a) to evaluate the improvement in (i) TB case finding, and (ii) drug resistant TB case finding, achieved by the Xpert package;

(b) to evaluate the impact of the Xpert package on HIV-infected patient outcomes;

(c) to assess best practices for rollout of the Xpert machine to inform future scale-up; and,

(d) to investigate Botswana-specific laboratory issues about Xpert machine use.

2.2. Primary Study Questions

The two primary study questions fall within the categories of the first two general objectives:

(a) **Intensified TB case finding:**

Among HIV-infected adults* presenting for care at HIV care and treatment centers in Botswana, who screen positive for TB using the WHO-recommended four-symptom screen, is the Xpert-based TB diagnostic algorithm more sensitive than the pre-Xpert smear-microscopy-based TB diagnostic algorithm in diagnosing culture-positive TB disease?

*Note that in this protocol, “adult” refers to an individual >12 years old and “child” refers to an individual ≤12 years old; this is because the TB diagnostic algorithms are different for persons aged >12 years versus ≤12 years. For consent-related issues, the definitions of “adult” and “child” change and will be described separately. The pre-Xpert and Xpert-based diagnostic algorithms for HIV-infected adult TB suspects who present to HIV care and treatment clinics is illustrated below, as well as the planned numerator and denominators of the sensitivity proportions to be compared.

Figure 1: Comparison of Pre-Xpert and Xpert-based TB Diagnostic Algorithms in Adults

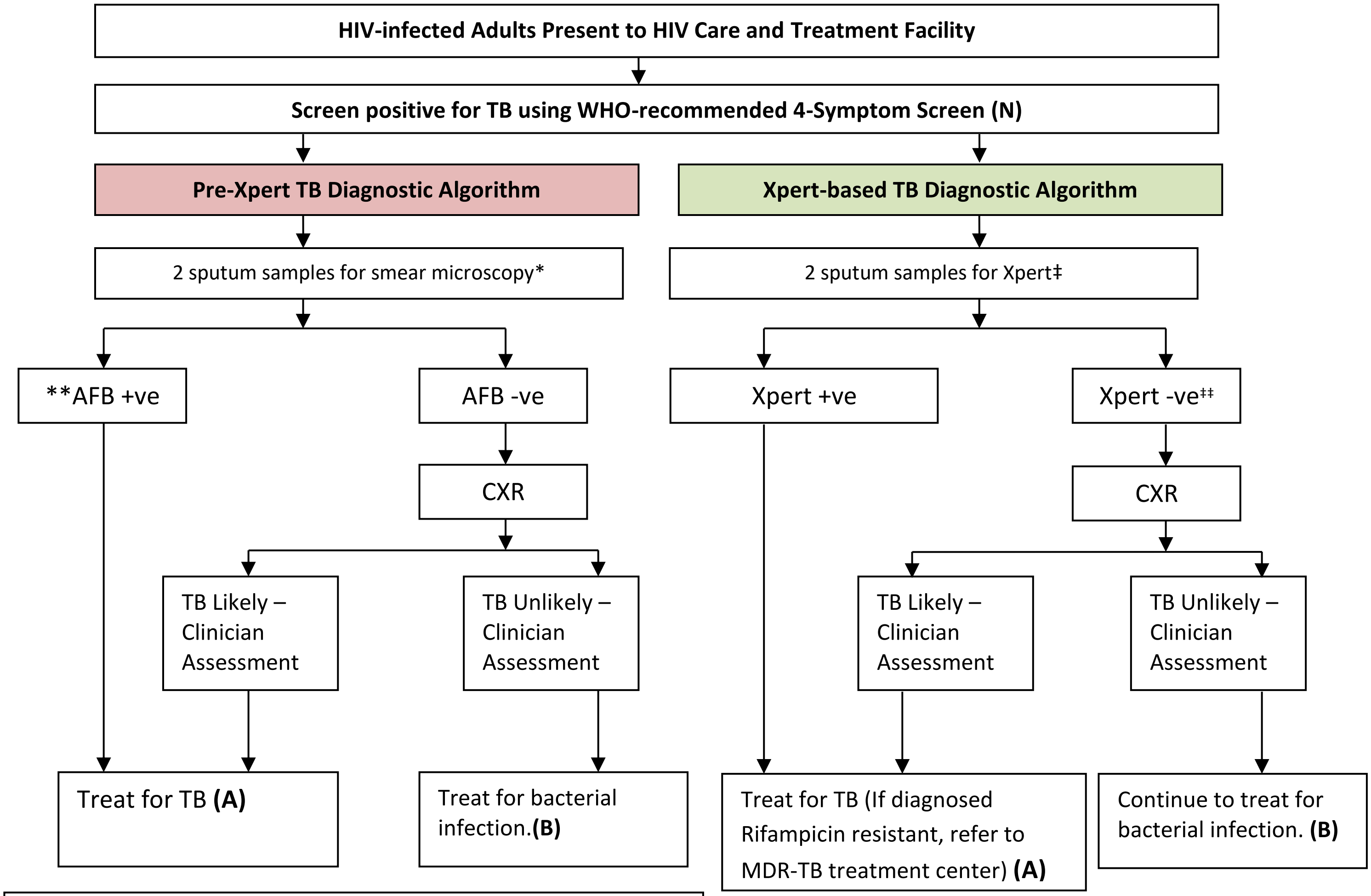

*In the study, two sputa are also collected for liquid TB culture at this stage. In the Botswana national guidelines 1 sputum is supposed to be sent for liquid culture for all HIV-infected TB suspects at this stage, however, this is not routinely done.
**AFB = Acid Fast Bacilli. AFB +ve means AFB's were identified through smear microscopy using a standard Ziehl-Neelsen stain. AFB –ve means AFB's were not identified.

‡In the study, two sputa are also collected for liquid TB culture at this stage.

Sensitivity Proportions of the Pre- and Post-Xpert TB Diagnostic Algorithms

		Liquid TB Culture Results		
		**TB +ve**	**TB -ve**	
**Diagnostic algorithm**	**TB +ve**	**(A – FP)**	**FP**	**A**
	**TB -ve**	**FN**	**(B-FN)**	**B**
		**(A – FP)+FN**	**FP+(B-FN)**	**Total (N)**

FP=false positive; FN=false negative

Sensitivity = $\mathbf{(A - FP)} / \mathbf{(A - FP) + FN}$

Note that for HIV-infected adult patients with danger signs (temperature >39 degrees centigrade, respiratory rate >30/min, pulse >120/min, or unable to walk unaided), the pre-Xpert diagnostic algorithm is slightly different (e.g. involves immediate hospital admission, treatment with parental antibiotics, and treatment for TB after 3-5 days of non-response to antibiotics regardless of X-ray findings among smear negative patients) (Appendix 11). Most patients (>95%) presenting to the HIV care and treatment clinics will not have danger signs, however, those patients with danger signs will still be eligible for enrollment. During analysis, the sensitivity of the pre-Xpert TB diagnostic algorithm will be considered including and excluding patients considered to have danger signs.

Note also that the screening algorithms – both the pre-Xpert and Xpert – are to be implemented at the facility level, and that all patients being seen at participating facilities will be screened using the implemented algorithms, regardless of study eligibility or participation.

(b) The impact of the Xpert package on all-Cause ART Patient Mortality

Are pre- versus post-Xpert package incidence rates of all-cause mortality in the first 6 months of ART among adult patientssignificantly different? (Note this evaluation question compares all-cause mortality rates during the first 6 months of ART between ART enrollees in cohorts R and B) (Note that only patients who are ART-eligible and start ART during the enrolment periods of interest will be included in this analysis).

2.3. Secondary Study Questions

(a) Intensified TB Case Finding:

Comparing adult patients enrolled on ART before and after implementation of the *complementary TB case finding interventions* (Use of new 4-symptom TB screen, TB nurses, and additional training in TB case finding):

(1) What proportion of new adult ART patients are documented to have been screened for active TB using the recommended algorithm?

(2) What proportion of new adult ART patients screen positive for TB?

(3) What proportion of new adult ART enrollees are diagnosed as having TB?

(4) Comparing children (≤12 years) enrolled in HIV care and treatment before Xpert package rollout and after implementation of the Xpert package, is the Xpert-based TB diagnostic algorithm more sensitive than the pre-Xpert smear-microscopy-based TB diagnostic algorithm in diagnosing culture-positive TB disease? (See figure 2 below and appendix 2 for the full pre-Xpert algorithm for children)

(5) Among children at the first HIV clinic enrollment visit, how sensitive is the MOH-recommended 6-symptom TB screen in identifying HIV-infected children with culture-positive TB?

Figure 2: TB Diagnostic Algorithm for Child TB Suspects

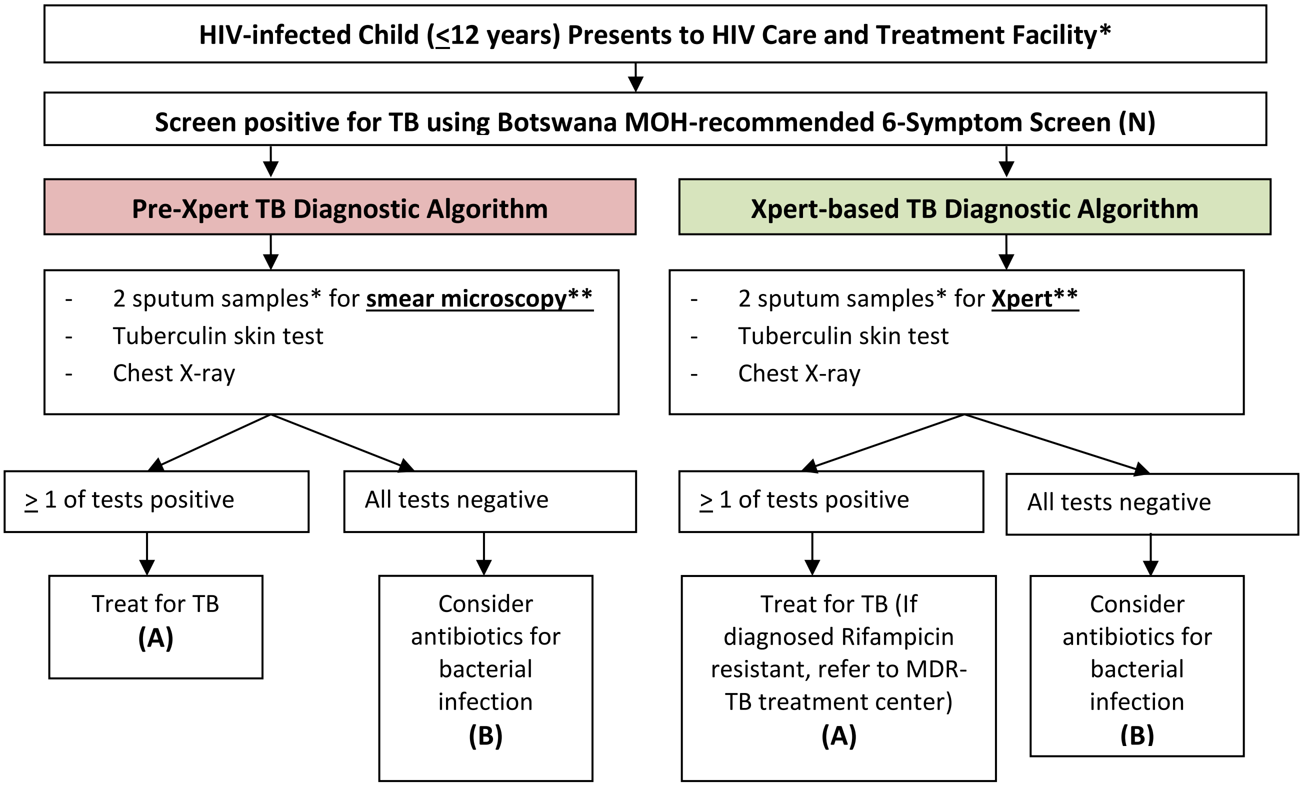

*Sputum may be obtained using sputum induction or gastric aspirates if necessary (described in detail in section 4.3 "Prospective Study Procedures")
**In the study, two sputa are also collected for liquid TB culture at this stage. In the Botswana national guidelines the same 2 sputa examined with smear microscopy or Xpert are supposed to be sent for liquid culture for all HIV-infected TB suspects at this stage, however, this is not routinely done due to resource constraints

Sensitivity Proportions of Pre- and Post-Xpert TB Diagnostic Algorithms

		Liquid TB Culture Results		
		**TB +ve**	**TB -ve**	
**Diagnostic algorithm**	**TB +ve**	**(A – FP)**	**FP**	**A**
	**TB -ve**	**FN**	**(B-FN)**	**B**
		**(A – FP)+FN**	**FP+(B-FN)**	**Total (N)**

FP=false positive; FN=false negative. (FN and FP diagnoses are identified by liquid culture results.)

$$\textbf{Sensitivity} = (\mathbf{A} - \mathbf{FP}) \big/ (\mathbf{A} - \mathbf{FP}) + \mathbf{FN}$$

Note: MOH-recommended 6-symptom TB screen for children (≤12 years old) is: (1) Weight loss or failure to thrive (no weight gain over 3 months); (2) Enlarged lymph nodes (more than 1 x 1 cm); (3) Cough for ≥ 2 weeks; (4) Fever for ≥ 2 weeks; (5) Fatigue/reduced playfulness ≥ 2 weeks; (6) Profuse night sweats ≥ 2 weeks.[33]

Note also that the screening algorithms – both the pre-Xpert and Xpert – are to be implemented at the facility level, and that all patients being seen at participating facilities will be screened using the implemented algorithms, regardless of study eligibility or participation.

*At the prospective enrollment visit, for HIV-infected children, who screen negative for TB using the 6-symptom MOH screen, we will collect 2 sputa for TB culture to evaluate the sensitivity of the MOH symptom screening algorithm. The first sputum or gastric aspirate will be collected on day 1 and the second sputum or gastric aspirate will be collected on the morning of day 2. After the enrollment visit, only symptomatic children will submit sputum for the diagnostic processes described in figure 2.

(6) Among all patients enrolled in HIV care and treatment centers after rollout of the Xpert package, what is the increase in sensitivity achieved by performing chest X-ray on Xpert negative TB suspects?

Intensified Drug Resistant TB Case Finding:

(7) *After* implementing the complementary TB case finding interventions, what is the increased sensitivity of the Xpert-based TB diagnostic algorithm over the pre-Xpert TB diagnostic algorithm in identifying drug resistant TB?

[Note: We anticipate that the Xpert-based algorithm will correctly identify more patients needing liquid culture and DST for identification of drug resistant TB for two reasons: (1) we anticipate that the Xpert-based algorithm will correctly identify more TB cases among TB suspects; and (2) in the pre-Xpert TB diagnostic algorithm, sputa for liquid culture and drug susceptibility testing (DST) are usually only requested for TB patients with risk factors for drug resistant TB (retreatment failure, new treatment failure, patients beginning retreatment, history of inappropriate treatment, contacts of known MDR and XDR-TB patients, residents or workers in health facilities prisons or TB laboratories, patients who develop TB disease while taking isoniazid preventive therapy (IPT)); some MDR TB patients, who screen negative for TB drug resistance risk factors, may be diagnosed as RIF-resistant by Xpert.]

(8) Among patients enrolled in the study after Xpert package rollout, how accurately does the Xpert diagnosis of rifampicin resistance reflect existence of MDR TB (rifampicin and isoniazid resistance) as determined by the gold standard measure of liquid TB culture and DST?

(9) By how much does the existence of TB drug resistance risk factors at HIV clinic enrollment among TB suspects, who test Xpert MTB/RIF positive, increase PPV of Xpert-diagnosed RIF-resistance in predicting (1) true RIF-resistance; (2) true MDR TB?

(b) HIV Patient Outcomes:

Before study start-up and after implementation of the full Xpert package:

(1) Is there a difference in TB incidence during the first 6 months of ART among adults?

(2) Is there a difference in adult TB treatment outcomes among new HIV clinic enrollees, who are diagnosed with TB in the first 6 months after HIV clinic enrollment?

(3) What are the incidence rates of hospitalization during the first 6 months of adult ART?

(c) Best practices for Xpert package scale-up nationally

(1) Does Xpert device (GeneXpert) performance vary by its location? Specifically, is there a difference in Xpert device TB diagnosis sensitivity depending on whether the Xpert device is located at laboratories and operated by trained laboratory technicians or located

at point-of-care (POC) facilities and operated by personnel without formal laboratory training?

(2) Of all Xpert batches attempted, for what proportion of attempts was Xpert considered inoperable for any duration of time?

(3) Over all occurrences of the Xpert device being inoperable, what was the median duration in hours that Xpert was considered inoperable?

(4) What were the reasons that Xpert could not be operated?

(5) Did frequencies of reasons for Xpert being inoperable differ by location of the Xpert test?

(6) What is the mean and median monthly number of days Xpert is unable to operate?

(d) Laboratory Issues Surrounding Use and Performance

After implementation of the Xpert package:

(1) What is the mean and median time from screening positive for TB to specimen collection?

(2) What is the mean and median time from sputum/gastric aspirate collection to: (i) specimen receipt at the testing site; (ii) result availability at the laboratory; (iii) result return to the patient folder; (iv) TB diagnosis (clinician decision); (v) result return to the patient; and (vi) initiation of TB treatment?

(3) Will the performance (sensitivity, specificity, positive predictive value, negative predictive value) of Xpert in diagnosing TB, change over the course of the study?

(4) What proportion of newly diagnosed TB patients are lost to follow-up before they can receive the TB diagnosis and start TB treatment?

3. Methods

3.1. Study Design

This is an evaluation of the phased rollout of the Xpert package using a "Step Wedge" design.[35, 36] Figure 3 is a diagrammatic representation of the proposed design.

Figure 3: Phased Rollout of the Xpert Package

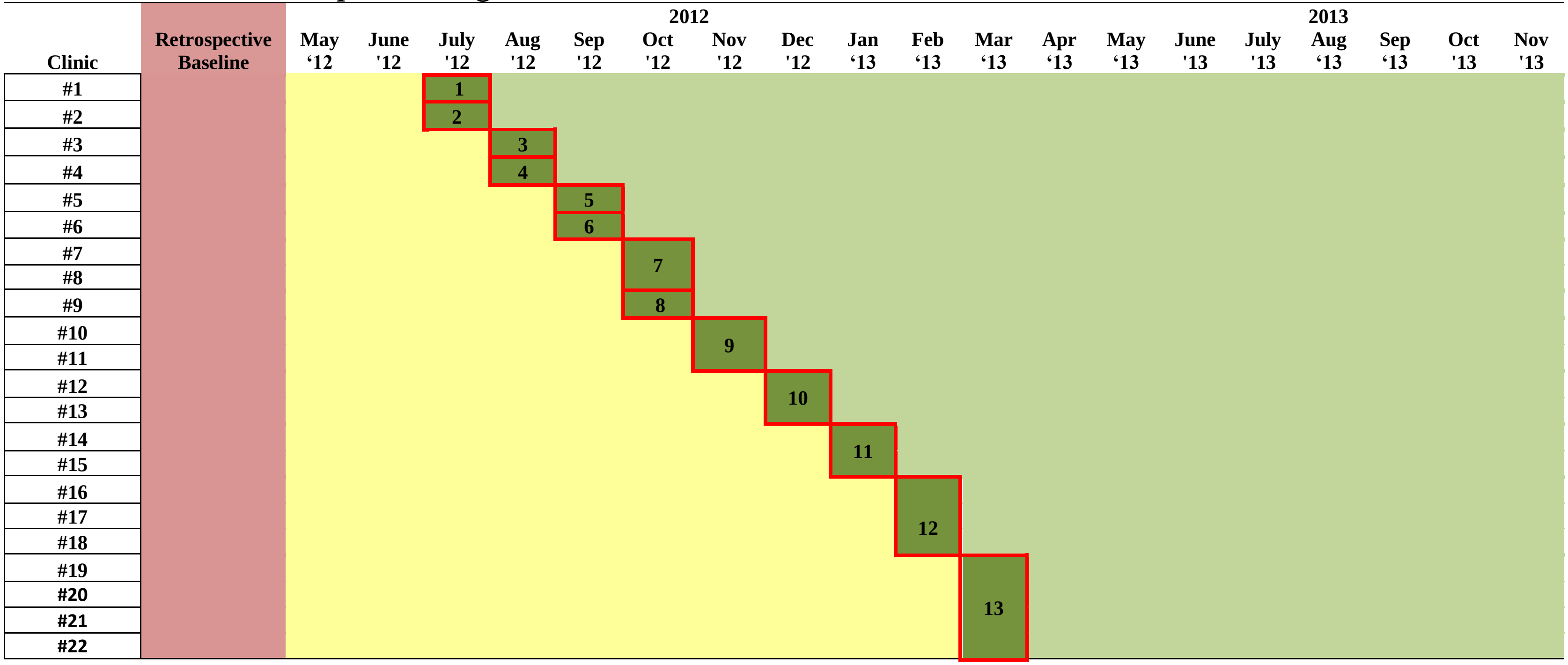

Key	Retro- or Prospective Enrollment?	Complementary TB Case Finding Intervention in place?	Xpert device in place?
(pink)	Retrospective	No	No
(yellow)	Prospective	Yes	No
(green)	Prospective	Yes	Yes

(green box with red border) Indicates initiation of one Xpert device. There are 13 Xpert devices.

Complementary TB Case Finding Intervention:

(1) Adoption of the WHO-recommended 4-symptom screen (cough of any duration, fever, weight loss, and night sweats) for TB screening among HIV-infected adults (>12 years old);
(2) Introduction of clinic TB case finders, responsible for daily TB screening, sample collection, sample transport to the laboratory, and reporting of results to diagnosing clinicians; and,
(3) Training for health care workers in correct use of recommended case finding algorithms for adults (>12 years) and children (<12 years).

Explanation of diagram:

- **Clinics:** 22 HIV care and treatment centers will be purposefully selected for Xpert rollout and inclusion in the evaluation. (See clinic selection criteria in section 3.3)

- **Prospective Baseline Phase (shaded yellow):** During this phase, HIV-infected patients will be enrolled into routine HIV care and treatment (HIV C&T) at clinics where the *complementary TB case finding interventions* (which include the new WHO-recommended 4-symptom screen, TB case finding nurses, and training for health care workers in completion of the TB diagnostic algorithms) will be in place. All consenting patients *newly* presenting to the 22 clinics will be enrolled in the evaluation to answer the primary study question which compares sensitivity of the pre-Xpert smear microscopy-based TB diagnostic algorithm with the Xpert-based TB diagnostic algorithm. This population will be referred to as "study population A" in the rest of the protocol. (Patient population inclusion and exclusion criteria are further described in section 3.3.)

- **Prospective Phase (shaded green):** During this phase, HIV-infected patients will be enrolled into routine HIV care and treatment at clinics where the Xpert package will be in place. All consenting patients *newly* presenting to the 22 clinics during this phase will be enrolled in the evaluation to help answer both primary study questions. This population will be referred to as "study population B" in the rest of the protocol. The rationale for the duration of enrollment into this prospective post-Xpert cohort phase is described in the sample size requirements section.

- **Retrospective Baseline Phase (shaded red):** At the 22 clinics during this pre-study phase, HIV-infected patients were enrolled into routine HIV care and treatment and should have received the standard pre-Xpert TB screening and diagnostic algorithm at HIV care entry (appendix 1), although the extent to which clinicians adhered to national guidelines during this time period is unknown. During this phase, permanent MOH-recommended medical records would have been opened for all ART patients but not for all ART-ineligible patients. To answer the second primary question, we will select all patients initiating ART at the 22 selected clinics in the 24 months prior to study start-up

to estimate all-cause mortality rates during the first 6 months of ART and compare this all-cause mortality with that observed during full Xpert package implementation. The size of the pre-Xpert retrospective cohort will be defined by sample size estimations needed to answer this second primary study question with the required degree of power. This population will be referred to as "study population R" in the rest of the protocol.

- **Follow-up duration for prospectively enrolled patients:** Prospectively enrolled patients will be followed up for 6 months after enrollment or until the end of TB treatment. The rationale for the minimum 12 month follow-up period is to facilitate answering one of the secondary questions (question B.2.), which investigates possible differences in pre- versus post-Xpert package TB treatment outcomes among new HIV clinic enrollees, who are diagnosed with TB in the first 6 months after HIV clinic enrollment. Although most incident TB during *ART* occurs in the first six months of therapy, some incident TB does occur in months 6-12. For example, in a study by van Rie in South Africa, of the 430 TB cases occurring during the first ART year, 146 (34%) occurred during months 6-12.[13] Among patients who are initially ineligible for ART, it is unclear whether TB incidence will decrease (due to early screening and diagnosis) or increase (as CD4 counts continue to fall). By capturing all incident TB cases in the first 6 months post enrollment, precision of post-Xpert package TB treatment outcome estimates will be improved. We will also compare pre- versus post-Xpert 12-month all-cause mortality rates.

Table 3 below summarizes the study questions and the study populations that will be used to answer these questions.

Table 3: Summary of Study Questions and Patient Populations

Study Question	Description of Outcome to be Compared	Outcome Type	Study Populations which will be Compared: Pre-Intervention:	Study Populations which will be Compared: Post-Intervention:	Basic Analysis
**Primary Study Questions**:					
1	TB diagnostic algorithm sensitivity among HIV-infected adult TB suspects newly enrolling in care.	Sensitivity proportion	Study Population A	Study Population B	Comparison of two independent sensitivity proportions
2	Adult all-casue mortality in the first 6 months of ART	Mortality rate	Study Population R	Study Population B	Comparison of two independent rates
**Secondary Study Questions**					
	**(a) Intensified TB Case Finding:**				
1	Proportion of new adult ART patients screened for TB	Proportion	Study Population R	Study Population A	Comparison of two independent proportions
2	Proportion of adult ART patients screening positive	Proportion	Study Population R	Study Population A	
3	Proportion of new adult ART patients, who started ART while on TB treatment	Proportion	Study Population R	Study Population A	
4	TB diagnostic algorithm sensitivity among HIV-infected pediatric TB suspects newly enrolling in care	Sensitivity proportion	Study Population A	Study Population B	Comparison of two independent sensitivity proportions
5	Sensitivity of the MOH-recommended 6 symptom TB screen for detecting culture positive TB disease.	Sensitivity proportion	Study Population A	Study Population B	Sensitivity of MOH-recommended 6-symptom algorithm in detecting culture positive TB.
5	TB diagnostic sensitivity of Xpert-based algorithms including versus excluding X-ray for Xpert-negative TB suspects	Sensitivity proportion	N/A	Study Population B	Comparison of sensitivity proportions.
	**(a) Continued - Intensified Drug Resistant TB Case Finding:**				
6	Increased sensitivity of the Xpert-based TB diagnostic algorithm in identifying drug resistant TB.	Sensitivity proportion	Study Population A	Study Population B	Comparison of two independent sensitivity proportions
7	Positive predictive value (ppv) of Xpert diagnosis of RIF resistance in identifying MDR TB.	PPV proportion	N/A	Study Population B	Single proportion

8	PPV improvement if TB drug resistance risk factors are used in combination with the Xpert MTB/RIF assay to identify: (1) MDR TB; (2) Rifampicin resistance.	PPV proportion	N/A	Study Population B	Comparison of ppv proportions
	**(b) HIV Patient Outcomes:**				
1	Adult ART patient TB incidence in the first 6 months of ART.	Mortality rate	Study Population R	Study Population B	Comparison of two independent mortality rates
2	Adult TB treatment outcomes among HIV clinic enrollees diagnosed with TB in the first 6 months after clinic enrollment	Proportion cured/completed TB treatment	Study Population R*	Study Population B	Comparison of two independent proportions (incidence risks)
3	Adult ART patient hospitalization incidence in the first 6 months of ART	Hospitalization rate	Study Population R	Study Population B	Comparison of two independent incidence rates
	**(c) Best Practices for Xpert Package Scale-up**				
1	Xpert device performance according to its location	Sensitivity proportion	N/A	Study Population B	Comparison of independent sensitivity proportions
2	Proportion of Xpert device batch attempts for which Xpert was considered inoperable	Proportion	N/A	Study Population B	Single proportion
3	Median duration in hours Xpert was considered inoperable	Median count	N/A	Study Population B	Median count
4	Frequency of reasons for being inoperable	Proportions (frequency distribution)	N/A	Study Population B	Proportions (frequency distribution)
5	Frequency of reasons for being inoperable by site (point-of-care versus laboratory-situated)	Proportions (frequency distribution)	N/A	Study Population B	Comparison of frequency distributions
6	Monthly number of days Xpert was inoperable	Mean and Median count	N/A	Study Population B	Mean and Median count
	**(d) Laboratory Issues Surrounding Use and Performance**				
1	Median time from screening positive for TB, to: (i) specimen collection; (ii) specimen receipt at the testing site.	Mean and Median time	~~Study Population R~~N/A	Study Population B	**Report** independent means or medians
2	Median time from sputum/gastric aspirate collection to: (i) result availability at the laboratory;	Mean and Median time	Study Population A	Study Population B	Comparison of independent means or medians

	(ii) result return to the patient folder; (iii) TB diagnosis (clinician decision); (iv) result return to the patient; and (v) initiation of TB treatment				
3	Performance (sensitivity, specificity, positive predictive value, negative predictive value) change over time	Proportions	N/A		Comparison of sequential monthly proportions
4	Proportion of newly diagnosed TB patients who are lost to follow-up before they can receive the TB diagnosis and start TB treatment	Proportions	Study Population A	Study Population B	Comparison of independent proportions

*TB treatment outcomes will be obtained from TB clinic electronic registers for TB patients, who started HIV care and treatment at study clinics in the 24 months prior to study start (see retrospective study procedures).

3.2. Rationale for Proposed Design

The rationale for: (1) the step-wedge design, and (2) the need for retrospective data collection, is described below.

(a) Nested Step-Wedge Design

Xpert has been proven a superior TB diagnostic process compared with sputum smear microscopy, and is currently recommended by the WHO for use in resource-limited settings for HIV-infected persons and in settings where TB drug resistance is common.[7] Although the entire Xpert-based TB diagnostic algorithm for HIV-infected persons has not yet been proven superior to the existing Botswana TB diagnostic algorithm (which includes use of chest radiograph for smear-negative TB suspects), it is anticipated that the Xpert-based algorithm will have greater sensitivity than the existing algorithm.[8] To meet the dual objectives of (1) implementing use of the Xpert-based algorithm as fast as is feasible; and (2) evaluating the potential superiority of the Xpert-based algorithm over the existing algorithm, the Botswana MOH has chosen to sequentially but rapidly implement 13 Xpert devices in service of 22 HIV clinics over a period of nine months, initiating one or two Xpert devices per month which equates to one, two, three, or four clinics being initiated per month (see figure 3 and Appendix 3). Even if there were no need to evaluate the superiority of the Xpert-based algorithm over the existing algorithm, the MOH would choose a phased approach to implementation of the Xpert device for feasibility reasons. Set-up of the Xpert device, and training in its use, at any site (laboratory or health facility) requires considerable

investment of financial and human resources and a phased rollout of the device is considered the most reasonable approach to implementation.

The main advantage of a stepped wedge design over a simple pre-post evaluation is that the stepped wedge design offers a number of opportunities for data analysis, particularly for modeling the effect of time on the effectiveness of an intervention.[36] Since the 1987 Gambia Hepatitis Study,[37] it has been used successfully to evaluate complex interventions including novel TB diagnosis and treatment interventions[38, 39] in resource-limited settings.

(b) Prospective and retrospective data collection methods

Prospective data collection is the only way to answer the first primary study question which is related to performance of the Xpert-based TB diagnostic algorithm. However, retrospective data collection is needed to answer the second primary study question (Are all-cause mortality rates in the first 6 months of ART significantly different before and after implementation of the full Xpert package?). This is because we hope to compare all-cause mortality during the first 6 months of ART prior to implementation of both the *complementary TB case finding interventions and* the Xpert device, with the 6-month all-cause mortality rate observed after rollout of the *full* Xpert package at selected sites. We believe that retrospective data collected on all-cause mortality during ART will be of reasonable quality because: (1) all ART patients have a permanent medical record located at HIV care and treatment facilities, which is completed at all follow-up visits; (2) this standard medical record has a data field for final patient outcome status, which should be routinely completed by the attending clinician; and (3) Botswana ART guidelines recommend tracing of ART paitents using telephone or house visits if they are late for appointments – this ensures that the proportion of patients lost to follow-up is much lower than in other countries [40]. As part of this study, patients in the retrospective cohort, who are late for their last scheduled appointment, and who have not been traced, will be traced according to Botswana national guidelines.

3.3. Study Population

(a) **HIV Care and Treatment Facilities:** The main reason for selecting 21 of the 22 clinics is that they have a high ART patient enrollment rate (≥8 ART patients per month) (Appendix 3). One clinic (Gantsi) was selected mainly because, according to the Ministry of Health, it has a high prevalence of MDR TB among HIV clinic enrollees and MOH believes these patients will benefit from early rollout of the Xpert device. Other factors which prompted investigators to choose these 22 sites include:

- All 22 clinics have at least one year's experience in providing ART services.
- Nine of the clinics have no on-site laboratory or trained laboratory staff, while the other 13 clinics have some on-site laboratory facilities or easy access to a nearby laboratory facility. This is representative of HIV care and treatment centers in Botswana.
- All sites currently implement the pre-Xpert TB diagnostic algorithm, although adherence to algorithm guidelines and algorithm diagnostic performance has not yet been assessed.
- All sites have the ability to perform testing, or to transport specimens for, hematology, serum chemistry, and CD4 count analysis.

(b) Patient Population:

The study population will include males and females of all age groups (pediatric cases will be included) with HIV infection, who fall into two broad categories:

Patients enrolled prospectively:

- All patients newly registered at each study clinic in the prospective (2-10 months) enrollment period **prior** to *full* Xpert package implementation.
- All patients newly registering at each study clinic in the prospective enrollment period **after** *full* Xpert package implementation. [The duration of post-Xpert enrollment will be determined by sample size estimations to answer the study questions (see below)].
- **Inclusion criteria for prospective cohorts**:
  - All consenting adult patients (we use the legal definition of adult: ≥18years old) newly registered in the prospective period.
  - All persons newly registered in the prospective period and aged 7-17 who assent to enrollment and for whom the guardian provides consent for enrollment.

- All children <7 years old newly registered in the prospective period and for whom consent for enrollment has been provided by the guardian.

- **Exclusion criteria for prospective cohorts**:
  - Patient (or patient's guardian if patient is <18 years old) does not provide consent.
  - Patients aged 7-17 years old who do not provide assent.
  - Patient (or patient's guardian if patient is <18 years old) declines to provide contact information for themselves.
  - Patient (or patient's guardian if patient is <18 years old) declines to allow study staff to contact them by phone and in person if they miss a study visit.
  - All prisoners.

Patients enrolled in the retrospective cohort:

- **Inclusion criteria for retrospective cohorts**:
  - All ART patients (all ages) who initiated ART in the 24 months prior to study start-up (which is considered the date of initiation of prospective enrollment).
- **Exclusion criteria for retrospective cohorts**:
  - At the single interview, the patient (or patient's guardian if patient is <18 years old) does not provide consent for inclusion in the study.
  - At the single interview, patients aged 7-17 years old do not provide assent.
  - All prisoners (any patients whose records might reasonably identify them as prisoners (e.g., a correctional facility is listed as the patient's address) would be excluded from the retrospective cohort.

Note: All patients eligible for the retrospective cohort, who are alive and on ART at the time of study initiation, will be eligible for a one-time cross-sectional interview conducted after the patient's first scheduled visit post study initiation.

(c) Justification of Exclusion and Inclusion of Sub-segments of the Population

- **Inclusion of Children**

Children of all age groups will be included in the study for two reasons: (1) like HIV-infected adults, more HIV-infected children with active TB are likely to be identified by Xpert than smear microscopy; and (2) few studies have examined Xpert performance in a pediatric population. The study accounts for the fact that procedures for diagnosing tuberculosis in children are significantly different from that of adults (e.g., gastric aspirate is an option for sputum collection in young children).

- **Inclusion of Pregnant women**

  Pregnant women will not be excluded from this study. As in non-pregnant adults, TB is a leading cause of death in HIV-infected pregnant women, and pregnant women are likely to benefit from Xpert package rollout. The only risk to the fetus from the procedures performed in this study would be from chest radiography. The International Commission on Radiological Protection (IRCP) issued a report in 1999 on the risks to pregnant women and their fetuses associated with chest radiography and other diagnostic medical procedures that use radiation[24]. The IRCP states that a single chest radiograph does not present any risk to pregnant women or fetuses, because the ionizing dose of radiation from a single chest radiograph is several thousand times less than the dose of radiation associated with fetal malformations or childhood leukemia. The IRCP report also does not recommend routine testing for pregnancy prior to chest radiography. Nevertheless, in Botswana, it is common practice, not to perform chest radiography in women known to be pregnant. Therefore, for all women of child-bearing age, if chest X-ray is required, we will perform pregnancy testing, and we will not perform a chest radiograph for those that are pregnant.

- **Exclusion of Prisoners from the Study**

  Most prisoners in Botswana receive their health care, including HIV care, at specialized facilities; therefore, we anticipate that few prisoners will present for possible inclusion in the study. If this eventuality does occur, prisoners will not be enrolled as it is difficult to assure their follow-up since they are sometimes moved to different facilities without warning, or if they are released, they can choose move to a different part of the country.

The figure below describes the patient populations of interest for each clinic selected for the study.

Figure 4: Diagrammatic Representation of Patient Populations of Interest at Each Selected Clinic

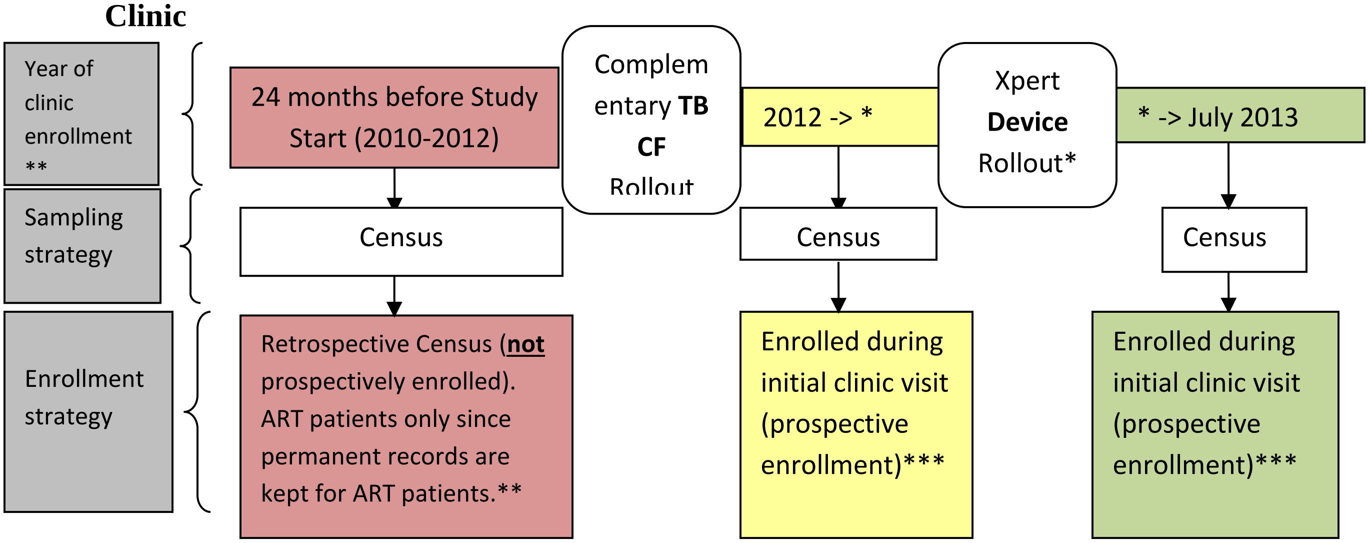

*Timing of Xpert package rollout will depend on allocation sequence.
**Red color reflects need for retrospective data collection among prior ART enrollees.
***In green and yellow blocks, clinic enrollment refers to ART and pre-ART care enrollment. The yellow color reflects pre-Xpert prospective enrollment and data collection, and green color is post-Xpert prospective enrollment and data collection.

3.4. Randomization Procedures

After obtaining consent from selected clinic and laboratory chiefs, an independent statistician, located within the National Center for Global Health (NCGH) at the US Centers for Disease Control and Prevention (CDC) will be asked to randomly assign the rollout order of the 13 Xpert devices. Note that some of the clinics use the same TB diagnostic facility as is indicated in Appendix 3. For example, Letsholathebe II Memorial Hospital, Boseja clinic, and Maun clinic, in Ngami, Maun district, all use the same TB lab, where the Xpert device will be located. Therefore, these three clinics will start implementing the full Xpert package together and will comprise one step. If there are 13 Xpert devices to be allocated in 9 steps, there are about 9! (or 362,880) possible permutations of the order of Xpert device rollout. The statistician will randomly choose one of these permutations. Once the Xpert device rollout order has been randomly chosen, the statistician will inform study investigators from CDC, MOH, and the clinic

and laboratory chiefs, simultaneously via a conference call or meeting to ensure transparency of the randomization process.[35]

3.5. Sample Size

Sample size estimates will be based on the two primary study questions. The first primary research question is: **Is the Xpert-based TB diagnostic algorithm for adults more sensitive than the pre-Xpert TB diagnostic algorithm in diagnosing culture-positive TB disease?**

These sample size calculations require estimates of:

(a) The proportion of new HIV clinic enrollees that will screen positive for TB using the WHO-recommended four-symptom screen.

(b) The estimated sensitivity of the pre-Xpert TB diagnostic algorithm in diagnosing true TB among TB suspects in Botswana.

(c) The estimated sensitivity of the Xpert TB diagnostic algorithm.

(d) The intra-cluster correlation coefficient (ICC) appropriate for our design.

Table 3 below lists these required sample size assumptions derived from a literature review.

Table 3: Literature Review for Primary Question Sample Size Calculations: Sensitivity of Pre-Xpert TB Diagnosis Algorithm Vs. Xpert-based TB Diagnosis Algorithm

	**Fractions of Sub Populations**	**Cumulative % of Total Enrollees**	**Reference**
**Pre-Xpert TB Diagnostic Algorithm:**			
(a) % of HIV Clinic Enrollees who Screen Positive for TB	49.1%	49.1%	Getahun *et al*, PlosMed 2011[34]
(b) Of TB suspects (a), % with true TB	33.0%	16.2%	See table 4
(c) Of TB suspects with true TB (b), % diagnosed with TB using methods other than liquid culture (Pre-Xpert Sensitivity)	62.5%	10.1%	Shah *et al,*[8] JAIDS 2009
**Xpert-based TB Diagnostic Algorithm**			
(a) % of HIV Clinic Enrollees who Screen Positive for TB	49.1%	49.1%	Getahun *et al*, PlosMed 2011[34]
(b) Of TB suspects (a), % with true TB	33.0%	16.2%	See table 4
(c) Of TB suspects with true TB (b), % diagnosed with TB using methods other than liquid culture (Pre-Xpert Sensitivity)	82.4%	13.4%	Boehme *et al*, Lancet 2011[7] (Publication Table 5)

Table 4: Literature Review to Estimate the True Prevalence of TB Among new Adult HIV Clinic Enrollees

Year	Author	Country	Study population	Gold standard	Total enrollees	No. of TB Cases at Enrollment	TB prevalence
2011	Lawn[41]	South Africa	PLHIV enrolling in ART	Liquid culture	468	81	17.3%
2010	Bassett[42]	South Africa	PLHIV entering ART education program	MGIT	825	128	15.5%
2009	Lawn[43]	South Africa	PLHIV >18 Referred to Community based ART	2-4 MGIT	226	58	25.7%
2010	Lawn[12]	South Africa	PLHIV initiating ART	MGIT	241	76	31.5%
2011	Shah[8]	Ethiopia	PLHIV enrolling in HIV care & Tx	L J	453	32	7.1%
2011	Kain[44]	Cambodia, Vietnam, Thailand	PLHIV enrolling in HIV care & Tx	Liquid culture	1748	267	15.3%
**Crude Average**					**3,961**	**642**	**16.2%**
**Median**							**17.3%**

Abbreviations: PLHIV, people living with HIV; ART, antiretroviral therapy; MGIT, Mycobacteria Growth Indicator Tube; LJ, Lowenstein Jensen's culture medium

The size of the cohort of new HIV clinic patients enrolled prior to Xpert package rollout is constrained by the ethical need to rollout the package as fast as is feasible for the benefit of the HIV-infected patients newly entering care. We estimate that the Xpert device rollout can start in early 2012. This restricts the pre-Xpert prospective enrollment phase to about 2 months for those clinics randomly selected to implement the Xpert package **first** (see figure 3 for illustration).

The average number of patients starting ART at each clinic each month is 23/month (Appendix 3). A literature review suggests that about 75% of patients, newly presenting to HIV clinics in sub-Saharan Africa, are eligible for ART at CD4<200 cells/uL.[18] A physician at one of the larger HIV care and treatment facilities in Botswana (Princess Marina Hospital in Gaborone) estimated that about 70% of patients presenting to the clinic were ART eligible at CD4<250 cells/uL. If we assume that the 23 ART-eligible patients represent 70% of new HIV clinic enrollees, a total of 33 patients could be enrolling at each of the 22 clinics each month. This would allow for a total of 4,686 patients enrolled prior to Xpert package rollout.

Because we cannot accurately predict monthly study enrollment rates, to be conservative with sample size calculations, we will assume that 23 patients will enroll at each clinic per month, for a maximum pre-Xpert patient enrollment of 3,266 consenting new clinic enrollees.

Funding restrictions mean that study enrollment at all 22 clinics after Xpert device rollout can only be supported for 19 months. If enrollment continues through 19 months at 23 patients per month, this would generate a post-Xpert sample size of 6,348. We explored how power to detect a difference in sensitivity of the Xpert-based diagnostic algorithm compared to the pre-Xpert algorithm varied over a range of possible sensitivities if enrollment continued through 19 months. We simulated data according to the stepped wedge design above, using the beta-binomial model to induce the ICC. We simulated 1,000 datasets and fit a mixed model appropriate for the stepped wedge design (Hussey and Hughes 2007)[45] that included fixed effects for time and intervention condition (0 for time points before Xpert implementation and 1 afterward), and a random effect for the clinic, to take into account between-clinic variability. We assumed 23 patients per month throughout the study period, with approximately 50% treated as TB suspects, and approximately one-third of those as having true TB (see Table 3 for rationale). Table 5 presents power for several combinations of sensitivity pre- and post-Xpert. For the parameters shown in table 3, we have excellent power to detect the intervention effect (98.9%). Even with a slightly smaller intervention effect, power is still high.

Table 5: Power to Detect Differences in Sensitivity Comparing pre-Xpert to post-Xpert

Sensitivity Pre-Xpert	Sensitivity Post-Xpert	Power to detect difference
62.5	82.5	**99.6%**
62.5	80.0	**97.8%**
62.5	77.5	**94.0%**
58.0	78.0	**99.2%**
58.0	75.0	**96.7%**
58.0	73.0	**91.9%**
55.0	75.0	**98.9%**
55.0	71.9	**96.2%**
55.0	70.0	**89.7%**

Note: assumes an ICC of 0.05, two-tailed test with α=0.05

Figure 5: Diagram of Patient Enrollment to Meet Sample Size Requirement for the Primary Study Question (Note that prospective enrollment will continue for 19 months from the date of study start).

	Retrospective Baseline	2012								2013													Total Prospective Enrolment
		*May '12	June '12	July '12	Aug '12	Sep '12	Oct '12	Nov '12	Dec '12	Jan '13	Feb '13	Mar '13	Apr '13	May '13	June '13	July '13	Aug '13	Sep '13	Oct '13	Nov* '13	Pre-Xpert	Post-Xpert	
#1	552	23	23	23	23	23	23	23	23	23	23	23	23	23	23	23	23	23	23	23	46	391	437
#2	552	23	23	23	23	23	23	23	23	23	23	23	23	23	23	23	23	23	23	23	46	391	437
#3	552	23	23	23	23	23	23	23	23	23	23	23	23	23	23	23	23	23	23	23	69	368	437
#4	552	23	23	23	23	23	23	23	23	23	23	23	23	23	23	23	23	23	23	23	69	368	437
#5	552	23	23	23	23	23	23	23	23	23	23	23	23	23	23	23	23	23	23	23	92	345	437
#6	552	23	23	23	23	23	23	23	23	23	23	23	23	23	23	23	23	23	23	23	92	345	437
#7	552	23	23	23	23	23	23	23	23	23	23	23	23	23	23	23	23	23	23	23	115	322	437
#8	552	23	23	23	23	23	23	23	23	23	23	23	23	23	23	23	23	23	23	23	115	322	437
#9	552	23	23	23	23	23	23	23	23	23	23	23	23	23	23	23	23	23	23	23	115	322	437
#10	552	23	23	23	23	23	23	23	23	23	23	23	23	23	23	23	23	23	23	23	138	299	437
#11	552	23	23	23	23	23	23	23	23	23	23	23	23	23	23	23	23	23	23	23	138	299	437
#12	552	23	23	23	23	23	23	23	23	23	23	23	23	23	23	23	23	23	23	23	161	276	437
#13	552	23	23	23	23	23	23	23	23	23	23	23	23	23	23	23	23	23	23	23	161	276	437
#14	552	23	23	23	23	23	23	23	23	23	23	23	23	23	23	23	23	23	23	23	184	253	437
#15	552	23	23	23	23	23	23	23	23	23	23	23	23	23	23	23	23	23	23	23	184	253	437
#16	552	23	23	23	23	23	23	23	23	23	23	23	23	23	23	23	23	23	23	23	207	230	437
#17	552	23	23	23	23	23	23	23	23	23	23	23	23	23	23	23	23	23	23	23	207	230	437
#18	552	23	23	23	23	23	23	23	23	23	23	23	23	23	23	23	23	23	23	23	207	230	437
#19	552	23	23	23	23	23	23	23	23	23	23	23	23	23	23	23	23	23	23	23	230	207	437
#20	552	23	23	23	23	23	23	23	23	23	23	23	23	23	23	23	23	23	23	23	230	207	437
#21	552	23	23	23	23	23	23	23	23	23	23	23	23	23	23	23	23	23	23	23	230	207	437
#22	552	23	23	23	23	23	23	23	23	23	23	23	23	23	23	23	23	23	23	23	230	207	437
**Pre-Xpert**	12,144	506	506	460	414	368	299	253	207	161	92	0	0	0	0	0	0	0	0	0	3,266		
**Post-Xpert**		0	0	46	92	138	207	253	299	345	414	506	506	506	506	506	506	506	506	506		6,348	
**Total**	12,144	506	506	506	506	506	506	506	506	506	506	506	506	506	506	506	506	506	506	506			9,614

Key	Retro- or Prospective Enrollment?	Complementary TB Case Finding Intervention in place?	Xpert device in place?
(pink)	Retrospective	No	No
(yellow)	Prospective	Yes	No
(light green)	Prospective	Yes	Yes

(dark green, red border) Indicates initiation of one Xpert device. There are 13 Xpert devices.

Complementary TB Case Finding Intervention:

(1) Adoption of the WHO-recommended 4-symptom screen (cough of any duration, fever, weight loss, and night sweats) for TB screening among HIV-infected adults (>12 years old);
(2) Introduction of clinic TB case finders, responsible for daily TB screening, sample collection, sample transport to the laboratory, and reporting of results to diagnosing clinicians; and,
(3) Training for health care workers in correct use of recommended case finding algorithms for adults (>12 years) and children (<12 years).

The second primary study question is: **Are pre- versus post-Xpert package all-cause mortality rates in the first 6 months of ART significantly different? (Note that this compares mortality rates during months 0-6 of ART between ART patients enrolled cohorts "R" versus "B")**

As described earlier, the pre-Xpert package all-cause ART mortality rates during the first 6 months of ART will be estimated using retrospective data collection methods. The size of the retrospective cohort to be evaluated for TB incidence needs to be estimated to allow the study to answer the question with sufficient power.

Sample size calculations require the following estimates from a literature review:

(a) All-cause ART mortality before and after Xpert package rollout

(b) ICC

To estimate power for the comparison of incidence in the retrospective cohort to that found in the prospective cohort, we followed the approach of Moulton et al. (2007), in which they adapted a formula for cluster-randomized trials to the stepped wedge design.

Table 9: Sample Size Calculations

Potential all-cause mortality rates in the first 6 months of ART		Reduction	alpha	Size of Retrospective Cohort	Prospective Cohort determined by Primary Question #1	Power
Pre-Xpert (Per 100 PY)	Post-Xpert (Per 100 PY)					
**5[46]**	**2.50**	**50%**	0.05	months: **24** size: **12,144**	23 per month (22 clinics)	**84.7%**
**10**	**5.00**					**96.4%**
**15**	**7.50**					**98.8%**
**20**	**10.00**					**99.4%**
5	3.00	**40%**	0.05	months: **24** size: **12,144**	23 per month (22 clinics)	62.5%
**10**	**6.00**					**82.1%**
**15**	**9.00**					**89.5%**
**20**	**12.00**					**93.0%**

In Mozambique, the documented all-cause early mortality rate (within the first 90 days) was 12.9 deaths per 100 patient-years. Programs with better tracing programs generally have higher documented early all-cause mortality rates (e.g. Zambia [18], China [47], and a meta-analyses of 18 programs in resource-constrained settings [17] have reported early all-cause mortality rates of 26, 23, and 14.7 deaths per 100 patient-years, respectively). In Botswana,

limited data from 2008 suggest that the documented all-cause early mortality rate in the first 6 months of ART among adults was about 15 deaths per 100 PY.[40] Table 9 shows that a potential 40% reduction in all-cause mortality [1] would be detected with sufficient power (>80% power).

Table 9.1. shows sample size calculations for comparison of 6 month ART mortality rates in cohort A versus cohort B. Only if pre-Xpert prospective ART mortality rates are as high as 20 per 100 PY and fall by 80% to 4 per 100 PY, will there be sufficient power to detect a difference in TB incidence rates in cohorts A versus B. Therefore, because we do not expect mortality rates as high as 20 per 100 PY, or a reduction in mortality rates by >50%, it is unlikely that we will be able to detect a significant difference in 6-month mortality rates **between cohorts A and B.**

Table 9.1. Table of Power to Detect a Difference in TB incidence Rates during Months 0-6 of ART Between Cohorts A and B

TB incidence in the first 6 months of ART		Reduction	alpha	Size of PRE-EXPERT	Prospective Cohort POST EXPERT ROLLOUT	Power
Pre-Xpert (Per 100 PY)	Post-Xpert (Per 100 PY)					
**5**	**1**	**80%**	0.05	23 per month (22 clinics)= 3,266 pts	23 per month (22 clinics) = 6,348 pts	0.30
**10**	**2**					0.52
**15**	**3**					0.69
**20**	**4**					**0.80**
**5**	**1.5**	**70%**	0.05			0.24
**10**	**3**					0.42
**15**	**4.5**					0.57
**20**	**6**					0.68
**5**	**2**	**60%**	0.05			0.19
**10**	**4**					0.32
**15**	**6**					0.44
**20**	**8**					0.55
**5**	**2.5**	**50%**	0.05			0.14
**10**	**5**					0.24
**15**	**7.5**					0.33
**20**	**10**					0.41
**5**	**3**	**40%**	0.05			0.11
**10**	**6**					0.17
**15**	**9**					0.22
**20**	**12**					0.28

Note: an intracluster correlation coefficient of 0.05 is used based on a literature review including:

- Yelland LN, Salter AB, Ryan P, Laurence CO. Adjusted intraclass correlation coefficients for binary data: methods and estimates from a cluster-randomized trial in primary care. Clin Trials. 2011; **8**(1): 48-58.Parker DR, Evangelou E, Eaton CB. Intraclass correlation coefficients for cluster randomized trials in primary care: the cholesterol education and research trial (CEART). Contemp Clin Trials. 2005; **26**(2): 260-7.

4. Study Procedures

4.1. Site Assessments

Assessments of the clinic sites and reference laboratories involved in this study will be undertaken prior to the commencement of the study by the investigators. The site assessments will be performed to identify potential barriers to successful implementation of the study (e.g. malfunctioning X-ray machines). Once identified, these barriers will be discussed by project investigators. If improvements to clinics need to be made, besides interventions encompassed by the Xpert package, these improvements will be documented and reported in any reports and publications because they may have an independent effect on primary study outcomes (e.g. repair of an X-ray machine).

4.2. Training

After obtaining ethical approval for the protocol and consent from the clinic chiefs, all personnel involved in the study and use of the Xpert-based diagnostic algorithm will be trained in study procedures. Personnel to be trained include: (1) TB case finding nurses, who will also serve as the study nurses, at each clinic (study nurses will be responsible for enrollment procedures and data collection), (2) other HIV clinic personnel involved in HIV-infected patient management, (3) personnel who will operate the Xpert device, and (4) data entry personnel.

(a) **Study nurses** will be given extensive training on all study-related procedures, including consent, data collection, data storage, sample storage, sample transport, and questionnaire transport protocols. These procedures are described in detail in the next section [section 4 (c)

and (d)]. The study nurse will need to interact with other clinic personnel on a daily basis and will be encouraged to attend clinic staff meetings to ensure integration with the clinic teams. Study nurses will be trained on three occasions prior to study initiation: (1) once at a week-long training in Gaborone; (2) once immediately prior to study start; and (3) once immediately prior to GeneXpert rollout. In addition, depending on funding availability quarterly, biannual or annual meetings for study nurses will be held to improve coordination of study procedures across sites, and maintain team cohesion and morale.

(b) **Training for HIV clinic personnel** involved in various aspects of care of the HIV-infected patients will include:

- **Chest radiography training** – Medical staff who interpret chest radiographs will be given refresher training in the definitions of radiologic manifestations of TB and in the proper completion of the radiographic data collection forms.
- **Blood collection** – All staff performing phlebotomy will be provided training concerning the correct tubes to use for each requested test as well as correct procedures for labeling and storage of specimens after collection of blood.
- **Sputum collection** – The clinic staff will be trained on how to instruct the participant on the correct procedure for collecting an adequate sputum specimen. Careful instruction will ensure that sputum and not saliva is collected. Instruction will include rinsing the mouth prior to production of the sputum and breathing deeply before coughing up sputum. Instruction will also cover aspects of infection control such as the use of well ventilated areas away from other clients and staff during sputum production. Instruction will also include careful documentation of TB screening results inside permanent routine patient records as well as documentation of actions taken (i.e. specimens sent to the lab).
- **Training frequency**: Clinic personnel will be trained twice: (1) once immediately prior to study start at the clinic; and (2) once immediately prior to GeneXpert rollout.

(c) **Training for laboratory staff** will include:

- **Good laboratory practice** – All laboratory personnel will receive training in standard good laboratory practice in order to ensure that the laboratory analyses to be carried out during the study follow the correct standard operating procedures.

- **AFB smear microscopy** – After an initial assessment of capabilities, the responsible laboratory personnel will receive refresher training in preparation of slides, staining of slides and reading techniques.
- **Safe specimen handling** – The laboratory personnel will receive training in the proper preparation of specimens for transport and the use of appropriate transportation supplies to avoid spillage.
- **Specimen transport** – Training will be provided in the correct procedures for the transportation of biologic specimens between the study sites and the reference laboratories. This will include the correct packaging materials as well as the procedures required to obtain the approvals for the transportation of biologic specimens from national authorities.
- **Specimen forms, reporting** – Training will be provided in the use of the study specific forms and other data capture instruments to ensure that all data collected is accurate and reported in a timely manner to the relevant study and clinical staff.
- **Xpert MTB/RIF training -** Xpert MTB/RIF testing will be conducted either in laboratories associated with each facility or in designated point-of-care clinic locations. In both instances the relevant staff will receive specialized training in the conduct of Xpert MTB/RIF testing. This will consist of a two-day session organized by staff of the CDC-GAP International Laboratory Staff. Trainees will be required to successfully test a proficiency panel of samples.
- **Reference Laboratory training -** The reference laboratory staff will receive training in good laboratory practice as well as specific training related to the technical procedures to be carried out in the laboratory. The training will include the procedures to follow from receipt, documentation and accessioning of the specimens, through the analytic processes and reporting of the results. The trainings will be provided by in-country experts, or by various partners, including one or more of the following: American Society for Microbiology (funded through the Country Operational Plans), Becton-Dickinson Public Private Partnership, GAP CDC International Lab Branch, and Foundation for Innovative New Diagnostics (FIND).

- **Frequency of training:** Laboratory staff at the peripheral and central labs will receive one in depth training at the central laboratory in Gaborone prior to study start, followed by refresher trainings for peripheral lab staff at the start of study enrolment and again at the time of GeneXpert rollout. Central laboratory personnel will receive refresher trainings biannually.

4.3. Prospective Study Procedures

(a) **Patient Arrival at the Clinic**: Normal procedures for new HIV clinic enrollees involve: (1) patient registration at the administrative desk (this involves the client providing his/her name, proof of citizenship, and residential address and being issued a patient-held health card); (2) the patient being seen by the clinic nurse who weighs the patient, and records vital signs (pulse rate, blood pressure, temperature) on the patient-held card; (3) the patient seeing the clinician who administers routine HIV care, including: clinical history taking, physical examination, and collection of blood samples for CD4+ T-cell count, full blood count, and serum chemistry; and (4) the patient being referred, with his/her permanent facility medical record and/or patient health card to the TB case finder who will screen for TB symptoms and take sputa for smear microscopy (or Xpert).

Although all clinic personnel will be aware of study procedures, it is the study nurses (TB case finders) who will be primarily responsible for assuring adherence to study procedures such as enrollment, systematic TB symptom screening and TB diagnostic algorithm measures. All clinic personnel will check that the patient has been offered the opportunity to be enrolled in the study but the study nurse will provide detailed information about the study and perform the consent procedures. If the clinic personell prefer to change the order of patient flow to improve feasibility of study management (e.g. study nurses screen and enroll patients after the patients have been seen by the clinic nurse and while they wait for their doctor's visit) this will be documented for study supervisors. Study nurses will ensure all questions relevant to the enrollment questionnaire (see below) are completed.

Note also that the TB screening algorithms – both the pre-Xpert and Xpert – are to be implemented at the facility level, and that all patients being seen at participating facilities

will be screened using the implemented algorithms, regardless of study eligibility or participation.

(b) **Consent Procedures**: The study nurses will ask all new clinic enrollees for consent to be included in the study. Adults (legally defined as $\geq$18 years old) will be asked for written consent. Guardians of new clinic enrollees aged 0-17 will be asked for written consent for enrollment of their child and enrollees aged 7-17 will be asked for their assent for study enrollment if guardian consent is provided (see human subject's issues in section 7(a), and appendix 4 for information and consent forms).

If consent is granted, patient locator information consisting of (i) two telephone numbers and an address of the patient and (ii) a telephone number, address, and name of a patient contact person will be obtained.

(c) **Enrollment Questionnaire**: For consenting patients, the study nurse will administer the enrollment questionnaire (Appendix 5) and will abstract elements of the physical examination from the HIV-clinic medical records (i.e. the patient-held card and the permanent facility medical record), which the patient will carry into the consulting room with the study nurse.

(d) **TB Screening**: In all study sites, the study TB case-finder will administer the standardized, WHO-recommended, 4-symptom TB screen for adults (>12 years), and the existing MOH-recommended TB screen for children $\leq$12 years (described under figure 2). Those who have one or more positive symptom screens for TB will have 4 sptums collected for: (1) microscopy, (2) Flourochrome microscopy; (3) culture, (4) line probe assay if culture positive and (5) drug susceptibility testing if culture positive. All TB suspects identified after rollout of the Xpert device will also have the sputum tested with Xpert. Details of sputum processing are described below.

At the study enrollment visit only, for HIV-infected children, who are negative for all 6 TB screening symptoms, two sputa will be collected for culture to assess the sensitivity of the

screening symptoms in identifying culture positive disease. The first sputum will be collected on day 1 at the time of enrollment, and the second sputum will be collected on the morning of day 2. We will not collect sputum using a naso-gastric tube from children who are asymptomatic and unable to produce sputum, since this is considered additional risk for asymptomatic children. If an asymptomatic child cannot produce cough sputum voluntarily, we will not collect sputum from this child.

(e) **Sputa Collection**: Among those enrollees *with* one or more symptoms on TB screen, the following procedures will be followed:

- **Among persons able to produce sputa on request** (almost all symptomatic persons >12 years old) four sputum specimens will be produced as follows:
  - ➢ On the enrollment day, the patient will be asked to proceed to a designated, well-ventilated area, rinse his/her mouth with filtered or boiled water provided by the study research staff, and then produce two on-the-spot (spot) sputa in two unused containers, with each sputum >2ml in volume. The patient will also be given one clean, unused container for morning sputum collection the following day, and instructed to follow the same procedure when producing the unsupervised morning sputum. On the second day of enrollment, the patient will be asked to: (1) return to the clinic with the morning sputum already collected, and (2) produce a third spot sputum following the procedures described above. Other sputum collection procedures and infection control precautions are described in detail in the national TB diagnosis and management manual.[33]

  - ➢ **Among persons unable to produce sputa on request**, sputum-induction will be performed if contraindications are not present and if sputum-induction facilities are available:
    - Adults (>12 years): Contraindications for sputum induction among adults (>12 years old) include: Acute asthma; signs of respiratory distress including dyspnea or wheezing; hypoxia: SaO2 < 90%; abnormal vital signs; pneumothorax; fractured ribs or other chest trauma; recent eye surgery. If these contraindications are absent, procedures and infection control precautions as described in detail in

the national TB diagnosis and management manual,[33] will be followed. Adult sputum inductions will be performed to collect two sputa on the enrollment day and two sputa on the second day of enrollment.

- Children (</=12 years): Contraindications for sputum induction among children (≤12 years old) include: acute (active) asthma; any signs of moderate to severe respiratory distress; wheezing; abnormal; vital signs; epistaxis; pneumothorax; fractured ribs or other chest trauma; recent eye surgery. In addition, sputum induction is generally not performed for children <5 years old. If these contraindications are absent, procedures and infection control precautions as described in detail in the national TB diagnosis and management manual,[33] will be followed. Child sputum inductions will be performed to collect two sputa on the enrollment day and two sputa on the second day of enrollment.

  Among symptomatic children ≤12 years old who are unable to produce sputa on request, and for whom there are sputum-induction contraindications or for whom facilities for sputum-induction are unavailable, naso-gastric tube aspirates of swallowed sputum will be performed, again according to the procedures described in the national TB manual.[33]

Patients who cannot produce any sputum, despite the procedures described above, will not be excluded from follow-up, but will be excluded from the analysis of the primary study question. This situation where the study team is unable to obtain a sputum sample from a consenting TB suspect is expected to be rare. All TB diagnostic specimens, collected as part of the study, will be accompanied by a specimen collection form (Appendix 5.2)

- **Sputum Processing**:
  - **Pre-Xpert Enrollment Phase – Role of Smear Microscopy**: One of the spot samples from the first day and the spot sample from the second day will be examined for acid fast bacilli (AFB) via standard smear microscopy (Ziehl-Neelsen method).

At the six clinics where there is no on-site laboratory for microscopy, the sputa will be transported to the nearest laboratory for microscopy.

- **Post-Xpert Enrollment Phase – Role of Smear Microscopy**: The same procedures described above will be followed at the 10 clinics with on-site laboratories. This will allow comparison of the sensitivity of smear microscopy with Xpert in diagnosing culture-positive TB on the *same* sputum specimens. However, at the six clinics where there is no on-site laboratory for microscopy, the sputa will not be transported to the nearest laboratory for microscopy because there will be on-site Xpert testing available, and the additional transport of specimens for microscopy is considered to be complex and unnecessary for the study.

- **Fluorescence microscopy, Liquid TB Culture, and Drug Susceptibility Testing**: One of the spot sputum samples collected on the first day and the morning sputum sample from the second day of study enrollment will be sent to the national TB reference laboratory (NTRL) for fluorescence microcopy and liquid mycobacterial culture. Those specimens which are culture positive will be assessed for TB drug sensitivity (DST). Figure 6 illustrates an overview of patient flow from the initial study visit and figure 7 illustrates the flow of TB sputa.

- The two sputa collected from aymptomatic HIV-infected children during the enrollment visit will be sent to the national TB reference laboratory for culture.

Figure 6: Overview of Study Enrollment and Follow-up Procedures (Adults and Children)

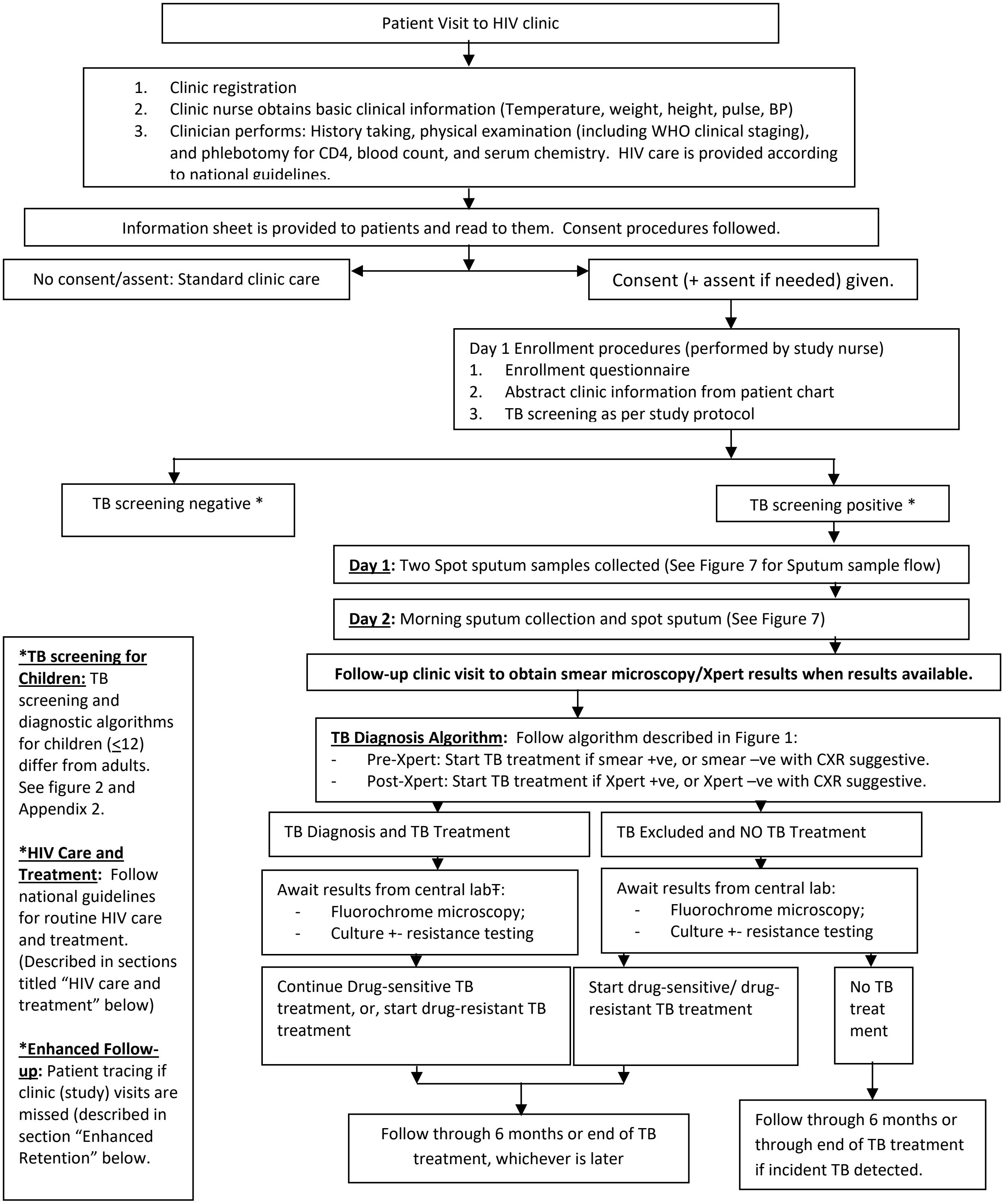

Figure 7: Flow Diagram for Sputa Mycobacterial Testing (See box for Abbreviations)

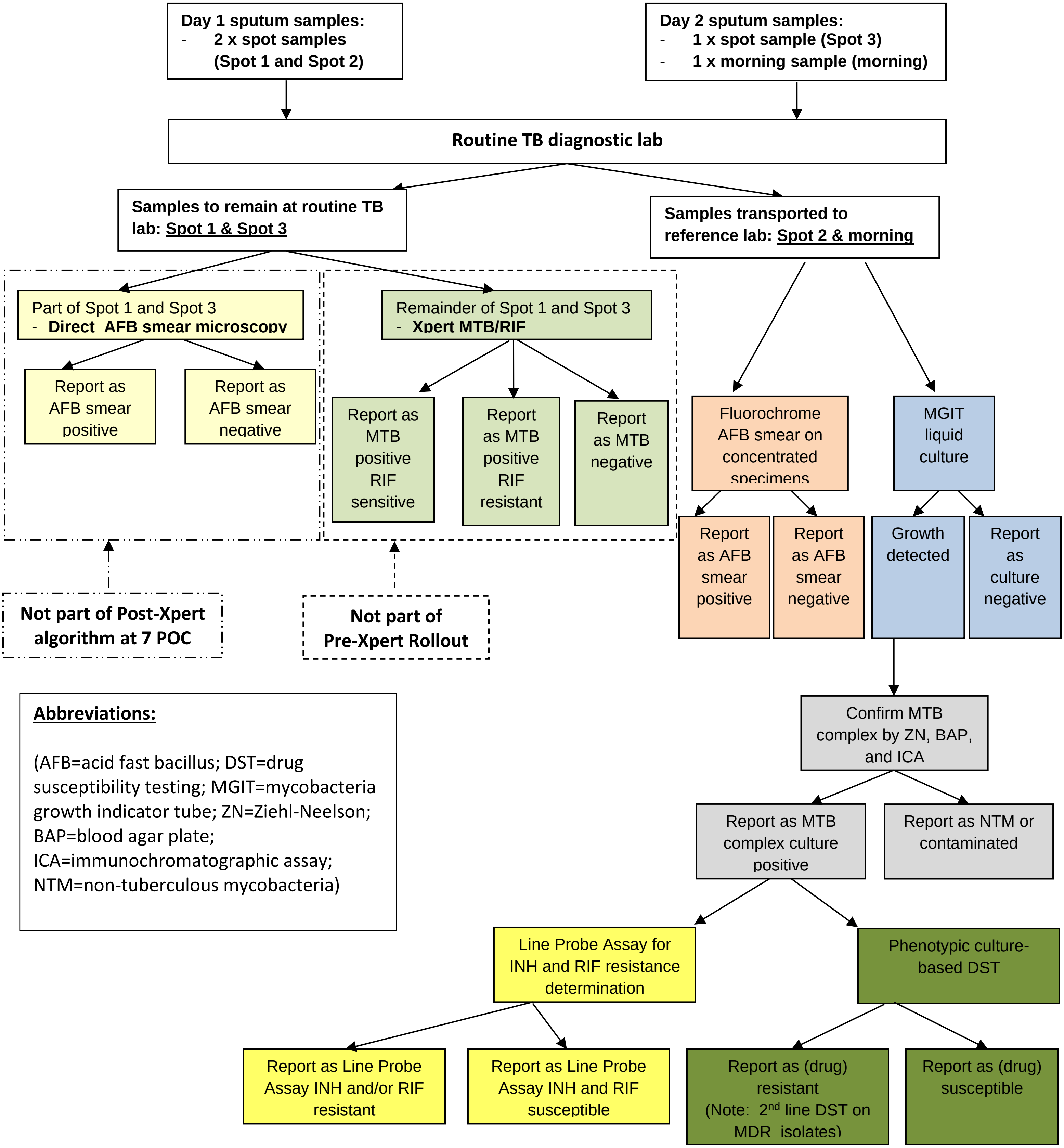

Abbreviations:

(AFB=acid fast bacillus; DST=drug susceptibility testing; MGIT=mycobacteria growth indicator tube; ZN=Ziehl-Neelson; BAP=blood agar plate; ICA=immunochromatographic assay; NTM=non-tuberculous mycobacteria)

- **<u>TB Diagnosis and Treatment</u>**
  - **Adults (>12 years):**
    - Screening: As described earlier, in this study, TB screening among adults will include screening for any of the following symptoms: (1) cough of any duration, (2) fever, (3) weight loss, or (4) night sweats. This four-symptom screen is a new WHO-recommended approach based on recent evidence.[34, 48] At the start of prospective study enrollment, these new screening criteria will replace the existing MOH screening criterion which is to screen for a ≥2 week cough. However, patients in the retrospective cohort will have been screened using the old screening criterion, assuming clinicians followed national guidelines.
    - Diagnosis: Pre- and post-Xpert algorithms for pulmonary TB diagnosis in adults in this study are illustrated in figures 1, 6, and 7 and have been described in detail above. These algorithms are in line with MOH recommendations (Appendix 1).[33] Any left-over sputum at the lab will be discarded, per national guidelines. Diagnosis of extra-pulmonary TB may rely on additional diagnostic tests as described on pages 23 and 24 of the national TB manual.[33] The most common types of extra-pulmonary TB would include lymphadenitis, pleural effusion, pericarditis and meningitis. However, any organ may be infected with TB. Once diagnosed, enrolled adult patients will fall into one of the following categories:

Table 10: Botswana TB Nomenclature

	Site of disease	Sputum Smear**	History of Previous TB	Drug resistance
**Options**	- Pulmonary<br>- Extra-pulmonary	- Smear +ve<br>- Smear –ve	New<br>Retreatment‡<br>- Relapse‡<br>- Failure‡<br>- Default‡	Drug-sensitive TB‡<br>Mono-resistant TB‡<br>Poly-resistant TB‡<br>MDR-TB‡<br>XDR-TB‡<br>**Xpert RIF-resistant*** **
**Implications**	May influence type of treatment	Important for monitoring treatment progress and outcome	- Influences choice of regimen<br>- Influences duration of treatment	- Influences choice of regimen<br>- Influences duration of treatment

*All patients will be HIV-infected which implies: (1) co-trimoxazole for all patients; and (2) ART when tolerating TB treatment, ideally early (after 2 weeks of TB treatment).[49]
**Smear results are standard smear microscopy results (Ziehl-Nielsen, not Flourochrome smear microscopy).
***Xpert resistance is an additional category which will be preliminary in this study; RIF-resistant TB, as diagnosed by Xpert, will eventually be re-categorized as drug-sensitive, mono-, poly-, multi-, or extensive- drug resistance, based on culture and drug resistance testing.

‡See appendix 6 for definitions.

- Treatment: Once classified, TB treatment guidelines as described in the national TB guidelines will be followed.[33] Table 11 summarizes the TB treatment strategies:

Table 11: Recommended Adult Treatment Regimens by Treatment Group

TB Patients		TB Treatment Regimens[a]	
		**Intensive Phase (daily)**	**Continuation Phase (daily)**
**New**	All new adult cases of TB regardless of site, smear results or severity of disease	2HRZE[b]	4HRE
**Re-treatment[c]**	Previously treated cases of TB:<br>- Retreatment after relapse<br>- Retreatment after default<br>- Retreatment after treatment failure	2HRZES/1HRZE[d]	5HRE
**MDR-TB**	Patients with confirmed or strongly suspected MDR-TB	See Chapter 7 of the national TB guidelines for details about MDR-TB treatment[33]	

Abbreviations: 1/2/4/5=number of months; H=isoniazid; R=rifampicin; Z=pryrazinamide; E=ethambutol; S=streptomycin;
[a]Direct observation of drug intake is always required.
[b]Streptomycin is an alternative to Ethambutol. Replace Ethambutol with Streptomycin for TB meningitis.
[c]Sputum for culture and DST from all retreatment patients will be collected *before* starting therapy.
[d]The retreatment intensive phase is 3 months. Streptomycin is given only for the first 2 months.

After Xpert device rollout, some patients will be diagnosed as RIF-resistant a few weeks before culture and DST results become available. This represents a new management challenge for the TB program, for which national guidelines have not yet been created. The following protocol will be followed for these patients *and* for any patient where TB drug resistance is detected:

i. Referral and early transport to the nearest MDR-TB treatment center:
   - Princess Marina Hospital, (PMH) Gaborone: 3621625
   - Nyangabgwe Referral Hospital, Francistown: 2411261
   - Sekgoma Memorial Hospital, Serowe: 4611000, ext 1337
   - Ghanzi Primary Hospital, Ghanzi: 6596333, ext 226
   - Letsholathebe II Memorial Hospital, Maun: 6879000[33]

ii. Specialist physicians at the referral treatment center will, after receiving the laboratory and chest X-ray reporting forms, decide on the initial TB treatment regimen. (See Appendix 6).[33]

If a patient is found to be Xpert positive but culture negative, decisions about future treatment will be made by the patient's physician. Consults are available at the central MDR-TB treatment centers.

- **Children (≤12 years):**
  - Screening**:** Although the WHO recommends the following TB screening criteria for children: (1) poor weight gain [reported/confirmed weight loss, flattened growth or weight-for-age z-score (WAZ) <-2]; (2) current fever; (3) current cough; and (4) contact history with a TB case,[48] the Botswana guidelines for screening are slightly different. They include screening for the following: (1) Weight loss or failure to thrive (no weight gain over 3 months); (2) Enlarged lymph nodes (more than 1 x 1 cm); (3) Cough for ≥ 2 weeks; (4) Fever for ≥ 2 weeks; (5) Fatigue/reduced playfulness ≥ 2 weeks; and (6) Profuse night sweats ≥ 2 weeks.[33] We will follow the Botswana national guidelines, because convincing evidence that the WHO recommendations are superior, is lacking.[50]

  - Diagnosis**:** Key differences of the pediatric TB diagnostic algorithm for child TB suspects compared with the adult diagnostic algorithms (pre- and post-Xpert) include:

    i. Obtaining sputum specimens is more challenging. Either sputum induction or gastric aspiration are recommended for children who cannot produce sputa (summarized earlier and described in detail in the TB manual[33]).

    ii. Three TB diagnostic tests are carried out for all TB suspects: (1) tuberculin skin test (TST); (2) chest X-ray; (3) two sputa for microscopy (or Xpert).

    The algorithm is illustrated in Figure 2, shown earlier and as appendix 2. Any left-over sputum at the lab will be discarded, per national guidelines.

  - Treatment**:** The same classification criteria that apply to adults also apply to newly diagnosed child TB patients (table 10). Similarly, the treatment strategy

for TB patients is similar (table 11). The major differences for TB treatment in children compared with adults include:

(i) Drug dosages (which are described in detail in the national TB manual (table 5.3, page 49))

(ii) Fixed dose combination formulations (see annex 7, page 111 of national TB manual)[33]

- **HIV Care and Treatment:**
  - **ART eligibility:** For both adults and children, national ART eligibility criteria will be adhered to.

Table 12: ART initiation Criteria

Patient	Eligibility Criteria
**Adults**	
**Pregnant**	Life-long ART if:<br>- CD4<350<br>- WHO stage III/IV<br>Otherwise, ART for pregnancy and throughout breastfeeding.
**Non-pregnant**	Life-long ART if:<br>- CD4<350<br>- WHO stage III/IV
**Children**	
**≤12 months old**	All infants are eligible for ART
**>12 months**	- WHO clinical stage 3 or 4<br>- Advanced or severe immune suppression per WHO age-related guidelines (see below)

Table 13: Botswana MOH Definitions of Advanced Immunosupression in Children

	**<1 year**	**12-35 months**	**36-59 months**	**5-14 years**
**Mild**	30-35%	25-30%	20-25%	350-499
**Advanced**	25-29%	20-24%	15-19%	200-349
**Severe**	<25% or<br><1500 cells/ml	<20% or<br><750 cells/ml	<15% or<br><350	< 15% or<br><200

- **ART Regimens:** Appendix 7 shows the recommended first and second line regimens for Botswana as well as the implications of concurrent TB treatment and ART.

- **Pre-ART and ART Follow-up:** The recommended frequency of follow-up for patients enrolled in HIV care and treatment is listed below in table 14:

Table 14: Study Follow-up Procedures for Enrolled Patients

		Clinical Measurements
**Adults**		
**Pre-ART, CD4 >500**	**3 monthly***	Weight, WHO stage, CD4, TB screen
**Pre-ART, CD4 250-500**	**3 monthly**	Weight, WHO stage, CD4, TB screen
**ART**	**ART start**	Weight, WHO stage, CD4, , TB screen, ALT/AST if NVP-based regimen, Hb if AZT-based regimen, Hepatitis B screen, creatinine if TDF-based regimen
	**2 weeks**	Weight, WHO stage, TB screen, ALT/AST if NVP-based regimen, Hb if AZT-based regimen
	**1 month**	Weight, WHO stage, TB screen, ALT/AST if NVP-based regimen, Hb if AZT-based regimen
	**3 months**	Weight, WHO stage, TB screen, ALT/AST if NVP-based regimen, Hb if AZT-based regimen, Viral load, creatinine if TDF-based regimen
	**6months**	Weight, WHO stage, TB screen, ALT/AST**** if NVP-based regimen, Hb if AZT-based regimen, Viral load, CD4
	**Quarterly*****	Weight, WHO stage, TB screen, Viral load and CD4 6 monthly, creatinine if TDF-based regimen 6 monthly
**Children**		
**Pre-ART**	**3 monthly**	Weight, CD4 and CD4%, TB screen
**ART**	**Same as adults**	CD4% in addition to tests recommended for adults Viral load every three months for duration of ART

****Routine ALT/AST not required after 6 months but may be requested by the clinician depending on the clinical situation.
*****For those patients started on PI-based regimens, baseline and 12-monthly glucose (random or fasting) and total cholesterol/triglycerides are recommended.

- **Interviews at Follow-up Clinic Visits:**
  - At routine follow-up appointments, enrolled patients will be interviewed by the study nurse and certain data will be abstracted from their medical records (Appendix 8.1.).
  - In addition, routine TB screening procedures will be followed as have been described in depth above, unless the patient has already been diagnosed with TB.

- In addition, all blood, sputum or other test results will be returned and explained to the patient. If a test result is not yet available, the reason for the delay will be explained.
- Data from the TB records, located at TB clinics, of patients who start TB treatment will be abstracted at the end of TB treatment follow-up (Appendix 8.2.). If TB clinics are inaccessible (for example due to distance), obtaining the TB treatment outcome data via patient interview and review of patient-held cards will be attempted. If neither of these two options are successful, abstraction of relevant data from the electronic, patient-level database (ETR.net) in Gaborone, by one of the study investigators, will be performed.
- A study exit form (Appendix 8.3.) will be completed for all patients completing the study follow-up, or leaving the study for some other reason.

➢ **<u>Enhanced Retention Interventions to Reduce Loss to Follow-up</u>:**

- At study enrollment, the following contact details will be requested from the enrollee:
  (i) Personal contact telephone number and residential address.
  (ii) Family member/friend telephone number and residential address.
- If a patient is late for a clinic visit (misses a scheduled appointment by one day), the following procedures will be followed:
  (i) The patient will be telephoned (or the friend/family member if the patient is unreachable). If the patient is reached by telephone a new clinic visit will be scheduled in coordination with the clinic staff.
  (ii) If the patient cannot be reached by telephone and has missed the last clinic appointment by >7 days, active tracing procedures will be initiated. This will entail a study tracing team driving to the patient's residence and attempting to locate the patient.
  (iii) If the patient cannot be located through telephone or active tracing procedures, and the outcome of the patient is unknown, the patient will be considered lost to follow-up (LTFU). A patient will only be considered lost to follow-up if there have been at least 5 unsuccessful attempts to reach the

person by phone, 2 unsuccessful home visits and the patient has missed the last scheduled appointment by ≥3 months.

(iv) If the patient has died the date of death will be recorded as well as any data on possible cause of death.

(v) For all patients who became LTFU, a questionnaire collecting data on reasons for LTFU and events during the LTFU period will be administered (appendix 9).

- Patients will be followed through 6 months post enrollment or through the end of TB treatment, whichever time point is later.

➢ **Use of Botswana's National Mortality Database To Evaluate Outcomes of Prospectively Enrolled Patients who are LTFU**

To answer the second primary study question, obtaining an accurate estimate of mortality in the first six months of ART among patients in the prospective post-Xpert cohort, is needed. To obtain an accurate estimate of mortality, two procedures will be utilized: (1) tracing of patients considered LTFU (described above), and (2) use of patient-locating information to search Botswana's national mortality database. Botswana's national mortality database is located within the Department of Civil and National Registration in the Ministry of Labor and Home Affairs. Botswana law requires registration of all deaths that occur in Botswana, even among non-citizens, ideally within 30 days of the death (http://www.gov.bw/en/Ministries--Authorities/Ministries/Ministry-of-Labour--Home-Affairs-MLHA/Tools--Services/Services--Forms/Death-registration/).

To search the database, identifying information of patients who are LTFU would be needed, including "omang number", name, and date of birth. This information is collected on the patient locator questionnaire, which is maintained by study nurses for tracing purposes (Appendix 5.1).

Consent forms (Appendices 4.2.1., 4.2.2, 4.2.3., and 4.2.4) describe the need for investigators to collect identifying information for tracing purposes. This procedure for ascertaining patient outcomes has been used in previous prospective studies,[51] and is

being used for routine program evaluation purposes in Botswana.

- **Use of Botswana's National Mortality Database To Evaluate How Representative the Post-Xpert Prospective Cohort is**

There may be situations where the study nurse does not have time to enroll all patients eligible for the prospective post-Xpert cohort. In these circumstances, it would be important to know if mortality rates among those patients who are eligible for the post-Xpert prospective cohort, but were unfortunately not enrolled, are different from mortality rates of those patients enrolled in the post-Xpert prospective cohort.

To evaluate representativeness of the post-Xpert prospective cohort, we are requesting a waiver of informed consent to abstract data from the ART medical records of patients who were eligible for the post-Xpert prospective cohort, but who could not be enrolled due to human resource limitations. The following variables would be abstracted:

- First name, surname, national identification number (Omang number);
- Date of birth; age; sex;
- Village, town, and city of residence;
- Marital status; educational level; employment status; and
- Number of biological children
- Date of first clinic visit
- Date of ART initiation
- Outcome status at time of data abstraction (death, alive on ART, stopping ART, transfer out, or LTFU).
- Baseline CD4 count
- Baseline WHO stage

For patients who are considered LTFU at the time of data abstraction, the national mortality database would be used to assess vital status. We request a waiver of informed consent for medical record abstraction in accordance with 45CFR 46.116 (d). The waiver is appropriate because (1) the evaluation is retrospective in nature, and involves no more than minimal risk to human subjects; (2) the evaluation will not adversely affect the rights and welfare of the subjects; and, (3) the evaluation could not practicably be carried

out without the waiver.

4.4. Retrospective Cohort Procedures:

(a) <u>Study Design</u>: This study will be a retrospective cohort analysis of TB incidence and other treatment outcomes in the 24 months prior to prospective study enrollment.

(b) <u>Sources of Data</u>: Study data will come from four sources:

- Patient medical records (paper and electronic) at participating sites,
- Pharmacy records,
- Laboratory records,
- TB treatment registers if available,
- Interviews with one Health Care Worker at each site, and,
- A single interview with those patients enrolled retrospectively who are still alive and on therapy at the time of study start and consent to having an interview.

(c) <u>Patient Population</u>:

As described in section 3.3 (b), the following inclusion and exclusion criteria apply for **patients enrolled in the retrospective cohort:**

- **Inclusion criteria for retrospective cohorts**:
  - All ART patients who initiated ART in the 24 months prior to study start-up (which is considered the date of initiation of prospective enrollment).
  - **Exclusion criteria for retrospective cohorts**:
    - All prisoners

(d) <u>Selection of ART Records for Review at Each Site</u>:

- All adult and pediatric patients who initiated ART have charts with unique identification numbers, which are recorded on the front of the patient chart and in an electronic database.
- The electronic database also records the date of ART initiation alongside the clinic identity number.

- By searching the existing electronic database, study investigators will work with database managers to create lists of clinic identification numbers for all patients eligible for enrollment (all those who started ART at the site in the 24 months prior to study start-up) (Appendix 10).
- Once generated, the stored medical records will be located.
- Once located, the chart will be classified into one of the following categories:
  - Dead: Documented to have died
  - Transferred: Documented to have been transferred to another facility.
  - Stopped Care: Documented to have voluntarily stopped ART (rare).
  - Lost to follow-up (LTFU): Not documented to have died, been transferred, or stopped ART but has **not** attended the clinic in the preceding 90 days.
  - Presumed alive and on ART: Attended the facility in the preceding 90 days, and not documented to have died, been transferred, or stopped ART.
- **For patients who died, stopped ART, or were transferred**: The medical record will be abstracted (including the date of the documented outcome) and no interview will be pursued. For these patients, we are requesting a waiver of informed consent for data abstraction and use for this study. (See section 7.1 – Human subjects, for justification)
- **For patients LTFU**:
  - The medical record will be abstracted
  - The patient's final outcome status will be pursued through tracing procedures including: (i) up to 5 telephone calls; (ii) up to two home visits.
  - If the patient is returned to ART, the patient will be screened for TB using the four-symptom TB screen (cough, night sweats, loss of weight, and fever). For patients who screen negative for TB, the patients will continue with normal HIV care and treatment procedures.
  - For paitents returned to ART and who screen positive for TB, consent for the following will be requested: (1) interview and (2) use of routine data abstracted from their medical record. If consent for the interview is refused, we will request verbal consent for use of routine data abstracted from their medical record. If consent for both the interview and routine data use are refused, then the patient is excluded from the study. However, the patient will continue to receive standard of ART care.

- For patients who have died, been transferred or stopped ART, no interview will be pursued.
- For patients about whom no outcome status can be ascertained, an outcome status of LTFU will be allocated, and the date of LTFU will be the date after the most recent clinic appointment. For these patients, no interview will be pursued.

- **For patients presumed alive and on ART**:
  - The patient's medical record will be abstracted.
  - The patient's folder will be "flagged" to indicate the patient's potential eligibility for interview. This will be done by stapling a colored marker to the chart's outer folder.
  - The date of the next scheduled appointment will be recorded by the study nurse.
  - When the patient attends the routine appointment, the attending clinician will ensure the 4-symptom TB screen is completed. This is done by clinic staff, usually clinic nurses.
  - The clinician will administer the four symptom TB screen, in accordance with Botswana national guidelines.
  - If the patient screens negative, the patient will continue with normal care and treatment procedures and the patient's medical record will be abstracted. We are requesting a waiver of informed consent for data abstraction for these patients. (See section 7.1 – Human subjects, for justification)
  - If the patient screens positive for TB, the following will be requested: (1) interview and (2) use of routine data abstracted from their medical record. If consent for the interview is refused, we will request verbal consent for use of routine data abstracted from their medical record. If consent for both the interview and routine data use are refused, then the patient is excluded from the study. However, the patient will continue to receive standard of ART care.
  - If consent for an interview is granted, the patient will be interviewed.

(e) Managing the Problem of Missing Charts:

- In the event that a selected, eligible medical record is not found at its expected storage location, the study team will make every effort to locate the selected record. Possible locations of a missing medical record include: (1) the data entry room; (2) in a

consultation room; (3) at the pharmacy; (4) at the social worker's office where it is being used for tracing purposes, but there may be other locations that need to be searched.

- Records of numbers of missing charts and charts removed for patient tracing will be kept and form part of the report generated by this evaluation. In addition, using a data abstraction questionnaire, we will abstract as much information on patients with missing charts from the electronic database as is possible. In a sub-analysis, we will attempt to define if charts are missing at random or if a pattern of "missingness" can be detected. This information will be used to define if the problem of missing charts biases our findings in any way.

(f) Data abstraction:

- All HIV care and treatment facilities included in the study have an electronic medical register [the Patient Integrated Management System, version 2 (PIMS2)]. Appendix 11 shows the data we need from patient files. Most of these data will potentially be collected from centrally maintained electronic medical records. However, as described above, study nurses will review facility-based paper records to ascertain the patient's final outcome status (alive on ART, LTFU, stopped ART, transferred out, or dead). In addition, study nurses will review the paper medical records for the key variables of: (1) TB incidence during ART follow-up, and, (2) hospitalization during follow-up.
- Study nurses will perform the data abstraction at times when they are not busy prospectively enrolling new HIV clinic enrollees.
- Study nurses will have experience in clinical medicine.
- Training in abstraction will occur before study start at a training facility in Gaborone. During training, the importance of maintaining confidentiality of the patients whose charts are reviewed will be emphasized. Although no names will be abstracted, the abstractors will see names on charts reviewed. The study nurses will be asked to sign a form stating that they will not disclose any information they read in the charts.
- As part of the training for abstraction, before the study begins, procedures for medical chart review will be piloted at one to three health facilities to ensure that the instruments are appropriate and that all team members are given consistent directions. The

instruments will be revised, as necessary, before the full data collection process starts (Appendix 11).

(g) <u>Supervision of Data Abstraction</u>:

- To assess quality of the electronic medical records, the first 5% of medical records selected for inclusion in the retrospective study, will be fully abstracted from the paper medical records (using appendix 11) and compared with the electronic data. Investigators will assess concordance between electronic and paper medical record data variables. Paper medical records will be considered the gold standard. The key variables of: (1) date of birth, (2) date of ART start, (3) CD4 count at ART start, (4) and WHO stage at ART start, will be used to assess reliability of the electronic data. If there is a ≥95% concordance between electronic and paper records, electronic data will be used to complete all data fields except: (1) TB incidence during ART follow-up, (2) hospitalization during follow-up, and (3) final outcome status at the time of abstraction. These three outcome variables will be abstracted from paper medical records by the study nurse. If there is <95% concordance between electronic and paper medical records, all data will be abstracted from paper medical records.
- To ensure quality of data abstraction from paper medical records, all data abstraction forms will be reviewed and 5% of abstracted questionnaires will be randomly selected for re-abstraction by field team supervisors.
- Charts for re-abstraction will be selected by dividing the total charts reviewed by 20 to get an interval "N". A random whole number between 0 and "N" will be randomly selected. This random number will serve as the first chart to be re-abstracted. Every "Nth" chart after this random number will also be re-abstracted until 5% of charts have been re-abstracted.
- Once the charts have been re-abstracted, any discrepancies between data initially abstracted and data that were re-abstracted will be discussed and the option which is unanimously agreed to be correct will be selected. If there is no agreement between team facilitators and data abstractors, a pre-selected investigator will be contacted to gain advice on how to proceed. Lessons learnt from the re-abstraction process will be shared with all study nurses.

- Feedback will be given to the clinical staff at the end of the session based on the observations of the abstractors. Feedback will focus on the importance of monitoring and evaluation of ART for quality promotion.

(h) <u>Patient Interviews</u>:

- As described above, study staff will be informed that every patient with a flagged folder should be referred to the study nurse for consent procedures. In addition, the study nurse will have a list of dates and clinic ID numbers, indicating when eligible patients should be presenting to the clinic.
- Study nurses will request consent (and where necessary, assent) from eligible patients (i.e. those patients still alive and on ART who screen positive for TB) ,and where necessary, their guardians, for participation in a single interview.
- If consent for the interview is granted, the interview will be conducted (Appendix 12).
- Some patients, who were thought to be alive and on ART, will be re-classified as dead, or LTFU if they do not return for the scheduled appointment. Tracing of patients who are LTFU will be attempted as described earlier. Data from the medical records of patients who have died or been LTFU will be abstracted.
- Study exit forms will be completed for all patients enrolled in the cross sectional interviews. Exit forms would be filled either after completing study follow-up (which would occur if the patient screened positive for TB and received results for sputum samples), or if the patient left the study for some other reason (Appendix 8.3.).

(i) <u>Health Care Worker Interviews</u>:

- An interview with the health care worker who is considered the most knowledgeable about the HIV clinic function will be requested (Appendix 13).
- If consent is granted, site-level characteristics will be documented.
- Only one site-level interview need be completed for each facility.

(j) <u>Abstraction of TB Treatment Outcome Data</u>:

- Retrospective TB treatment outcome data will be abstracted from the electronic

Tuberculosis Register (ETR.net) in Gaborone Botswana, ensuring that most of the data captured for prospective patients (Appendix 8.2), is captured for the retrospective comparison group.

- TB treatment outcome data for all TB-HIV co-infected patients, who enrolled in one of the study clinics in the 24 months prior to study start, and were diagnosed with TB in the first year after HIV clinic enrollment, will be abstracted and analyzed to assess proportions of patients in the pre-Xpert period with documented treatment completion or cure.
- This abstraction of ETR.net data will be performed by one of the investigators in coordination with the Ministry of Health.
- Matching of a patients ART medical record with his/her TB record will be achieved via one or a combination of identifiers. We will abstract the “Omang” number, a number which identifies the patient as a Botswana citizen (and therefore eligible for government health care) from both ART records and ETR.net to allow for this matching.
- ETR.net was developed using the Microsoft.NET platform (VB.NET 2.0), and is a successor to the TBREG software (Epi- Info6) developed by The BOTUSA Project (CDC-Botswana).
- ETR.net captures patient-based information at the district level directly from paper TB registers. Therefore, patient-level observations, for our cohort of interest, are available at the central TB program evaluation office in Gaborone.
- All variables listed in Appendix 8.2 are available in the ETR.net database (http://www.etrnet.info/Default.aspx).

(k) Use of Botswana’s National Mortality Database

To answer the second primary study question, obtaining an accurate estimate of mortality in the first six months of ART among patients enrolled on ART in the 24 months before study start, is needed. To obtain an accurate estimate of mortality, two procedures will be utilized: (1) tracing of patients considered LTFU (described above), and (2) use of patient-locating information to search Botswana’s national mortality database. Botswana’s national mortality database is located within the Department of Civil and National Registration in the Ministry of Labor and Home Affairs. Botswana law requires

registration of all deaths that occur in Botswana, even among non-citizens, ideally within 30 days of the death (http://www.gov.bw/en/Ministries--Authorities/Ministries/Ministry-of-Labour--Home-Affairs-MLHA/Tools--Services/Services--Forms/Death-registration/).

To search the database, patient identifying information routinely recorded on the ART medical records of patients who are LTFU is needed, including "omang number", name, and date of birth.

Because patients are not routinely attending the clinic (i.e. are considered LTFU), it is not possible to request their consent for use of identifying information to search the Botswana national mortality register. We request a waiver of informed consent for medical record abstraction in accordance with 45CFR 46.116 (d). The waiver is appropriate because (1) the evaluation is retrospective in nature, and involves no more than minimal risk to human subjects; (2) the evaluation will not adversely affect the rights and welfare of the subjects; and, (3) the evaluation could not practicably be carried out without the waiver. This procedure for ascertaining patient outcomes has been used in previous studies,[51] and is being used for routine program evaluation purposes in Botswana.

To ensure that retrospective cohort-eligible patients are only screened for TB once by the study team, study nurses would use Appendix 10 (the retrospective cohort study register) or a similar register to keep track of which eligible patients have been screened and which patients still require screening.

4.5. Summary of Procedures that go Beyond Standard of Care for Prospective and Retrospective Cohorts:

Prospective Cohorts:

1. Prospective cohort patients will be interviewed after each routinely scheduled clinic visit.
2. Certain data, described in detail in the data collection forms, will be abstracted from the medical records of prospective cohort patients.
3. Prospective cohort patients who screen positive for TB using the MOH-recommended TB screen, will provide 4 sputa:

- Day 1: 2 on-the-spot sputa
- Day 2: 1 morning sputum and one on-the-spot sputum.

  MOH guidelines recommend only 2 sputa be collected:
- Day 1: One on-the-spot sputum
- Day 2: One morning sputum

4. All prospective patients agreeing to be part of the study, who screen positive for TB, will have sputum sent for: (1) culture; and (2) if culture-positive, line probe assay testing and drug resistance testing. In routine practice, only HIV-infected TB suspects who are smear-negative on routine microscopy, or who have a history suggestive of drug-resistant TB, would have sputum sent for culture. Line probe assays and drug susceptibility testing is usually reserved for TB suspects with a history suggestive of possible TB drug resistance. (As with all other HIV-infected patients attending the site, Xpert will be used for sptum testing if it is in place at the time of the cross-sectional interview).
5. At the enrollment visit only for asymptomatic HIV-infected children (<=12 years old) 2 sputa will be collected for TB culture to assess the sensitivity of the MOH algorithm in detecting culture positive TB disease, if they are able to produce sputa voluntarily.

Retrospective Cohorts:

1. Retrospective cohort patients who are alive and on ART at the time of study initiation, and who screen positive for TB, will be asked for consent for one interview. The interview will take place after the next scheduled visit for the patient once the study has started.
2. Patients who screen positive for TB and consent to the cross-sectional interview, will provide 4 rather than 2 sputa (as described above).
3. Sputa will be sent for (1) culture; and (2) if culture-positive, line probe assay testing and drug resistance testing. (As with all other HIV-infected patients attending the site, Xpert will be used for sptum testing if it is in place at the time of the cross-sectional interview).
4. Routinely collected data will be abstracted from the paper medical records.

Note that tracing procedures are not study specific as they are recommended by the Ministry of Health for routine care.

4.6. Summary of Variables to be Collected:

All variables below will be collected for prospective cohorts and where possible for the retrospective cohort. Variables collected for the retrospective cohort are limited to some extent by the variables already present on the MOH-approved standard ART medical records. In the table below, data collection for the prospective cohort will occur through interview and in certain circumstances by review of data recorded by the attending physician. Details of which data source will provide information for each variable are included with the questionnaires. Similarly, for the retrospective cohort, two types of data sources will provide the necessary information: (1) the patient's medical records (including pharmacy, laboratory and patient-held medical records); and, (2) interview with the patient (if the patient is alive and in care and consents to the interview).

Table 15: Demographic and Clinical Variables to be Collected for Prospective and Retrospective Cohorts

Variables	Frequency of collection	Prospective	Retrospective
**Demographic Variables**			
Date of birth	BL	✓	✓ *
Age at enrollment into HIV care	BL	✓	✓ *
Sex	BL	✓	✓ *
Marital status of the patient	BL	✓	✓ *
Educational level reached at the time of enrollment into HIV care and number of years of education	BL	✓	✓ *
Employment status	BL + F/U	✓	✓(BL only)*
Housing type	BL	✓	✓
Availability of running water	BL	✓	✓
Number of people in household	BL	✓	✓
Number of biological children at the time of enrollment into HIV Care and their ages	BL	✓	✓ *
Distance from the clinic (estimated number of kilometers and amount of time to reach the clinic)*	BL	✓	✓
Cost of transport to the clinic*	BL	✓	✓
Whether the patient has disclosed his/her status to family/friend (adults only)*	BL + F/U	✓	✓ *
**Past Medical History**			

History of previous TB [date of diagnosis, type of TB (table 10), duration of TB treatment, outcome of TB treatment* (appendix 3)]	BL	✓	✓ *
History of previous treatment for opportunistic infections (OI) other than TB, type of OI, and date of OI occurrence.	BL	✓	✓ *
Previous exposure to ARVs and type of ARVs.	BL	✓	✓ *
Date of HIV diagnosis (and type of HIV)	BL	✓	✓ *
Current medication list	BL	✓	✓ *
Alcohol history	BL	✓	✓
Smoking history	BL	✓	✓
**TB Screening and Diagnosis Information**			
Was the patient administered the appropriate TB screening algorithm and which of the screening questions were positive and which negative?	BL + F/U	✓	✓ *
If screened positive which diagnostic tests were ordered and what were the results?	BL + F/U	✓	✓ *
Date of TB diagnosis and type of TB (table 10)?	BL + F/U	✓	✓ *
Type of TB treatment administered?	BL + F/U	✓	✓ *
Was the patient enrolled in direct observed therapy?	BL + F/U	✓	✓ *
TB contact information (within + outside household contacts)	BL + F/U	✓	✓ *
**Clinical Exposure Variables**			
Weight	BL + F/U	✓ *	✓ *
Height	BL + F/U	✓ *	✓ *
Blood pressure	BL + F/U	✓	
Temperature	BL + F/U	✓	
Respiratory rate	BL + F/U	✓	
Pulse rate	BL + F/U	✓	
WHO stage	BL + F/U	✓	✓ *
Whether the patient continues to take TB treatment, date of TB treatment start, type of TB, and TB treatment outcome (see table 10 and appendix 8.2)	BL + F/U	✓	✓ *
Whether taking isoniazid preventive therapy (IPT) and date of IPT start	BL + F/U	✓ *	✓ *
Whether taking co-trimoxazole (CTX) and date of CTX start	BL + F/U	✓ *	✓ *
If not taking CTX/IPT, give the reason*	BL + F/U	✓ *	✓ *
CD4 count (and CD4% for children)	BL + F/U	✓ *	✓ *
Viral load if available	BL + F/U	✓ *	✓ *
Hemoglobin (Hb)	BL + F/U	✓ *	✓ *
ALT and AST if available	BL + F/U	✓ *	✓ *
Creatinine clearance if available	BL + F/U	✓ *	✓ *
Whether considered ART-eligible by the attending clinician	BL + F/U	✓ *	✓ *
Date of ART start	BL + F/U	✓ *	✓ *
**Outcome Variables**			
**Alive and in care:**			

• On time for clinic appointment‡	F/U	✓	✓ *
• Was late for clinic appointment (# days)	F/U	✓	✓*
• Returned to clinic after being considered LTFU (>90 days late for last appointment)	F/U	✓	✓ *
**Lost to follow-up and untraceable:**			✓ *
•>90 days late for a clinic appointment and not documented to have died, been transferred or dropped out of the study)	Final status	✓	✓ *
**Dead:**			✓ *
•include date of death and clinician-suspected cause	Final status	✓	✓ *
**Transferred:**			✓ *
•to another facility (and date of transfer)	Final status	✓	✓ *
**Voluntarily stopped care:**			✓ *
•Refused follow-up HIV care and treatment and therefore also refused study participation (include date of stopping care)	Final status	✓	✓ *
**Voluntarily stopped study follow-up:**			✓ *
•Refused follow-up HIV care and treatment and therefore also refused study participation (include date of stopping follow-up)	Final status	✓	✓ *

*Variables to be abstracted from the patient's medical records

(a) Variables Specific for Children:

- Certain variables specific to pediatric care will be collected. These include:
  - Whether the child is aware of his/her own HIV status;
  - Birth weight;
  - APGAR score;
  - Vaccination status;
  - Whether the child's mother received PMTCT antiretrovirals (ARVs) and type of ARVs

(b) Laboratory Specific Variables:

- Each sputum (or other TB diagnostic sample such as a biopsy) will be tracked from the time of collection through laboratory analysis with its own study form (Appendix 5.2.)
- Variables to be collected for each specimen include:

Table 16: Variables Collected for Each TB Diagnostic Specimen

**Patient Information**	
Study Identification Number	

Clinic Identification Number		
Clinic name		
Sex		
Date of birth		
**Type of Specimen**		
Spot sputum		
Morning sputum		
Lymph node biopsy (specify site and method of biopsy)		
Other biopsy (specify site and method of biopsy)		
Fluid aspirate (specify type of fluid and source)		
**Method of Sputum Collection (if specimen is sputum)**		
Not induced		
Induced		
Gastric aspirate		
**Date and time of sputum collection**		
Date (dd/mm/yyyy)		
Time (e.g. 14h00)		
**Sputum Transport**		
	**From collection site (clinic) to peripheral TB diagnostic Lab**	**From Peripheral TB diagnostic Lab to Reference Lab**
Name of person transporting specimen		
Date (dd/mm/yyyy)		
Time of departure (e.g. 14h00)		
Name of person receiving specimen		
Date (dd/mm/yyyy)		
Time of receiving specimen (e.g. 14h00)		
**Tests performed at routine TB diagnostic lab**		
	**Smear Microscopy**	**XPERT**
Name of operator		
Date		
Time		
Result		
Date and time result returned to clinic staff		
Date and time result returned to patient		
**Tests performed at Central Lab**		
	**Flourochrome Smear Microscopy**	
Name of microscopist		
Date		

Time		
Result		
Date and time result returned to clinic staff		
Date and time result returned to patient		
**Culture-dependent Tests performed at Central Lab**		
	**Liquid Culture**	**Drug Susceptibility Testing**
Name of operator		
Date of culture start		
Time of culture start		
Result of culture		
Date and time of culture result at central lab		
Date and time result returned to clinic staff		
Date and time result returned to patient		
**Final Outcome of Sample**		
Stored/Disposed		
Date of storage/disposal		
Name of person responsible		

(c) <u>User Acceptance Survey for Xpert Device Operators</u>

- To allow estimation of the level of GeneXpert user satisfaction following rollout of GeneXpert devices to support study clinics, study investigators with laboratory training will administer a user acceptance survey 6-monthly, after GeneXpert rollout. The user acceptance survey is attached as appendix 5.4. The protocol for conducting the user-acceptance survey is as follows:
  - **Elligibility for Survey**: All operators of the Xpert device will be asked to complete the user acceptance survey twice each calendar year for the duration of the study.
  - **Sample Size**: There is no need for a desired precision or power. The study is descriptive. All operators will be asked consent for interview as described in appendix 5.4. We estimate that 3-4 people will be trained to operate each of 13 devices during the course of the study, for survey size of 39-52 persons annually.
  - **Procedures:** Every 6 months, each Xpert location will be visited by one of the study investigators at a time comvenient for the Xpert operators. The Xpert operators will be asked for written consent to participate in the interview. The operators will be reassured that all responses are confidential. Signed consent forms will be kept

separate from the stored questionnaires. Other measures to preserve the confidentiality of the Xpert operator are described in Appendix 5.4 and include:

- The operator's name will not appear on the interview questionnaires and the information collected during individual interviews will not be shared directly with other clinic or lab staff or your supervisor.
- Only study investigators, who perform the interviews, will have access to a computerized table linking an operators name to a code (a 3 digit number between 1 and 999). Only the operator's code will be entered onto the user acceptance questionnaire. This code is necessary to allow the investigators to link 6-monthly user acceptance surveys longitudinally. Linking surveys longitudinally is important to help investigators learn if user-acceptability improves, declines or stays the same over time.
- The information gathered in the interviews will be kept in a locked and secure place for up to five years and during that time only the study staff from Botswana Ministry of Health, and CDC will have access to this information.
- Additionally, members of the Botswana Committee on Research Ethics, and U.S. regulatory agencies like the Office for Human Research Protections (OHRP) may look at the research records.
- After five years, the hard copies of the study files will be destroyed.
- All study staff, including investigators, will be trained on confidentiality and data security issues and will be required to sign confidentiality agreements.
- By ensuring confidentiality as far as is reasonably possible, risk to the operator's job security will be minimized as far as the investigators are able.

(d) Temperature Logger

- To monitor the temperature of the location where GeneXpert devices and kits will be located as well as the electricity supply, an environmental monitoring log will be placed in the same room as GeneXpert devices and kits. Examples of environmental monitoring logs which may be used for the study are found at: http://www.chartlogger.com/Dickson-Temperature-and-Humidity-Data-Logger.html

(e) Monitoring Workspace Cleanness and Clutter

- To monitor workspace cleanness and clutter, study supervisors will visit the location of Smear microscopy and GeneXpert use on a quarterly basis with a standardized site assessment form (Appendix 5.4). In addition, the supervisors will take photographs of the laboratory or clinic space being used for smear microscopy or GeneXpert during quarterly visits, being sure not to include any identifiable persons, or laboratory specimens in the photographs.

4.7. Quality Assurance

(a) Laboratory

- Good laboratory practices will be followed for all laboratory testing. Standardized operating procedures will be used by all sites involved in the study. Procedures will be in place for performing and documenting the quality of a specimen, including storage and transport conditions, monitoring of equipment temperatures and function indicators, control of cross-contamination during culture and molecular testing, control for acceptable isolation and contamination rates, control for AFB smear accuracy and staff competency, and control for accuracy of TB identification. In addition to general Quality Control procedures, specific procedures will include enrollment in quality assurance (QA) programs for Xpert MTB/RIF, AFB smear, and TB culture.

- A comprehensive QA program is being devised to monitor Xpert in the lab and at the POC. It will be included in the laboratory manual developed for this study. Briefly, QA will include the following:
  - ➢ SOPs will be provided and customized for each testing site.
  - ➢ Training and competency assessments will be performed for all staff performing the test
  - ➢ Equipment error logs will be maintained and monitored at least monthly in each testing site. (The GeneXpert will record and display error codes specific to common equipment failures such as power outages.)

- Similar to TB culture quality assurance and, Xpert MTB/RIF performance indicators will be monitored monthly. Indicators will include the following:

The % of Xpert results which are invalid
The % of AFB-smear positive patients who are Xpert-TB positive for TB
The % of AFB-smear negative patients who are Xpert-TB positive for TB
The % of Xpert-TB positive patients among patients who were smear-negative (incremental yield of Xpert over smear)
The % of patients who are Xpert-TB positive for TB who are also Xpert-TB RIF resistant
The % of culture-positive patients who are Xpert-TB positive for TB
The % of culture-negative subjects that are Xpert-TB positive for TB
The % of patients who are Xpert-TB RIF resistant who are also RIF resistant by conventional DST
The % of patients who are Xpert-TB RIF susceptible who are also RIF susceptible by conventional DST
The % of patients who are Xpert-TB RIF resistant who are also multidrug resistant by conventional DST

- Building upon the existing smear microscopy external QA program in Botswana, on-site supervisory visits and proficiency panel testing will be performed quarterly
- Environmental temperatures in the testing facilities and cartridge storage facilities will be monitored with temperature loggers (as described above)
- All data routinely captured by the GeneXpert device software (e.g. name of operator, date, time, result) will be captured. If an error occurs, operators will be trained to ascertain what is the most likely cause (e.g. air in the specimen can cause an error). Possible causes of Xpert errors will be captured by GeneXpert operators.

- At the Botswana Ministry of Health reference laboratory and the CDC research laboratory, additional storage facilities consisting of refrigerators (4°C), freezers (-20 ^{o}C) and ultra freezers (-80 ^{o}C) will be available for the short term and long term storage of specimens. An effective external quality assessment (EQA) to ensure integrity of the specimens will be in place at the reference laboratory. A reliable electrical supply and backup will be necessary at the reference laboratory to ensure constant electricity for proper functioning of the TB culture devices (BACTEC 960) and for maintaining stored specimens. Each stored specimen will have a unique identifier which is linked to the study participant's study number. The specimens will be stored, through collaborative efforts between CDC Botswana and the Botswana Ministry of Health for a minimum of two years. When the TB/HIV Research Division at CDC Botswana closes, the government, as co-Investigators and co-owners, will continue to maintain the specimens.

- At the clinic laboratories, the national guidelines for sputum smear microscopy will be adhered to. These are coordinated by the national TB program and generally describe periodic inspection visits, retesting of a sample of smears and periodic testing of external proficiency panels. Standard quality assurance practices will be followed for the other laboratory tests (hematology, immunophenotyping, and serum chemistry).

- Regarding chest radiography, we will work to assure that the radiography equipment is functioning properly. The study teams will work to assure that each participating radiology clinic has an up-to-date certification. Radiograms will be read twice, initially by the physician associated with the radiography center. A second reading will be conducted by study physicians who will be trained in the standardized reading of chest radiograms. These physicians will also be responsible for completing the chest radiography results form (Appendix 14). Any discrepancies in overall interpretation will be reviewed between the two readers to agree upon a consensus interpretation.

(b) Clinical care

- The Ministry of Health (MOH) has standard quality assurance programs intended to monitor and maintain quality of the HIV care and treatment program and the TB program. These programs will be continued.
- Through constant field supervision, the study team investigators will work with regulating bodies to identify potential program weaknesses to ensure a high standard of care.

(c) Adherence to study procedures

- Throughout the study, project managers will perform routine, random site visits to check on adherence to study procedures.
- Where study procedures are violated (e.g. no X-ray is performed after a sputum sample for a TB suspect is assessed as negative with smear microscopy) the event will be documented and corrected where possible.

- Data entry checks will serve to identify missing crucial data, inconsistencies in data collection (e.g. date of death before date of HIV clinic enrollment) or errors in data collection (e.g. weight measurements >1,000 kilograms).

5. Data management

5.1. Data flow

(a) As described above, data will be collected from various sources (e.g., patient interviews, HIV clinic records, TB treatment cards, and laboratory log books).

(b) All *clinical* information for enrolled patients will be collected by the study nurse onto paper-based questionnaires that will be verified for accuracy and completeness by site supervisors.

(c) All results of *laboratory* tests will be entered by laboratory personnel.

(d) All questionnaires, once complete, will initially be stored in locked, steel cabinets at the health facility or laboratory.

(e) Questionnaire transport from the health facilities or laboratories will occur on a weekly basis.

(f) During transfer, all questionnaires will be locked in a brief case, or securely fastened within a box with adhesive wrappings.

(g) All complete study forms will be transported to a central location in Gaborone, CDC-Botswana, for data-entry.

(h) Upon reaching the CDC data entry location, the questionnaires will be locked in a steel cabinet until the time of data entry.

5.2. Data entry

(a) Data will be double-entered using Census and Survey Processing Systems (CSPro) software.

(b) The software will:

- Only accept variables within reasonable pre-defined ranges (e.g. CD4 0-2,000/uL)
- Detect and prevent entry of inconsistencies (e.g. date of death before date of HIV clinic enrollment, or, a sputum result from a laboratory form which does not match the sputum result recorded on the questionnaires completed by the study nurse (appendices 5, 11, or 12)

- Detect missing data fields and prevent omission of crucial data (e.g. missing date of HIV clinic enrollment).

(c) Print-outs of comparisons of data which have been double data-entered will be used to document the double data entry and data correction processes. By randomly checking 10% of all variables documented to have been corrected in the final clean database, the data entry manager will be able to confirm that true double data entry and cleaning occurred.

(d) Data from the various questionnaires for a single patient will be linked by the patient's unique study number.

(e) All data will be stored on desktop computers with password-protected login screens.

(f) Each day, all data entered will be backed up on the data manger's desktop computer and in a secure location on the CDC Botswana shared drive.

(g) Only the data manager and the study investigators will have access to accumulating data across all sites.

5.3. Storage of paper questionnaires

(a) Paper questionnaires will be destroyed after all data has been electronically entered (double-entered), the study database is cleaned and investigators have verified that the database is finalized. Disposal of printed materials will be through a method which renders the material unreadable; a local vendor able to provide confidentialshredding, including cross-shredding, will be selected. Patient consent forms will be securely stored at CDC Botswana for 6 years after study completion.

5.4. Data transfer

Electronic data may need to be transferred from CDC Botswana investigators to MOH investigators or CDC Atlanta investigators or vice versa. Data will never be transferred via email. Instead, investigators will use PSFTP data sharing mechanisms. This program allows upload and download of files from investigator personal desktops with MS Windows operating software to remote servers located at CDC Atlanta. Only investigators will be aware of the instructions for accessing the PFSTP site including the passwords needed to access the server.

When the TB/HIV Research Division closes at CDC Botswana, the full study dataset will be securely transferred to CDC headquarters. Study datasets without PII will also be made available for public access, through an approved platform such as data.cdc.gov.

6. Analysis Plan

6.1. Study Flow Diagram

A study flow diagram will illustrate for each of the following cohorts: (1) Retrospective; (2) Prospective pre-Xpert; and (3) Prospective post-Xpert cohorts, the following:

(a) Number of patients eligible for enrollment

(b) Number of patients who consented to study enrollment and number of patients who declined consent to study enrollment

(c) Number of patients who: (1) were lost to follow-up; (2) stopped HIV care and treatment; (3) preferred to drop out of the study; (4) or were transferred to a non-study location for routine HIV care and treatment before the end of protocol-defined study follow-up. All enrolled patients not meeting these criteria for these endpoints will either be alive in HIV care and treatment at the end of study follow-up, or will have died during study follow-up. Using available data, analyses to aide assessment of possible selection bias due to enrollment refusal or cohort drop out will be conducted.

6.2. Baseline Characteristics

Key clinical and demographic characteristics of each of the three cohorts will be summarized using means, medians or frequencies. Continuous variables will be compared using t-tests if normally distributed, and Wilcoxon rank sum tests (Mann-Whitney tests) if not normally distributed. Categorical variables will be compared using chi-square tests.

6.3. Primary Study Questions

For the first primary study question:

(a) We will employ a mixed-model approach identical to that presented in Hussey and Hughes (2007).[45] The model will be fit in SAS PROC GLIMMIX, a procedure in SAS developed to fit generalized linear mixed models. The dependent variable is dichotomous, indicating whether the diagnostic algorithm detected TB or not (only those with true TB detected by

liquid culture will be included in the sensitivity analysis). A fixed effect for time will be included in the analysis to adjust for any time trends that might bias the pre- post comparison if not adjusted for. A fixed effect for intervention condition (0 before Xpert implementation, 1 afterward) will also be included and is the test of the intervention effect. A random effect for clinic will be included to adjust for between-clinic variation. The intervention effect will be judged significant at $p<0.05$ with a two-tailed test.

(b) **Analysis and Interpretation of All-Cause Mortality Rates**: As described earlier, through reductions in the burden of undiagnosed TB, we would expect TB-associated, and therefore all-cause, mortality among ART patients during the first 6 months of therapy to decrease post-Xpert package rollout.

However, we will need to explore the validity of the all-cause mortality data. Firstly, we need to assess the validity of pre-Xpert mortality estimates during the first 6 months of ART. By actively tracing all retrospectively enrolled patients, who are assessed as being lost to follow-up (LTFU) at the time of study start, we will determine true outcomes for those patients we are able to trace. In a previous study in Botswana, 58.8% of patients initially thought to be LTFU, were found to be dead after tracing, and this increased true mortality estimates.[40] Secondly, we will need to consider the impact of TB culture on all-cause mortality post-Xpert rollout. We will not be able to accurately describe the relative contribution of TB culture to any observed reductions in all-cause mortality post-Xpert

(c) **Analysis and Interpretation of TB incidence Rates:** We will estimate TB incidence in the first 6 months of ART, among patients who are considered TB-free at ART start, before and after Xpert package rollout, and compare these estimates using models that take into account the censoring of observations and the between-clinic variation. We will perform the same analysis to estimate if there is a significant difference in all-cause mortality in the first 6 months of ART pre- and post-Xpert package implementation. Because CD4 eligibility criteria for ART initiation are changing from <250/µL to <350/µL, we will need to account for patient CD4 count at ART initiation, in adjusted Cox proportional hazards regression analysis of comparing pre- versus post- Xpert package TB incidence and mortality.

Analysis and Interpretation of TB Incidence Rates: When discussing observed pre- and post-Xpert package TB incidence rates during the first 6 months of ART, we will need to consider several factors and limitations. Firstly, we will need to consider the role that TB culture, which will be performed for all prospectively enrolled TB suspects, played in identifying true active TB patients. When analyzing post-Xpert TB incidence rates, we will explore how TB incidence rates change if Xpert-negative, culture-positive TB cases are *not* considered incident TB cases. This analysis will help investigators to explore the role of TB culture in observed, post-Xpert package, TB incidence rates. Secondly, we will need to discuss the accuracy of the retrospectively accumulated data on TB incidence. Several studies have used retrospective data to assess TB incidence rates during ART and therefore, our approach has a precedent.[10, 13] However, retrospective analysis of incident TB is dependent on accurate documentation by attending clinicians. In Botswana, the routine electronic ART patient records do routinely undergo quality control checks by the Ministry of Health. This process is supported by the Botswana Harvard Partnership (BHP), a CDC implementing partner. The routine quality control exercises completed by the monitoring and evaluation unit improve the likelihood that observed TB incidence rates from retrospective data, are valid. To further explore the validity of TB incidence rates in the retrospective cohort, we will compare interview-reported TB incidence rates with documented TB incidence rates among patients who are selected for retrospective cohort enrolment, are alive and on ART at the time of study start, and consent to a cross-sectional interview. Another way to explore the accuracy of retrospective TB incidence data is to compare retrospective ART clinical records with retrospective TB records (in ETR.net) using the “omang” number to match records. If an ART patient’s “omang” number is in the ETR.net database because the patient started TB treatment, this is evidence of incident TB. If Omang numbers are missing, probabilistic matching algorithms to link ART with TB medical records, using variables such as date of birth and sex.

After assessing the validity of observed TB incidence rates pre- and post-Xpert, careful interpretation of any observed differences in TB incidence will be needed. For example, decreases in post-Xpert package TB-incidence during the first 6 months of ART among those

diagnosed as TB-free at ART start, might support the interpretation that the Xpert package correctly diagnosed more active TB patients prior to ART start by: (1) better screening and identification of TB suspects, and (2) better diagnosis of TB prior to ART start among identified TB suspects. However, it is possible that we will observe no change or increases in TB incidence during the first 6 months of ART, post-Xpert package implementation. This might support the interpretation that, although the Xpert package probably reduced the prevalence of undiagnosed TB at ART start, improved diagnosis of incident TB among patients with active sub-clinical disease at ART start or new active TB disease acquired after ART start, resulted in unchanged or increased TB incidence post-Xpert.

Further explanation of how retrospective and prospective cohorts can be compared for key variables of interest:

Outcome of interest	Data Field	Retrospective Cohort	Prospective Cohorts
**TB Incidence**	1. **Medical record**	**To be completed at each facility visit: "Is the patient taking TB treatment and date of TB treatment start"**	**To be completed at each facility visit: "Is the patient taking TB treatment and date of TB treatment start"**
	2. *Questionnaire*	*This information is abstracted onto appendix 11.1, Q #49*	*Information on TB diagnosis is collected on appendix 8.1, Q#41*
	3. *Report*	*Incidence rate of TB in the first 6 months of ART (events/100 person years)*	*Incidence rate of TB in the first 6 months of ART (events/100 person years)*
**All-cause Mortality**	1. **Medical record**	**To be completed at each scheduled facility visit: "What is the patient's vital status:**<br>**- Alive on ART**<br>**- Late for scheduled appointment**<br>**- Dead**<br>**- Stopped ART**<br>**- Transferred out"**	**To be completed at each scheduled facility visit: "What is the patient's vital status:**<br>**- Alive on ART**<br>**- Late for scheduled appointment**<br>**- Dead**<br>**- Stopped ART**<br>**- Transferred out"**
	2. *Questionnaire*	*This information is abstracted onto appendix 11.1, Q #42)*	*This information is collected with appendix 8.3, Q #8*
	3. *Report*	*Incidence rate of death in the first 6 months of ART (events/100 person years)**	*Incidence rate of death in the first 6 months of ART (events/100 person years)**
**TB Screening at ART start**	1. **Medical record**	**To be completed at each scheduled facility visit: Whether the patient was screened for TB, date of screening, and screening questions used.**	**To be completed at each scheduled facility visit: Whether the patient was screened for TB, date of screening, and screening questions used.**
	2. *Questionnaire*	*This information is abstracted onto appendix 11.1, Q28*	*This information is collected with appendix 8.1, Q53-7.*
	3. *Report/ publication*	*Proportion of ART patients screened for TB on the date of ART initiation.*	*Proportion of ART patients screened for TB on the date of ART initiation.*

**TB Suspects at ART start**	1. **Medical record**	**To be completed at each scheduled facility visit: Did the patient screen positive for TB?**	**To be completed at each scheduled facility visit: Did the patient screen positive for TB?**
	2. *Questionnaire*	*This information is abstracted onto appendix 11.1 Q28*	*This information is collected with appendix 8.1, Q53-7.*
	3. *Report*	*Proportion of ART patients (not already taking TB treatment at ART initiation) who screened positive for TB on the date of ART initiation.*	*Proportion of ART patients (not already taking TB treatment at ART initiation) who screened positive for TB on the date of ART initiation.*
**Active TB at ART start**	1. **Medical record**	**To be completed at each facility visit: "Is the patient taking TB treatment and date of TB treatment start"**	**To be completed at each facility visit: "Is the patient taking TB treatment and date of TB treatment start"**
	2. *Questionnaire*	*This information is abstracted onto appendix 11.1, Q #31.*	*Information on TB diagnosis is collected on appendix 5.1, Q#49 and appendix 8.1, Q#41*
	3. *Report*	*Proportion of ART patients taking TB treatment at ART initiation*	*Proportion of ART patients taking TB treatment at ART initiation*
**TB Treatment Outcomes**	1. **Medical record**	**To be completed for each TB patient that starts TB treatment. Patient was: Cured, Completed Treatment, Died, Treatment failure, defaulted/Lost-to-follow-up/missing, Transferred out, Treatment ongoing**	**To be completed for each TB patient that starts TB treatment. Patient was: Cured, Completed Treatment, Died, Treatment failure, defaulted/Lost-to-follow-up/missing, Transferred out, Treatment ongoing**
	2. *Questionnaire*	*Appendix 8.2, Question 19*	*Appendix 8.2, Question 19*
	3. *Report*	*Proportion of ART patents, initiating TB treatment in the 24 months before study start, who completed TB treatment or were cured.*	*Proportion of ART patents, initiating TB treatment in the study follow-up period following study start, who completed TB treatment or were cured.*
**Hospitalization rates**	1. **Medical record**	**To be completed for each ART patient at each visit. Was the patient hospitalized since the last visit, and if so, what was the date and cause?**	**To be completed for each ART patient at each visit. Was the patient hospitalized since the last visit, and if so, what was the date and cause?**
	2. *Questionnaire*	1. *To be collected from charts: Appendix 11.1, Q52*<br>2. *To be collected through cross-sectional interview for those retrospective patients who consent to an interview Appendix12, Q48*	*To be collected through interview Appendix 8.1, Q47*
	3. *Report*	*Incidence rate of hospitalization in the first 6 months of ART (events/100 person years)**	*Incidence rate of hospitalization in the first 6 months of ART (events/100 person years)**

7. Ethical issues

7.1. Informed consent

Written informed consent will be obtained prior to enrollment in the prospective cohorts and prior to interviews for patients alive and on ART in the retrospective cohorts. Information and consent forms will be read to patients in their own language. These consent forms, written in the

local language (Setswana) will be available for the patients to read. A certified translator will translate the English consent forms into the written local language. Translated forms will be back-translated into English to make sure accurate information is being conveyed. Simplicity of language used has been checked using the Microsoft Word Flesch-Kincaid readability tool. The purpose of the study and the reason for requesting their participation will be explained as well as potential risks, benefits, compensation, and time requirements. Interviewers will be trained to request consent in a manner that is free from controlling influences.

Assent from children aged 7-17 will be requested in addition to consent from their legal guardians in accordance with 45 CFR 46.408 and 50.55 (applicable for research not involving greater than minimal risk).[52]

For patients in the retrospective cohort, who have died, been lost to follow-up (LTFU) despite tracing attempts, stopped ART or been transferred to another facility, we are requesting a waiver of informed consent for medical record abstraction in accordance with 45CFR 46.116 (d). The waiver is appropriate because (1) the evaluation is retrospective in nature, involves no more than minimal risk to human subjects, and no personal identifiers will be collected; (2) the evaluation will not adversely affect the rights and welfare of the subjects; and, (3) the evaluation could not practicably be carried out without the waiver because it is not possible to track down and obtain consent from these patients in the retrospective cohort. Investigators do not believe that the rights of the patients in the retrospective cohort, who have died, stopped ART, been LTFU, or transferred by the time of study start, will be adversely affected by the abstraction process because (1) routine data has already been collected, and (2) all data collected for the study will be kept confidential. Confidentiality will be ensured as far as possible by: (1) abstracting data in a secure and private location (the study nurse's office), (2) not abstracting any easily identifiable data (e.g. name), (3) storing the abstracted data in brown envelopes in locked steel cabinets at the clinic until the time of transfer, (4) keeping the envelopes in sealed cardboard boxes during transfer to Gaborone, (5) keeping entered data on secure password-protected computers, and (6) never reporting any individual information. Investigators do not believe that the welfare of patients who have stopped ART, been LTFU, or been transferred, but who are still alive at the

time of data abstraction, will be adversely affected because no additional potentially risky study procedures will be performed for these patients.

The fourth criterion of 45CFR 46.116 (d) is to provide pertinent information to participants whenever possible. To meet this regulation, investigators will request written informed consent from persons in the retrospective cohort who are alive on ART at the time of study initiation for: (1) interview and (2) use of routinely collected data. These patients will be informed, during the consent process, that they were identified as eligible for inclusion in the study through review of existing clinic-based records (the clinic ART register- see consent forms in section 4).

For patients who are alive on ART, who are found to be TB suspects by the clinic nurse on the basis of a positive 4-symptom TB screen, consent for data abstraction and the cross-sectional interview will be requested. If the patient (or the patient's guardian) declines consent or assent for the interview, we will request verbal consent (or assent for minors) in the presence of a witness, to allow abstraction of routine data from the patient's medical record. If verbal consent is granted for data abstraction and use, this patient's retrospective data will be collected and included in the relevant analyses. If consent or assent is refused for both the interview and the data abstraction, the patient is excluded from any part of the study. Based on advice from the IRB, if a guardian of a minor aged 12 or younger gives verbal consent for use of their child's routine retrospective data, verbal assent from the young child aged <=12 will not be sought.

For patients who are alive on ART, who are not found to be TB suspects by the clinic nurse (i.e., screen negative for the 4-symptom TB screen), we are requesting a waiver of informed consent for medical record abstraction in accordance with 45CFR 46.116 (d). The waiver is appropriate because (1) the evaluation is retrospective in nature, involves no more than minimal risk to human subjects, and no personal identifiers will be collected; (2) the evaluation will not adversely affect the rights and welfare of the subjects; and, (3) the evaluation could not practicably be carried out without the waiver for several reasons including:

- Clinic nurses administer the TB screen, not study nurses, Therefore, study nurses have no contact with the retrospective patients who screen negative. If clinic nurses refer patients who screen negative for TB to the study nurse to administer consent forms to

allow data abstraction, this would disrupt the clinic flow and possibly reduce quality of care provided to screen-negative patients.

- If TB screen-negative patients were referred to the study nurse, this would inconvenience the TB-screen-negative patient, who would not benefit from any TB testing facilitated by the study nurse.

Investigators do not believe that the rights of the patients in the retrospective cohort, who are alive on ART and screen negative for TB, will be adversely affected by the abstraction process because (1) routine data has already been collected, and (2) all data collected for the study will be kept confidential. Confidentiality will be ensured as far as possible by: (1) abstracting data in a secure and private location (the study nurse's office), (2) not abstracting any easily identifiable data (e.g. name), (3) storing the abstracted data in brown envelopes in locked steel cabinets at the clinic until the time of transfer, (4) keeping the envelopes in sealed cardboard boxes during transfer to Gaborone, (5) keeping entered data on secure password-protected computers, and (6) never reporting any individual information. Investigators do not believe that the welfare of patients who are still alive at the time of data abstraction but who screen negative for the 4-symptom TB screen, will be adversely affected because no additional potentially risky study procedures will be performed for these patients.

The fourth criterion of 45CFR 46.116 (d) is to provide pertinent information to participants whenever possible. To meet this regulation, clinic nurses will continue to administer consent and provide information to the TB-screen-positive patients referred to them by the clinic nurses.

Written informed consent for the user-acceptibility surveys for GeneXpert operators will be sought as described in appendix 5.4.

7.2. Confidentiality

We do plan to collect patient's clinic registration numbers to help with: (1) returning results to patient's medical records; (2) supplementing patient interviews with data abstracted from medical records. In addition, if patients are LTFU, either from the prospective or retrospective

cohort, use of name and omang number is needed to search Botswana's national mortality database to assess vital status. For the retrospective cohort, we are requesting a waiver of informed consent under 45CFR 46.116 (d). The waiver is appropriate because (1) the evaluation is retrospective in nature, and involves no more than minimal risk to human subjects; (2) the evaluation will not adversely affect the rights and welfare of the subjects; and, (3) the evaluation could not practicably be carried out without the waiver. For the prospective cohort, current consent forms cover use of patient identifiers for tracing purposes.

All persons involved in data collection for the study (study nurses and laboratory personnel) as well as data entry personnel and study investigators will be trained on the importance of maintaining confidentiality of patients who participate in the study and will sign a document indicating their intent to maintain this confidentiality (Appendix 15).

Hard copies of the study data abstraction forms, interviews and laboratory results will be kept in locked cabinets at health facilities or laboratories prior to weekly transport to the CDC data entry location. Study forms will be transported either in locked briefcases or sealed boxes. Once data collection forms reach the CDC data entry location, they will be stored in locked steel cabinets. During data entry, all electronic data will be stored on personal computers with password-protected login screens. If data are transferred between investigators a secure PFSTP data transfer mechanism will be used. With all of these precautions, we are confident we will be able to maintain confidentiality of enrolled patients.

National guidelines for HIV care and treatment in Botswana recommend active tracing for patients considered late for an appointment to reduce loss to follow-up.[40] As part of these active tracing guidelines, collection of the patient's telephone numbers and residential addresses is needed at HIV clinic enrollment. Patients supply their contact information to the HIV care and treatment facility knowing that, if late for an appointment, active tracing procedures will be initiated. These guidelines are supported by evidence suggesting that many patients, who are late, may be late because they are ill and unable to reach the health facility; 58.8% who were late for ART appointments in Botswana in 2008 were found to be dead upon active tracing with most patients dying in the month after the missed appointment.[40] Therefore, our plans to actively trace

patients, who are late for clinic appointments, through phone calls and home visits are in line with national guidelines and should benefit most of the patients traced. To supplement the existing training of the tracing teams, during training for this study, the importance of confidentiality will be re-emphasized.

No names of the GeneXpert operators will be recorded on the user-acceptability questionnaire (appendix 5.4). Only investigators, who perform the survey, will have access to a table on a password-protected computer that links the operator’s name with the operator’s code. The operator’s code is necessary to facilitate longitudinal monitoring of user acceptability of the GeneXpert device over the study duration. Measures that the study investigators will take to preserve the confidentiality of the geneXpert operator and therefore the operator’s job security are described in detail in the Procedures section under the sub-section “user-acceptability survey” and in the consent form for the operator survey in appendix 5.4.

7.3. Benefits for enrolled patients

Patients prospectively enrolled in the study, should benefit from: TB drug resistance testing. According to MOH guidelines only TB suspects who have a history suggestive of possible TB drug resistance should receive TB drug resistance testing. All TB suspects enrolled in the study will have sputum tested for drug resistant TB using the gold standard methods of (1) line probe assay and (2) drug susceptibily testing, regardless of perceived risk of drug resistant TB. This may help some patients harboring drug resistant TB, who would otherwise not have been tested for TB drug resistance.

Patients enrolled in the retrospective cohort, if they are alive and in care, and agree to the single interview, will also benefit from TB drug resistance testing. Currently, there are no financial incentives planned for enrolled patients due to budgetary constraints.

There are no direct benefits for GeneXpert operators who take part in the user-acceptability survey.

All patients attending study clinics, whether they are enrolled in the study or not, should benefit from: (1) better screening for TB through training of health care providers and additional TB nurses (study nurses), (2) access to GeneXpert following rollout of this device.

7.4. Benefits for the program

The study should benefit the program by providing: (1) additional training in complementary TB case finding initiatives; (2) additional training in TB diagnostic procedures, including the use of Xpert; (3) 13 Xpert machines and necessary reagents; and (4) essential information about the impact and best practices for Xpert rollout nationally. Therefore, the study has the opportunity to benefit all future patients enrolled in Botswana's HIV care and treatment program.

The TB-HIV program could benefit from the information gained from the GeneXpert user-acceptability survey.

8. Logistics

8.1. Responsibilities

Funding for study procedures, which go beyond current national guidelines, (e.g. Xpert device purchase, Xpert reagents for patients enrolled in the study, additional TB case finding nurses at each of the sites) will be supplied by the US CDC, an agency funded by the US President's Emergency Plan for AIDS Relief (PEPFAR), at 17 sites. At 5 sites the African Comprehensive HIV/AIDS Partnerships (ACHAP) will purchase the Xpert devices, but CDC will recruit, fund and supervise the study nurses. However, routine program costs (such as for chest X-rays for TB suspects who are smear or Xpert negative) will be supplied by the MOH per national guidelines. Investigators from CDC Botswana, Atlanta, and the Ministry of Health have been identified and will share responsibility for approving the final protocol, allocation of funding responsibilities, supervision of the study, determination of the need for amendments, and dissemination of findings at meetings, conferences, and peer-reviewed journals. Investigators plan to create a publication guidelines document early during the study implementation phase, for review by all investigators. These guidelines will include the creation of a publication committee, which will have one representative from each participating organization. The publication guidelines and

publication committee are intended to create an efficient and harmonious publication process once data are ready for analysis.

8.2. Time table

A proposed time table for the study is illustrated on the following page.

Table 17: Proposed Timeline for XPRES (*Note that prospective study enrollment will continue for 19 months from the time of study initiation – in the figure below, this would be between May 2012 and November 2013. However, the timing of study initiation is dependent on approvals from various ethical review bodies).

	2011			2012								2013											2014
	Oct '11	Nov '11	Dec '11	*May '12	June '12	July '12	Aug '12	Sep '12	Oct '12	Nov '12	Dec '12	Jan '13	Feb '13	Mar '13	Apr '13	May '13	June '13	July '13	Aug '13	Sep '13	Oct '13	Nov* '13	
Protocol Submission to DGA ADS																							
Protocol Submission to CDC IRB and Botswana IRB																							
Study Preparation (Site assessments, training prep. Etc.)																							
Initiation of prospective enrollment - Pre-Xpert																							
Xpert machine rollout & prospective post-Xpert enrollment: Step #1																							
Step #2																							
Step #3																							
Step #4																							
Step #5																							
Step #6																							
Step #7																							
Step #8																							
Step #9																							
Retrospective Data Collection																							
Study Supervision																							
Data entry																							
Data analysis - retrospective cohort																							
Data analysis - retrospective + prospective cohorts																							
Dissemination of results																							

Key: Blue=Investigator responsibilities (protocol, study preparation, supervision, data entry, analysis, reporting results); Yellow=prospective pre-Xpert enrollment; Green=prospective post-Xpert enrollment; Red=retrospective patient enrollment; Grey=prospective patient follow-up.

8.3. Budget

	Year 1 (FY 2012)	Year 2 (FY 2013)	Year 3 (FY 2014)	Total
**PERSONNEL**				
Study Nurses (11 @ $30,000/year)	$ 385,000	$ 385,000	$ 385,000	$ 1,155,000
Additional Study Nurses (1 @ ($30,000/year)	$ 30,000	$ 30,000	$ 30,000	$ 90,000
Outreach Staff (4 @ $20,000/year)	$ 80,000	$ 80,000	$ 80,000	$ 240,000
Lab Techs (2 @ $20,000/year)	$ 40,000	$ 40,000	$ 40,000	$ 120,000
Lab Outreach Staff (3 @ $20,000/year)	$ 60,000	$ 60,000	$ 60,000	$ 180,000
**TRAVEL**				
International meetings/trainings	$ 25,000	$ 25,000	$ 25,000	$ 75,000
Local site visits/meeting/trainings	$ 50,000	$ 30,000	$ 30,000	$ 110,000
**SUPPLIES**				
Office Supplies/equipment for nurses	$ 10,000	$ 5,000	$ 5,000	$ 20,000
Courier costs	$ 15,000	$ 15,000	$ 15,000	$ 45,000
Computer laptops for nurses (11)	$ 20,000	$ -	$ -	$ 20,000
Internet costs at sites	$ 10,000	$ 10,000	$ 10,000	$ 30,000
Xpert Machine Package (11 Machines)*	$ 231,000	$ 20,000	$ 20,000	$ 271,000
Xpert Consumables/Cartridges	$ 20,000	$ 100,000	$ 5,000	$ 125,000
Xpert Training	$ 15,000	$ 10,000	$ -	$ 25,000
MGIT Testing Supplies	$ 20,000	$ 100,000	$ 5,000	$ 125,000
**OTHER**				
Site Renovations (Security, moving of portacabins)	$ 150,000	$ -	$ -	$ 150,000
Other/Unanticipated expenses	$ 120,000	$ 75,000	$ 75,000	$ 270,000
**Yearly Total**	**$ 1,281,000**	**$ 985,000**	**$ 785,000**	**$ 3,051,000**

8.4. First Amendment --- Reporting of: (1) Unanticipated problems involving risks to subjects or others, (2) continuing or serious protocol non-compliance, and (3) decentralization of certain study clinics

A*dverse events* are only a subset of unanticipated problems that might occur during research, and the term refers to any untoward medical occurrence that is temporally associated with study participation. *Adverse events* that are serious, unexpected, and at least possibly related to the study are considered to be *unanticipated problems involving risks to subjects or others*.

Unanticipated problems involving risks to subjects or others encompass any problem that is associated with a study, that was not anticipated, and that has a potential for harm. This includes, but is by no means limited to, serious *adverse events* that are unexpected and at least possibly related.

Noncompliance refers to the failure to adhere to approved study procedures, as well as failure to comply with IRB requirements, institutional policy, or applicable regulations. *Noncompliance* is considered serious when it represents a significant failure, or when it results in increased risk. Repeated occurrences of *noncompliance*, after they have been identified, are considered to be continuing, even if they are not serious.

In this study, there are several unfavorable outcomes that can occur among enrolled patients. Having HIV-infection, even if treated with ART, especially in resource-limited settings like Botswana, carries an increased mortality and morbidity risk. Previous studies have suggested that mortality could be as high as 15 deaths/100 person years in the first year of ART [40]. In our study, 18 prospectively enrolled study participants have died at a rate <5 events per 100 person-years. Several other adverse events are expected in our population of HIV-infected adults and children, including: new diagnoses of TB and other opportunistic infections, side effects from ART or TB medications, immune reconstitution inflammatory syndrome, and hospitalizations. Since these events are

expected, these events will not be considered adverse events or unanticipated problems involving risks to subjects or others.

For this study, some examples of *unanticipated problems involving risks to subjects or others, and noncompliance are listed below:*

1. Breach of confidentiality (note: this could be classified as both an *unanticipated problem involving risk to the subject and protocol noncompliance)*
2. Delay in return of positive TB sputum results to attending clinicians for newly diagnosed TB cases (note: this could be classified as an *unanticipated problem involving risk to the subject)*
3. Lost study sputum samples (note: this could be classified as both an *unanticipated problem involving risk to the subject and protocol noncompliance)*

These types of events are described in more detail below:

1. **Breach of confidentiality** – as might occur when a patient's file or Case Report Form with identifier is exposed to those unauthorized per the study protocol. Those who are authorized to have access to CRFs with patient identifier are: study nurses, medical officers, lab technicians, study investigators, and the data manager.

2. **Delay in return of TB sputum results to attending clinicians for newly diagnosed TB cases** – In XPRES, a delay to return reports of microbiologically confirmed Tuberculosis diagnosis to patients and their care providers might occur if study results fail to reach the study nurse. The delay in days might differ depending on the test and where the test was performed.

A delay would be reported in the following situations:

- If **smear microscopy** results from the peripheral lab reach the study nurse >4 days after the date of collection of the sputum sample.
- If **smear microscopy** results from the central national TB reference laboratory (NTRL) lab reach the study nurse >10 days after the date of collection of the sputum sample.

- If **Xpert** results from peripheral lab or point of care reaches the study nurse >2 days after the date of collection of the sputum sample.
- If **Culture** results from NTRL reaches the study nurse >49 days after the date of collection of the sputum sample.

3. **Lost study sample**. Any lost (unaccounted) study samples is considered an adverse event

Procedures for identifying and reporting either unanticipated risks to human subjects or protocol noncompliance will include:

- The study nurse or data manager identifies an event that might be an unanticipated event involving risk to human subjects or protocol non-compliance.
- The study nurse or data manager will report the event immediately to the study nurse or data management supervisor. The supervisor will have been trained to identify all events that constitute a possible risk to human subjects and events comprising continuing or serious non-compliance, and will report these events immediately to the in-country PI. If in doubt, the supervisor will report the event to the in-country PI.
- The PI will determine if the event is an unanticipated event involving risk to human subjects or protocol non-compliance that is serious or continuing, as defined in the document: "http://intranet.cdc.gov/od/oads/osi/hrpo/procedures/MAN.1.Unanticipated.ProblemsandAdverseEvents.pdf"
- If the event is an unanticipated event involving risk to human subjects or an incident of serious or continuing protocol non-compliance, the PI will email the report to IRB within 2 working days, and a formal report will be submitted to IRB within 2 weeks, in accordance with CDC guidelines.
- The report made within 2 days of the event, will include the following information:
  a. CDC protocol number
  b. Statement that an incident is thought to have occurred
  c. An explanation of the basis for determining that the adverse event, incident, experience, or outcome represents an unanticipated problem

d. Geographic site of occurrence

- The report made within 2 weeks will include submission to HRPO of form 0.1254 and if applicable form 0.1254S and any additional documentation. The form(s) will be submitted within this timeframe even if information is not yet complete.

Appendices 16 and 17 will help with reporting events that possibly constitute unanticipated events with possible risks to subjects or others and protocol non-compliance.

A description of MOH decentralization of study clinics

Study HIV care and treatment clinics have been decentralized by MOH since study initiation. Investigators were aware of decentralization plans before study initiation, but were unable to anticipate which study clinics would be decentralized. The decentralization process involves allowing HIV infected patients to enroll and receive study follow-up at clinics closer to their home. Therefore, the patient population that would have enrolled and received follow-up at Boswelelo clinic in Francistowm, now enrolls at both Boswelelo and Boikhutso clinic. Therefore, some prospectively enrolled patients at Boswelelo, now receive their follow-up at Boikhutso clinic. Any patient that screens positive for TB at the new clinic Boikhutso, will also have access to Xpert, when Xpert is implemented for Boswelelo. Therefore, Boikhutso clinic is in many ways an extension of the HIV clinic that started at Boswelelo.

Summary of Decentralized Locations* Supporting XPRES Patient Enrollment and Follow-up

	Study sites decentralized ARV initiation	New clinics enrolling*	Remark
1	Boswelelo clinic (Francistown district)	Boikhutso clinic	Initiated enrolling on 12-Oct-12 reporting under Boswelelo clinic

2	Nyangabwe Referral hospital (Francistown district)	Masego clinic	Initiated enrolling on 12-Mar-13 reporting under Nyangabwe Referral hospital
3	Deborah Memorial Hospital (Kgatleng district)	Morwa clinic	Initiated enrolling on 15-Jan-13 reporting under Deborah Memorial Hospital
4	Letsholathebe II Memorial hospital (Ngami- Maun District)	Sedie clinic	Initiated enrolling on 13-Mar-13 reporting under Letsholathebe II Memorial hospital

9. References

1. Ansari NA, Kombe AH, Kenyon TA, et al. Pathology and causes of death in a group of 128 predominantly HIV-positive patients in Botswana, 1997-1998. *Int J Tuberc Lung Dis* 2002; **6**(1): 55-63.
2. Ansari NA, Kombe AH, Kenyon TA, et al. Pathology and causes of death in a series of human immunodeficiency virus-positive and -negative pediatric referral hospital admissions in Botswana. *Pediatr Infect Dis J* 2003; **22**(1): 43-7.
3. Etard JF, Ndiaye I, Thierry-Mieg M, et al. Mortality and causes of death in adults receiving highly active antiretroviral therapy in Senegal: a 7-year cohort study. *AIDS* 2006; **20**(8): 1181-9.
4. Auld AF, Mbofana F, Shiraishi RW, et al. Four-Year Treatment Outcomes of Adult Patients Enrolled in Mozambique's Rapidly Expanding Antiretroviral Therapy Program. *PLoS One* 2011; **6**(4): e18453.
5. Lawn SD, Wilkinson RJ, Lipman MC, Wood R. Immune reconstitution and "unmasking" of tuberculosis during antiretroviral therapy. *Am J Respir Crit Care Med* 2008; **177**(7): 680-5.
6. Marais BJ, Graham SM, Cotton MF, Beyers N. Diagnostic and management challenges for childhood tuberculosis in the era of HIV. *J Infect Dis* 2007; **196 Suppl 1**: S76-85.
7. Boehme CC, Nicol MP, Nabeta P, et al. Feasibility, diagnostic accuracy, and effectiveness of decentralised use of the Xpert MTB/RIF test for diagnosis of tuberculosis and multidrug resistance: a multicentre implementation study. *Lancet* 2011; **377**(9776): 1495-505.
8. Shah S, Demissie M, Lambert L, et al. Intensified tuberculosis case finding among HIV-Infected persons from a voluntary counseling and testing center in Addis Ababa, Ethiopia. *J Acquir Immune Defic Syndr* 2009; **50**(5): 537-45.
9. Hermans SM, Kiragga AN, Schaefer P, Kambugu A, Hoepelman AI, Manabe YC. Incident tuberculosis during antiretroviral therapy contributes to suboptimal immune reconstitution in a large urban HIV clinic in sub-Saharan Africa. *PLoS One* 2010; **5**(5): e10527.
10. Dembele M, Saleri N, Carvalho AC, et al. Incidence of tuberculosis after HAART initiation in a cohort of HIV-positive patients in Burkina Faso. *Int J Tuberc Lung Dis* 2010; **14**(3): 318-23.
11. Moore D, Liechty C, Ekwaru P, et al. Prevalence, incidence and mortality associated with tuberculosis in HIV-infected patients initiating antiretroviral therapy in rural Uganda. *AIDS* 2007; **21**(6): 713-9.
12. Lawn SD, Kranzer K, Edwards DJ, McNally M, Bekker LG, Wood R. Tuberculosis during the first year of antiretroviral therapy in a South African cohort using an intensive pretreatment screening strategy. *AIDS* 2010; **24**(9): 1323-8.
13. Van Rie A, Westreich D, Sanne I. Tuberculosis in patients receiving antiretroviral treatment: incidence, risk factors, and prevention strategies. *J Acquir Immune Defic Syndr* 2011; **56**(4): 349-55.
14. UNAIDS. Global Report: UNAIDS Report on the Global HIV/AIDS Epidemic - 20102010. (accessed.
15. Morgan D, Mahe C, Mayanja B, Okongo JM, Lubega R, Whitworth JA. HIV-1 infection in rural Africa: is there a difference in median time to AIDS and survival compared with that in industrialized countries? *AIDS* 2002; **16**(4): 597-603.
16. World Health Organization. Antiretroviral therapy for HIV infection in adults and adolescents in resource-limited settings: towards universal access. Recommendations for a public health approach 2006 revision. **Accessed January 29, 2010:** **http://www.who.int/hiv/pub/guidelines/artadultguidelines.pdf**.
17. Braitstein P, Brinkhof MW, Dabis F, et al. Mortality of HIV-1-infected patients in the first year of antiretroviral therapy: comparison between low-income and high-income countries. *Lancet* 2006; **367**(9513): 817-24.

18. Stringer J, Zulu I, Levy J, et al. Rapid scale-up of antiretroviral therapy at primary care sites in Zambia: feasibility and early outcomes. *JAMA* 2006; **296**: 782-93.
19. Lawn SD, Harries AD, Anglaret X, Myer L, Wood R. Early mortality among adults accessing antiretroviral treatment programmes in sub-Saharan Africa. *AIDS* 2008; **22**(15): 1897-908.
20. Corbett EL, Watt CJ, Walker N, et al. The growing burden of tuberculosis: global trends and interactions with the HIV epidemic. *Arch Intern Med* 2003; **163**(9): 1009-21.
21. WHO. Global tuberculosis control, 2010. Accessed 4 September 2011 at: http://whqlibdoc.who.int/publications/2010/9789241564069_eng.pdf 2010.
22. Perkins MD, Cunningham J. Facing the crisis: improving the diagnosis of tuberculosis in the HIV era. *J Infect Dis* 2007; **196 Suppl 1**: S15-27.
23. Bruchfeld J, Aderaye G, Palme IB, Bjorvatn B, Kallenius G, Lindquist L. Sputum concentration improves diagnosis of tuberculosis in a setting with a high prevalence of HIV. *Trans R Soc Trop Med Hyg* 2000; **94**(6): 677-80.
24. Steingart KR, Ramsay A, Pai M. Optimizing sputum smear microscopy for the diagnosis of pulmonary tuberculosis. *Expert Rev Anti Infect Ther* 2007; **5**(3): 327-31.
25. Millen SJ, Uys PW, Hargrove J, van Helden PD, Williams BG. The effect of diagnostic delays on the drop-out rate and the total delay to diagnosis of tuberculosis. *PLoS One* 2008; **3**(4): e1933.
26. Behr MA, Warren SA, Salamon H, et al. Transmission of Mycobacterium tuberculosis from patients smear-negative for acid-fast bacilli. *Lancet* 1999; **353**(9151): 444-9.
27. Havlir DV, Barnes PF. Tuberculosis in patients with human immunodeficiency virus infection. *N Engl J Med* 1999; **340**(5): 367-73.
28. WHO. Multidrug and extensively drug-resistant TB (M/XDR-TB): 2010 global report on surveillance and response. Accessed 4 September 2011 at: http://whqlibdoc.who.int/publications/2010/9789241599191_eng.pdf. 2011.
29. Fischl MA, Daikos GL, Uttamchandani RB, et al. Clinical presentation and outcome of patients with HIV infection and tuberculosis caused by multiple-drug-resistant bacilli. *Ann Intern Med* 1992; **117**(3): 184-90.
30. Farmer P, Bayona J, Becerra M, et al. The dilemma of MDR-TB in the global era. *Int J Tuberc Lung Dis* 1998; **2**(11): 869-76.
31. Van Rie A, Enarson D. XDR tuberculosis: an indicator of public-health negligence. *Lancet* 2006; **368**(9547): 1554-6.
32. WHO. Rapid Implementation of the Xpert MTB/RIF diagnostic test. Accessed 3 September 2011 at: http://whqlibdoc.who.int/publications/2011/9789241501569_eng.pdf. 2011.
33. MOH. National Tuberculosis Programme Manual - Botswana. Accessed 12 September 2011 at: http://www.searchitech.org/itech?page=ff-08-02. 2011.
34. Getahun H, Kittikraisak W, Heilig CM, et al. Development of a standardized screening rule for tuberculosis in people living with HIV in resource-constrained settings: individual participant data meta-analysis of observational studies. *PLoS Med* 2011; **8**(1): e1000391.
35. Hayes R, Moulton LH. Cluster Randomized Trials. Boca Raton: CRC Press; 2009.
36. Brown CA, Lilford RJ. The stepped wedge trial design: a systematic review. *BMC Med Res Methodol* 2006; **6**: 54.
37. The Gambia Hepatitis Intervention Study. The Gambia Hepatitis Study Group. *Cancer Res* 1987; **47**(21): 5782-7.
38. Durovni B, Cavalcante SC, Saraceni V, et al. The implementation of isoniazid preventive therapy in HIV clinics: the experience from the TB/HIV in Rio (THRio) study. *AIDS* 2010; **24 Suppl 5**: S49-56.
39. Moulton LH, Golub JE, Durovni B, et al. Statistical design of THRio: a phased implementation clinic-randomized study of a tuberculosis preventive therapy intervention. *Clin Trials* 2007; **4**(2): 190-9.

40. Bisson GP, Gaolathe T, Gross R, et al. Overestimates of survival after HAART: implications for global scale-up efforts. *PLoS One* 2008; **3**(3): e1725.
41. Lawn SD, Brooks SV, Kranzer K, et al. Screening for HIV-Associated Tuberculosis and Rifampicin Resistance before Antiretroviral Therapy Using the Xpert MTB/RIF Assay: A Prospective Study. *PLoS Med* 2011; **8**(7): e1001067.
42. Bassett IV, Wang B, Chetty S, et al. Intensive tuberculosis screening for HIV-infected patients starting antiretroviral therapy in Durban, South Africa. *Clin Infect Dis* 2010; **51**(7): 823-9.
43. Lawn SD, Edwards DJ, Kranzer K, Vogt M, Bekker LG, Wood R. Urine lipoarabinomannan assay for tuberculosis screening before antiretroviral therapy diagnostic yield and association with immune reconstitution disease. *AIDS* 2009; **23**(14): 1875-80.
44. Cain KP, McCarthy KD, Heilig CM, et al. An algorithm for tuberculosis screening and diagnosis in people with HIV. *N Engl J Med* 2010; **362**(8): 707-16.
45. Hussey MA, Hughes JP. Design and analysis of stepped wedge cluster randomized trials. *Contemp Clin Trials* 2007; **28**(2): 182-91.
46. Lowrance DW, Ndamage F, Kayirangwa E, et al. Adult clinical and immunologic outcomes of the national antiretroviral treatment program in Rwanda during 2004-2005. *J Acquir Immune Defic Syndr* 2009; **52**(1): 49-55.
47. Zhang F, Dou Z, Ma Y, et al. Five-year outcomes of the China National Free Antiretroviral Treatment Program. *Ann Intern Med* 2009; **151**(4): 241-51, W-52.
48. WHO. Guidelines for intensified tuberculosis case-finding and isoniazid preventive therapy for people living with HIV in resource-constrained settings. Accessed 29 September 2011 at: http://whqlibdoc.who.int/publications/2011/9789241500708_eng.pdf. 2010.
49. Blanc FX. Significant enhancement in survival with early (2 weeks) vs. late (8 weeks) initiation of highly active antiretroviral treatment (HAART) in severely immunosuppressed HIV-infected adults with newly diagnosed tuberculosis. Eighteenth International AIDS Conference, Vienna, abstract THLBB106. 2010.
50. Song R, Menzies H, Vandebriel G, et al. Evaluation of tuberculosis screening approaches among HIV-infected children in Rwanda, 2008. 5th IAS Conference on HIV Pathogenesis and Treatment: Abstract no. TUPEB132. Available at: http://www.iasociety.org/Abstracts/A200721790.aspx 2009.
51. Samandari T, Agizew TB, Nyirenda S, et al. 6-month versus 36-month isoniazid preventive treatment for tuberculosis in adults with HIV infection in Botswana: a randomised, double-blind, placebo-controlled trial. *Lancet* 2011; **377**(9777): 1588-98.
52. UCSF. Summary Table on 45 CFR 46, Subpart D: Additional DHHS Protections for Children Involved as Subjects in Research. Available at: http://www.research.ucsf.edu/chr/guide/chrG_SpMinors.asp#4a. 2011.

10. List of Appendices – Attached as Separate Documents

1.1 Pre-Expert MOH Recommended TB Diagnostic Algorithm for Adults

1.2 Post-Expert MOH Recommended TB Diagnostic Algorithm for Adults

2 MOH Recommended TB Diagnosis Algorithm for Children under 12

3 Selected Sites and ART Patient Enrollment Characteristics

4 Information, Consent and Assent Forms at Study Enrollment

4.1 Eligibility Assessment and Consent Procedures for New HIV Clinic Enrollees

4.1.2 Study Eligibility Register

4.2.1 Information and Consent Form – Prospective Adult HIV Clinic Enrollees (≥18 years old at time of consent)

4.2.2 Information and Consent Form for Guardians of HIV Clinic Enrollees who are Minors (<18 years old at time of consent)

4.2.3 Information and Assent Form for HIV Clinic Enrollees who are Minors Aged 7-12

4.2.4 Information and Assent Form for HIV Clinic Enrollees who are Minors Aged 13-17

4.3.1 Information and Consent Form for Cross-Sectional Interview – Adult ART Enrollees (≥18 years old at time of consent)

4.3.2 Information and Consent Form for Guardians of Minors (currently <18 years old) who are Eligible for the Cross-Sectional Interview

4.3.3 Information and Assent Form for Cross-Sectional Interview Enrollees who are Minors Aged 7-12

4.3.4 Information and Assent Form for Cross-Sectional Interview Enrollees who are Minors Aged 13-17

5 Prospective Study Enrollment Questionnaire

5.1 Patient Locating Information – Kept by Study Nurse for Tracing Purposes

5.2 Specimen Collection Form (revision is separation into NTRL & Peripheral TB lab)

5.3 Prospective Study Register to Facilitate Appointment Tracking

5.4 GeneXpert Technician User Acceptance Consent Form and Survey

5.4 #2 Standardized Assessment of Workspace Cleanness or Clutter

6 Classification of TB Treatment Outcomes and Treatment Regimens for Drug-resistant TB

7 Recommended ART Regimens for Adults and Children

8.1 (8?) Follow-up Questionnaire for Patients Enrolled in Prospective Cohorts

8.2 TB Treatment Record Abstraction Form

8.3 Study Exit Form

9 Questionnaire for Patients Late for Clinic Appointments

9.1 Questionnaire to Ascertain Cause of Death

9.2 Information and Consent Form for Interview to Ascertain Cause of Death of Study Enrollee (should only read to an interviewee ≥18 years of age

10 Retrospective Study Register for Study Nurses Indicating Eligible Patients for Enrollment, ART Start Date, and Date of Next Scheduled Appointment

11.1 Adult Retrospective Data Abstraction Questionnaire (>12 years old at ART start)

11.2 Pediatric Retrospective Data Abstraction Questionnaire (≤12 at ART start)

12 Interview for Patients Enrolled Retrospectively

13 Interview for Health Care Worker at Each Participating Study Site (info sheet and questionnaire)

14 Chest X-ray Interpretation and Results Form

15 Statement of Intent to Maintain Confidentiality

16 XPRES Adverse Event Reporting Form (in clean only)

17 Protocol Violation Reporting Form (in clean only)
