# Supplementary material for: Derivation and external validation of a risk score for predicting HIV-associated tuberculosis to support case finding and preventive therapy scale-up: A cohort study
Source: PLoS Med. 2021 Sep 7;18(9):e1003739. doi: 10.1371/journal.pmed.1003739 (PMC8454974; doi:10.1371/journal.pmed.1003739)
Supplement: Attachment — Submitted filename: Response_Reviewer_comments_TBRisk_Score_July_16_2021.pdf [file pmed.1003739.s020.pdf]

The Editor
PLOS Medicine
PLOS Carlyle House, Carlyle Road
Cambridge, CB4 3DN

July 16, 2021

Dear Dr. Turner,

Re: Response to Reviewer Comments: "Risk score for predicting HIV-associated tuberculosis to support case finding and preventive therapy scale-up: A derivation and external validation cohort study using regression and machine learning approaches" (PMEDICINE-D-20-05706R2)

Thank you for the review of the manuscript and helpful feedback. Below we have addressed each comment point-by-point.

Thank you again for the review and we look forward to hearing from you,
Andrew

Andrew F. Auld MD, PhD, MSc
Country Director
U.S. Centers for Disease Control and Prevention (CDC), Malawi
Phone (mobile): +265 88 466 4257; Email: aauld@cdc.gov
Webpage: www.cdc.gov/globalhealth/countries/malawi

Requests from Editors:

We suggest some restructuring and trimming of the title: "Derivation and external validation of a risk score for predicting HIV-associated tuberculosis to support case finding and preventive therapy scale-up: A cohort study".

Response: Thank you. We agree with the proposed title and have made the changes (lines 1-3 of the marked version).

Early in the abstract, please remove "... to over 13 million PLHIV by 2021" as the context for this target appears elsewhere.

Response: Thank you. We have removed the phrase "to over 13 million PLHIV by 2021" as advised. Changes are made on line 44 of the marked version.

In the abstract, please briefly mention when the XPRES data were collected.

Response: Thank you. We have added this information on line 49 of the marked version. XPRES data collection was during 2012–2015.

Please also quote aggregate demographic details for XPRES participants.

Response: Thank you. We have added this information : “67% were female, and median age was 34” and “characteristics were similar between the derivation and validation datasets”. Changes are made on line 57.

Noting one instance in figure 5, please substitute "sex" for "gender" throughout the ms, where appropriate.

Response: Thank you. We have changed figure 5 to include the term “sex” instead of “gender”. We also changed Appendix 5 (protocol) where the word “gender” was used instead of “sex” in one location.

Noting the comments from one referee, we ask you to convert move several of the figures to supplementary information: we suggest figures 2, 4, 6, 7 and 8.

Response: Thank you. We have moved figures 2, 4, 6, and 7 to supplementary information. If possible, since figure 8 is (a) relatively simple and helps readers understand the potential for risk differentiation using the risk score, and (b) follows previous published precedent [1], we hope it is acceptable to still include figure 8 as a figure in the paper. This would be a total of five figures included, similar to recently published risk scores [1,2].

Figures 1, 3, 5, 8, and 9 are now figures 1, 2, 3, 4, and 5. By moving 4 figures to the supplementary appendices, the manuscript now has 18 appendices, up from 14.

Please adapt the attached TRIPOD checklist so that individual items are referred to by section (e.g., "Methods") and paragraph number, not by page or line numbers as these generally change in the event of publication.

Response: Thank you. We have made these changes to the TRIPOD checklist and included the revised appendix as Appendix 4.

Comment from academic editor:

I think that the authors should cite and mention an article published last year, listed below, which belongs with the list of other TB risk prediction tools in the Discussion.

"Baik Y, Rickman HM, Hanrahan CF, Mmolawa L, Kitonsa PJ, Sewelana T, et al. (2020) A clinical score for identifying active tuberculosis while awaiting microbiological results:

Development and validation of a multivariable prediction model in sub-Saharan Africa. PLoS Med 17(11): e1003420. https://doi.org/10.1371/journal. pmed.1003420."

Response: Thank you. We have added this reference on page 30 of the marked version, lines 562 – 565 as follows: "One recent TB clinical score was derived from a cohort of HIV-positive and -negative patients in South Africa who had symptoms of active TB; however, this score would not be able to detect asymptomatic active TB among PLHIV [2]."

Comments from Reviewers:

*** Reviewer #1:

Thanks authors for their great effort to improve the manucript. I am mostly satisfied with the response and revision so no further major issues needing attention. However, although the authors argued strongly I still reserve my opinion to make the paper more concise and readable by especially making the abstract and title more concise and focused on main messages (shorter) so relatively easy to follow. But it's up to the editors to decide and not a major issue.

Response: Thank you. We have followed the editor's guidance to make the title more concise. The abstract now also includes some additional suggestions from the editors.

*** Reviewer #3:

Thank you for the revision, no additional concerns.

Response: Thank you.

*** Reviewer #5:

[supportive report received]

Response: Thank you.

***

Any attachments provided with reviews can be seen via the following link:

____

In compliance with data protection regulations, you may request that we remove your personal registration details at any time. (Use the following URL: https://www.editorialmanager.com/pmedicine/login.asp?a=r). Please contact the publication office if you have any questions.

References

1. Gupta-Wright A, Corbett EL, Wilson D, van Oosterhout JJ, Dheda K, Huerga H, et al. Risk score for predicting mortality including urine lipoarabinomannan detection in hospital inpatients with HIV-associated tuberculosis in sub-Saharan Africa: Derivation and external validation cohort study. PLoS Med. 2019;16(4):e1002776. PubMed PMID: 30951533.

2. Baik Y, Rickman HM, Hanrahan CF, Mmolawa L, Kitonsa PJ, Sewelana T, et al. A clinical score for identifying active tuberculosis while awaiting microbiological results: Development and validation of a multivariable prediction model in sub-Saharan Africa. PLoS Med. 2020;17(11):e1003420. PubMed PMID: 33170838.
