# Supplementary material for: Derivation and external validation of a risk score for predicting HIV-associated tuberculosis to support case finding and preventive therapy scale-up: A cohort study
Source: PLoS Med. 2021 Sep 7;18(9):e1003739. doi: 10.1371/journal.pmed.1003739 (PMC8454974; doi:10.1371/journal.pmed.1003739)
Supplement: Attachment — Submitted filename: Response_Additional_Edits_Risk_Score_TB_paper_april_22_2021.docx [file pmed.1003739.s019.docx]

**Response to request for changes:** "Risk score for predicting HIV-associated tuberculosis to support case finding and preventive therapy scale-up: A derivation and external validation cohort study using regression and machine learning approaches"

Dear Krisztina and PLOS Medicine team,

Thank you for the opportunity to make additional changes to the paper to meet journal requirements. Please advise if we can provide additional information.

Sincerely,

Andrew

Andrew F. Auld MD, PhD, MSc

Country Director

U.S. Centers for Disease Control and Prevention (CDC), Malawi

Phone (mobile): +265 88 466 4257; Email: [aauld@cdc.gov](mailto:aauld@cdc.gov)

Dear Dr. Auld,

Thank you for submitting your manuscript entitled "Risk score for predicting HIV-associated tuberculosis to support case finding and preventive therapy scale-up: A derivation and external validation cohort study using regression and machine learning approaches" to PLOS Medicine.

Unfortunately, we have detected the following problems with your submission that will need to be corrected before we can proceed:

1. Thank you for providing a Financial Disclosure statement in the submission form. This statement is published with the article, therefore should be completed in full sentences and contain the exact wording you wish to be published.

i) State the initials, alongside each funding source, of each author to receive each grant.

**Response**: Thank you. The statement as currently recorded is the way both the President’s Emergency Plan for AIDS Relief (PEPFAR) and the US Centers for Disease Control and Prevention (CDC) would like to see the statement published. PEPFAR funds CDC country offices around the world to implement studies such as the one reported in this paper. Therefore, no specific author received a specific research grant for this study. Two recently published papers with the same wording are referenced here [1,2]. We have therefore left the proposed wording the same as follows and hope it is acceptable:

- “This research has been supported by the President’s Emergency Plan for AIDS Relief (PEPFAR) through the US Centers for Disease Control and Prevention. The funder had no role in the study design, data collection and analysis, decision to publish, or preparation of the manuscript.”

2. We do not publish any copyright or trademark symbols that usually accompany proprietary names, eg (R), (C), or TM (e.g. next to drug or reagent names). Therefore please remove all instances of trademark/copyright symbols throughout the text, including ® in S5 Appendix.

**Response**: Thank you.

- We have removed the (R) from the references section, page 46, line 905.
- We have removed the ® from S5 appendix, page 5.

3. Your Supporting Information file S6 contains branding/a logo. We are not permitted to publish this under our CC-BY 4.0 license, even with permission. We ask that you please remove or replace it.

**Response**: Thank you. We have removed the “TRIPOD” logo from the file S6.

Your paper has been sent back to enable you to make these changes. Please log in at <https://www.editorialmanager.com/pmedicine/>. Click the 'Submissions Sent Back to Author' or 'Revisions Sent Back to Author' link to access your submission. You can then make the changes requested above by clicking 'Edit Submission' from the list of Action Links. Once you have completed your edits, please 'View Submission' and then click 'Approve Submission' to resubmit.

If you have any questions or require assistance, please don't hesitate to get in touch. To help us minimize inbox volumes, we kindly ask that you not reply to this email simply to confirm you have resubmitted your revisions.

Best regards,

Krisztina Konzella

The PLOS Medicine Staff

[plosmedicine@plos.org](mailto:plosmedicine@plos.org)

**References**:

1.	Auld AF, Agizew T, Mathoma A, Boyd R, Date A, Pals SL, et al. Effect of tuberculosis screening and retention interventions on early antiretroviral therapy mortality in Botswana: a stepped-wedge cluster randomized trial. BMC Med. 2020;18(1):19. PubMed PMID: 32041583.

2.	Auld AF, Fielding K, Agizew T, Maida A, Mathoma A, Boyd R, et al. Risk scores for predicting early antiretroviral therapy mortality in sub-Saharan Africa to inform who needs intensification of care: a derivation and external validation cohort study. BMC Med. 2020;18(1):311. PubMed PMID: 33161899.
